# Anticancer effects of Erzhimaoling decoction in high-grade serous ovarian cancer in vitro and in vivo

**DOI:** 10.1186/s40001-024-01968-4

**Published:** 2024-08-05

**Authors:** Li Yang, Jingfang Liu, Jiejie Zhang, Feng Shao, Yanlu Jin, Jie Xing, Heran Zhou, Aijun Yu

**Affiliations:** 1 Department of Gynecological Oncology, Zhejiang Cancer Hospital, No. 1 Banshan East Road, Hangzhou, 310022 Zhejiang China; 2 Department of Oncology, Hangzhou TCM Hospital Affiliated to Zhejiang Chinese Medical University, No. 453 Stadium Road, Hangzhou, 310007 Zhejiang China

**Keywords:** Traditional Chinese Medicine, METTL3, METTL14, KRT8, FAS, SKOV3, Apoptosis

## Abstract

**Background:**

High-grade serous ovarian cancer (HGSOC) is a common gynecologic malignancy with a poor prognosis. The traditional Chinese medicine formula Erzhimaoling decoction (EZMLD) has anticancer potential. This study aims to elucidate the anticancer effects of EZMLD on HGSOC in vitro and in vivo.

**Materials and methods:**

EZMLD-containing serum was prepared from Sprague–Dawley rats for treating SKOV3 ovarian cancer cells at varying concentrations for 24 h and 48 h to determine the IC_50_. Concentrations of 0%, 5%, and 10% for 24 h were chosen for subsequent in vitro experiments. The roles of METTL3 and METTL14 in SKOV3 cells were explored by overexpressing these genes and combining EZMLD with METTL3/14 knockdown. Investigations focused on cell viability and apoptosis, apoptosis-related protein expression, and KRT8 mRNA m6A modification. For in vivo studies, 36 BALB/c nude mice were divided into six groups involving EZMLD (6.75, 13.5, and 27 g/kg) and METTL3 or METTL14 knockdowns, with daily EZMLD gavage for two weeks.

**Results:**

In vitro, EZMLD-containing serum had IC_50_ values of 8.29% at 24 h and 5.95% at 48 h in SKOV3 cells. EZMLD-containing serum decreased SKOV3 cell viability and increased apoptosis. EZMLD upregulated METTL3/14 and FAS-mediated apoptosis proteins, while downregulating Keratin 8 (KRT8). EZMLD increased KRT8 mRNA m6A methylation. METTL3/14 overexpression reduced SKOV3 cell viability and increased apoptosis, while METTL3/14 knockdown mitigated EZMLD's effects. In vivo, EZMLD suppressed SKOV3 xenografts growth, causing significant apoptosis and modulating protein expression.

**Conclusions:**

EZMLD has therapeutic potential for ovarian cancer and may be considered for other cancer types. Future research may explore its broader effects beyond cell apoptosis.

**Supplementary Information:**

The online version contains supplementary material available at 10.1186/s40001-024-01968-4.

## Background

Epithelial ovarian cancer accounts for nearly 90% of all ovarian cancers and 2.5% of all female tumors [1]. High-grade serous ovarian cancer (HGSOC) is the most common and deadliest type, accounting for 60–80% of ovarian cancer cases [2]. Early diagnosis of ovarian cancer is highly challenging due to its subtle symptoms. Approximately 70% of patients are diagnosed at an advanced stage, with a 5-year survival rate of less than 30% [3, 4]. The preferred treatment approach to improve survival in HGSOC involves tumor debulking surgery along with multiple cycles of chemotherapy and targeted therapy. However, the overall effectiveness of this regimen remains suboptimal, as 60% to 70% of patients experience relapse, even in cases where initial treatment is effective [5]. Therefore, there is a pressing need for potent therapeutic agents to address HGSOC more effectively.

Traditional Chinese medicine (TCM) has shown significant efficacy in treating various cancers in recent years [6]. According to TCM theories, ovarian cancer belongs to the categories of ‘Zhengjia’ and ‘Jiju’. It is characterized by underlying conditions of spleen and kidney deficiency, with the observable manifestation being accumulation of phlegm and stasis. Ovarian cancer often occurs before or after menopause, caused by reduced spleen and kidney efficiency. This leads to the formation of pathological substances such as phlegm, stasis, dampness, and toxicity. Over time, these substances accumulate and aggregate into nodules. Therefore, treatment for ovarian cancer includes tonifying and replenishing the kidneys, invigorating the spleen, and eliminating dampness.

Erzhimaoling decoction (EZMLD) is a formula commonly used in ovarian cancer patients with kidney–spleen deficiency. Its formulation is based on the classic herbal formula ‘Er Zhi Wan’ (from ‘Fu Shou Jing Fang’). The basic composition of EZMLD includes Nv Zhen Zi (*Ligustrum lucidum* W.T.Aiton, Oleaceae), Mo Han Lian (*Eclipta prostrata* (L.) L., Asteraceae), Bai Zhu (*Atractylodes macrocephala* Koidz., Asteraceae), Bai Shao (*Paeonia lactiflora* Pall., Paeoniaceae), Fu Ling (*Poria cocos* (Schw.) Wolf, Polyporaceae), Mao Ren Shen (*Actinidia valvata* Dunn, Actinidiaceae), Zhe Bei Mu (*Fritillaria thunbergii* Miq., Liliaceae), and Gui Zhi (*Cinnamomum cassia* J.Presl, Lauraceae). Being a mixture of these drugs, EZMLD effectively tonifies the kidneys, strengthens the spleen, and disperses stagnation to resolve masses. In our team’s retrospective study conducted in 2020, encompassing 45 cases of postoperative ovarian cancer, patients primarily treated with EZMLD exhibited a median progression-free survival (PFS) of 25 months. This was significantly better than that of ovarian cancer patients who did not receive TCM treatment, indicating a notable effect on the extension of PFS. However, the mechanism still needs to be clarified and warrants further investigation.

*N*6-methyladenosine (m6A) is the most abundant epitranscriptomic modification in eukaryotic mRNA, involving methylation at the N6 position of adenosine (A) in RNA [7]. M6A methylation is a dynamic and reversible modification process primarily regulated by m6A methyltransferases (writers), m6A demethylases (erasers), and m6A-binding proteins (readers) [8]. The writers include METTL3, METTL14, and WTAP. METTL3 and METTL14 form a stable heterodimer and are the two core components of the methyltransferase complex [9]. The erasers are made of ALKBH5 and FTO. YTHDFs (YTHDF1-3) and YTHDCs (YTHDC1-2) are two subtypes that form the YTH domain family proteins, which mainly comprise m6A-binding proteins, also known as ‘readers’. M6A primarily affects gene expression by regulating mRNA splicing, translation, degradation, and stability [10]. A recent study indicates that m6A methylation plays a crucial and multifaceted role in the development and progression of ovarian cancer [11].

In this study, we investigated the regulatory mechanism of the METTL3 and METTL14 methyltransferases, which mediate m6A methylation modification of KRT8 to influence the FAS apoptosis signaling pathway. Our focus was on elucidating the impact of EZMLD on m6A methylation modification of KRT8 in ovarian cancer cells and its subsequent role in regulating the FAS apoptosis signaling pathway. Ultimately, our findings confirm that EZMLD induces apoptosis in ovarian cancer cells through the mechanism of METTL3 and METTL14 methyltransferases, which mediate KRT8 m6A methylation modification. This study solidifies the foundation for potential clinical trials using EZMLD in treating ovarian cancer.

## Materials and methods

### Cell line and cell culture

The established human ovarian cancer cell line SKOV3 (#HTB-77™, RRID: CVCL_0532) was obtained from the American Type Culture Collection (ATCC). Cells were cultured in Dulbecco’s modified Eagle medium (#11965092, DMEM, Thermo Scientific, USA) supplemented with 10% fetal bovine serum (#13011-8611, Sijiqing Co. Ltd., China) and 1% penicillin/streptomycin (#15140148, Gibco, USA) in a cell incubator at 37 °C in a humidified atmosphere with 5% CO_2_.

### Drug

The basic formula for EZMLD is Nv Zhen Zi 15 g, Mo Han Lian 15 g, Bai Zhu 12 g, Bai Shao 12 g, Fu Ling 15 g, Mao Ren Shen 15 g, Zhe Bei Mu 12 g, and Gui Zhi 9 g. All drugs were provided by the Pharmacy of the Zhejiang Cancer Hospital (Zhejiang, China). All herbs were immersed in 6000 mL of purified water for 30 min and boiled twice for 2 h each time. After cooling, the decoction was filtered, and the collected liquid was concentrated to different volumes and concentrations (0.675, 1.35, 2.25, 2.7 g/mL) for subsequent animal experiments at 60 °C in a water bath using a vacuum concentrator. After the extraction process, EZMLD was stored at 4℃ for future applications.

### Animals and their care

Six male SPF-grade Sprague-Dawley rats (180–200 g) and 36 female BALB/c SPF-grade nude mice (4–5 weeks old) were acquired from Shanghai Slac Laboratory Animal Co., Ltd., China. Rats were used to prepare drug-containing and control serums for in vitro cell experiments. Nude mice were used to evaluate the in vivo anti-cancer effects of EZMLD. All animals were cared for according to the Guidance Suggestions for the Care and Use of Laboratory Animals (Ministry of Science and Technology of China) and housed at the Zhejiang Ying Yang Pharmaceutical R&D Center, China (licensed under SYXK(Zhe)2021-0033). Animals were kept in individually ventilated cages (2 rats or 4 mice per cage) in an environment with 20–26 °C temperature, 50%–60% humidity, a 12 h light/dark cycle, and 15–20 air changes per hour. Daily monitoring ensured humane treatment. Euthanasia was conducted humanely with intraperitoneal injection of 2% sodium pentobarbital (150 mg/kg for rats, 200 mg/kg for mice) after reaching humane endpoint criteria such as prostration, skin lesions, significant weight loss, respiratory distress, epistaxis, circling behavior, or decreased body temperature [12]. All the procedures on animals were approved by the Laboratory Animal Management and Ethics Committee of Zhejiang Ying Yang Pharmaceutical R&D Center (approval no. ZJEY-20230220-01, 2023-2-20).

### Preparation of drug-containing serum

After a 3-day acclimatization period with standard feed, the experimental rats were randomly assigned to two groups: a blank control group and an EZMLD treatment group, each comprising three rats. The clinical human dose of EZMLD is calculated as 105 g/day. To adapt this dose for rats, we used a conversion formula of the body surface area coefficient (rat to human surface area ratio = 0.018). Therefore, the rat dosage for creating drug-containing serum was determined as follows: 105 g/day (human dosage) × 0.018/0.2 kg (estimated rat weight) × 5 (serum dilution coefficient) = 47.5 g/kg/day. The serum dilution coefficient was established based on the literature [13, 14] and preliminary experiments. Thus, the drug treatment group received gavage administration at a dose of 45 g/kg/day, while the blank group received an equivalent volume of saline. Using EZMLD at the corresponding concentration [45 g/kg × 0.2 kg (estimated rat weight)/4 mL (estimated daily dose) = 2.25 g/mL], both groups were administered twice daily, in the morning and evening over three consecutive days, the volume of EZMLD was calculated as: 10 mL/kg × real rat weight (kg). On the third evening, a 12 h fasting period was implemented (while water remained accessible). Rats were anesthetized by intraperitoneal injection with 2% sodium pentobarbital (50 mg/kg). Blood samples were obtained from the abdominal aorta, centrifuged, and serum was collected. Serum collected was filtered through a 0.22 μm filter membrane, followed by heat inactivation at 56 °C for 30 min. Subsequently, the processed serum samples were stored at − 80 °C until further utilization. The rats were then euthanized under deep anesthesia with intraperitoneal injection of 2% sodium pentobarbital (150 mg/kg).

### Plasmid transfection

METTL3 or METTL14 overexpression was achieved by cloning full-length sequences into pcDNA3.1 (+) vectors, provided by the Zhejiang Ying Yang Pharmaceutical R&D Center (Zhejiang, China). Plasmid transfection in SKOV3 cells was performed using Lipofectamine 2000 (#11668019, Invitrogen, USA). The day before transfection, cells were detached and 1 × 10^6^ cells were seeded in each well of a 6-well plate. On the day of transfection, cell confluence reached 70–90%. For transfection, 4 µg of plasmids was first diluted in 250 µL of Opti-MEM per well. This was followed by adding 10 µL of Lipofectamine 2000, previously mixed with 250 µL of Opti-MEM for each well, to the diluted plasmids. The mixture was incubated at room temperature for 30 min to form DNA–lipofectamine complexes. Concurrently, the growth medium in the wells was replaced with Opti-MEM. The prepared DNA–lipofectamine complexes were then added to each well. After 4 h, the complexes were removed and replaced with DMEM. After transfection, cells were incubated at 37 °C in a CO_2_ incubator for 24–48 h before proceeding with the assays.

### Lentiviral vector transduction with short hairpin RNA

Lentiviral vectors expressing specific short hairpin RNA (shRNA) targeting METTL3 (5′-GGTTCGTTCCACCAGTCATAA-3′) or METTL14 (5′-GCTGGACTTGGGATGATATTA-3′), along with a puromycin resistance gene, were provided by the Zhejiang Ying Yang Pharmaceutical R&D Center. 293T cells were seeded at a density of 1 × 10^4^ cells per well in 96-well plates. After 24 h, cells were transfected with the lentiviral vector expression construct using Lipofectamine 2000. The lentiviral particles were harvested after 48 h, filtered, and used to infect SKOV3 cells. This was followed by puromycin screening to generate stable cell lines with METTL3 or METTL14 knockdown.

### Cell viability assay

Cell Counting Kit-8 (CCK-8) (#CK04, Dojindo Laboratories, Japan) was used to measure cell viability after EZMLD treatment. Briefly, 15,000 cells per well in 96-well plates with 100 μL maintenance medium were seeded and cultured overnight, and cells were treated with different concentrations of EZMLD for 24 h and 48 h. The medium was replaced with 180 μL fresh medium and 20 μL CCK-8 solution in each well and incubated at 37 °C for 2 h. The number of viable cells was assessed by measuring the absorbance at 450 nm with a Microplate Reader (BioTek Instruments, USA). The viability of cells in the experimental group was measured against an equivalent number of cells in the control group. IC_50_ values were calculated using Graphpad Prism 8 software (GraphPad Software Inc., USA).

### Apoptosis assay

SKOV3 cells (1.2 × 10^6^ cells/well) were seeded in six-well plates, cultured overnight, and then treated with blank serum or EZMLD for 24 h. Cells were harvested, washed twice with cold PBS, and resuspended at a density of 1 × 10^6^ cells/mL. 500 μL of binding buffer was added, followed by centrifugation to remove the supernatant. Subsequently, 100 μL of binding buffer (#556547, BD Biosciences, USA) was added, mixed well, and stained with 5 μL of Annexin V-FITC (#556547, BD Biosciences, USA) and 10 μL of PI (#556547, BD Biosciences, USA) in the dark at room temperature for 15 min. Finally, 400 μL of binding buffer was added, and cell apoptosis was measured using NovoCyte flow cytometer (Agilent Technologies, USA). The gating strategy was as follows: First, the locations of cell clusters were identified based on cell size (FSC-H) and internal complexity (SSC-H). Most cells were clustered in the middle, which was identified as the target cell cluster. Cell debris was excluded from the gate. For the target cell population, the axes were changed to Annexin V-FITC and PE channels. The position of the negative cell population was established using the blank control. The vertical axis was defined by Annexin V-FITC staining alone, and the horizontal axis was determined by PI staining alone. Ultimately, the position of the gate was established. The experiment was repeated three times.

### Methylated RNA immunoprecipitation sequencing (MeRIP-seq)

Methylated RNA immunoprecipitation sequencing, using methods previously described [15], revealed changes in m6A methylation of KRT8 in HGSOC. Three HGSOC tissues and three normal ovarian tissues were collected during surgeries at Zhejiang Cancer Hospital in 2021 and stored in biospecimen repository. Immediately after surgical excision, samples were snap-frozen in liquid nitrogen and stored at − 80 °C until research use. Normal ovarian tissues were obtained from patients undergoing surgery for uterine leiomyomas. None of the patients had received chemotherapy or radiotherapy prior to surgery. Each participant provided written informed consent. This study was approved by the Institutional Review Board of the Zhejiang Cancer Hospital (approval no. IRB-2022-781, 2022-12-27).

In brief, total RNA was isolated from the tissues using TRIzol reagent (#15596018CN, Invitrogen, USA) and assessed for purity and integrity with NanoDrop ND-1000 (NanoDrop, USA) and Bioanalyzer 2100 (Agilent, USA). Following RNA extraction, poly(A) RNA was purified and fragmented, then subjected to m6A immunoprecipitation using a specific antibody. The immunoprecipitated RNA was reverse transcribed, followed by cDNA synthesis and preparation for sequencing. Sequencing was performed on an Illumina Novaseq™ 6000 platform (LC-Bio Technology CO., Ltd., Hangzhou, China). Adaptor sequences and low-quality reads were removed using Cutadapt and in-house Perl scripts. The high-quality reads were aligned to the human genome using HISAT2, and m6A peaks were identified and analyzed using exomePeak and visualized with IGV. Motif analysis was performed using MEME and HOMER. Peak annotations and intersections were conducted with ChIPseeker. Gene expression levels were quantified using StringTie and differentially expressed genes and methylation peaks were identified using edgeR and the Poisson test, respectively.

### In vivo tumor growth assay

After a week of acclimatization in a pathogen-free environment, the 36 nude mice were divided into six groups, with six mice in each group according to the research plan (Table 1). Dosage for mice was calculated using the body surface area coefficient conversion method (the body surface area ratio of mice to humans = 0.0026). This was determined as follows: 105 g/day (human dosage) × 0.0026/0.02 kg (estimated mice weight) = 13.65 g/kg/day [16]. Based on this calculation, we established three dose levels for the experiments: low (6.75 g/kg/day), medium (13.5 g/kg/day), and high (27 g/kg/day). The corresponding concentrations of the decoction for each dose level were calculated as follows: for the low-dose group, 6.75 g/kg × 0.02 kg (estimated mice weight)/0.2 mL (estimated daily dose) = 0.675 g/mL; for the medium-dose group, 13.5 g/kg × 0.02 kg/0.2 mL = 1.35 g/mL; and for the high-dose group, 27 g/kg × 0.02 kg/0.2 mL = 2.7 g/mL. The volume of EZMLD was calculated as: 10 mL/kg × real mice weight (kg).

**Table 1:** Tumor sizes of nude mice (means ± SD, *n* = 6)

Groups	Tumor volume (mm^3^)				
	D0	D3	D6	D9	D12
SKOV3 cell nude mouse xenografts treated with saline	97.54 ± 7.40	233.90 ± 20.00	458.40 ± 40.02	804.01 ± 62.70	1341.12 ± 105.60
SKOV3 cell nude mouse xenografts + 6.75 g/kg EZMLD^a^	100.54 ± 6.23	207.88 ± 17.45	385.09 ± 37.21^▴▴^	675.93 ± 65.18^▴▴^	1121.20 ± 107.39^▴▴^
SKOV3 cell nude mouse xenografts + 13.5 g/kg EZMLD^b^	98.73 ± 5.78	185.03 ± 13.66^▴▴^	339.27 ± 26.89^▴▴^	583.64 ± 44.90^▴▴^	969.89 ± 73.05^▴▴^
SKOV3 cell nude mouse xenografts + 27 g/kg EZMLD^c^	104.17 ± 6.08	156.14 ± 13.91^▴▴^	288.71 ± 26.86^▴▴^	475.42 ± 36.35^▴▴^	778.38 ± 62.94^▴▴^
Nude mouse xenografts with METTL3 knockdown + 27 g/kg EZMLD^c^	102.21 ± 7.37	173.53 ± 9.25^▴▴^	352.09 ± 32.76^▴▴^*	601.22 ± 63.44^▴▴^**	998.90 ± 78.86^▴▴^**
Nude mouse xenografts with METTL14 knockdown + 27 g/kg EZMLD^c^	98.31 ± 5.66	169.82 ± 13.83^▴▴^	374.48 ± 26.71^▴▴^**	571.31 ± 34.43^▴▴^*	951.33 ± 45.05^▴▴^*

EZMLD was administered once daily by gavage for 2 weeks when tumors grown subcutaneously in nude mice reached about 100 mm^3^, tumor diameter and volume were measured/calculated every 3 days. Compared to SKOV3 cell nude mouse xenografts treated with saline, ^▴^*P* < 0.05, ^▴▴^*P* < 0.01; Compared to SKOV3 cell nude mouse xenografts + 27 g/kg EZMLD, *P < 0.05, **P < 0.01. ^a^ 0.675 g/mL, ^b^ 1.35 g/mL, ^c^ 2.7 g/mL.

SKOV3 cells were harvested in their logarithmic growth phase and suspended at a concentration of 1 × 10^8^ cells/mL. Each mouse received a subcutaneous injection of 0.2 mL of cell suspension in the right axillary region. Approximately 2 to 3 weeks later, when measurable tumors (around 100 mm^3^) appeared at the injection site, mice received oral gavage administration of EZMLD with the corresponding concentrations and volumes according to grouping once daily for two consecutive weeks. The tumor diameter was measured every three days, totaling five measurements throughout the study. At the end of the animal experiment, the mice were euthanized under deep anesthesia with intraperitoneal injection of 2% sodium pentobarbital (200 mg/kg), samples were taken, photos were captured, and a tumor volume chart was plotted. Tumor volume was calculated using the formula: Volume = long diameter × short diameter^2^/2.

### Hematoxylin and eosin staining

Hematoxylin and eosin (HE) staining was utilized to observe the pathological changes in mouse tumor tissues. Briefly, paraffin sections of tumor tissues were deparaffinized, stained with hematoxylin (#H3136, Sigma–Aldrich, Germany) and eosin (#E4009, Sigma–Aldrich, Germany), and dehydrated before examining the slide under a microscope. The tissue injury was assessed based on a scoring system using the percentage of the damaged area, where 0 indicated that there was no damage, 1 represented less than 25% damage, 2 indicated damage between 25% and less than 50%, 3 denoted damage between 50% and less than 75%, and 4 signified damage equal to or exceeding 75%.

### Terminal deoxynucleotidyl transferase d-UTP nick end labeling (TUNEL) assay

TUNEL staining was used to detect cell apoptosis in tumor tissues of mice from various groups. Paraffin sections were dewaxed at room temperature and incubated at 37℃ for 22 min with a proteinase K working solution. After adding the permeabilization working solution and setting it at room temperature for 20 min, the appropriate amount of TDT enzyme, dUTP, and the buffer mixture of the TUNEL kit (#11684795910; Roche, Switzerland) was added to the slides and incubated at 37 °C for 2 h. Then, a 4′, 6′-diamidino-2-phenylindole (DAPI) (#ab104139, Abcam, UK) stain was added and the slides mounted. Finally, the images were captured under a fluorescence microscope. Finally, the images were captured under a fluorescence microscope (magnification, ×200), and the percentage of apoptotic cells was calculated.

### Western blot analysis

After designated treatment, SKOV3 cells and 100 mg of tumor tissue were harvested and lysed using RIPA lysis buffer (#P0013B, Beyotime, China). Protein concentration in the lysates was measured with a BCA assay kit (#P0010, Beyotime, China). 50 μg of protein samples was separated by SDS-PAGE and transferred to PVDF membranes (GE Healthcare Life, USA). These membranes were blocked with 5% fat-free milk and subsequently incubated with primary antibodies. The primary antibodies used were against METTL3 (rabbit, 1:1000, #ab195352, RRID: AB_2721254, Abcam, UK), METTL14 (mouse, 1:1000, #ab220031, RRID: AB_2664841, Abcam, UK), KRT8 (rabbit, 1:1000, #ab53280, RRID: AB_869901, Abcam, UK), FAS (rabbit, 1:1000, #ab133619, RRID: AB_2940837, Abcam, UK), FADD (rabbit, 1:1000, #ab108601, RRID: AB_10864812, Abcam, UK), phosphorylated FADD (p-FADD) (rabbit, 1:1000, #ab68475, RRID: AB_11155500, Abcam, UK), Caspase 8 (rabbit, 1:1000, #ab32397, RRID: AB_725956, Abcam, UK), cleaved Caspase 3 (rabbit, 1:1000, #ab32042, RRID: AB_725947, Abcam, UK), and GAPDH (rabbit, 1:10,000, #10494-1-AP, RRID: AB_2263076, Proteintech, USA). This was followed by incubation with horseradish peroxidase (HRP)-conjugated goat anti-rabbit (1:6000, #7074, RRID: AB_2099233, Cell Signaling Technology, USA) or anti-mouse (1:6000, #7076, RRID: AB_330924, Cell Signaling Technology, USA) secondary antibodies. Immunoreactivity was detected using the ECL Western blot detection kit (GE Healthcare Life, USA).

### Global m6A methylation quantification assay

The mRNA global m6A levels in SKOV3 cells and xenograft tumors in nude mice were assessed using the EpiQuik m6A RNA methylation quantification kit (#EPT-P-9005-48, Epigentek, USA). Briefly, 200 ng of RNA was bound to strip wells with RNA binding solution and then incubated with the capture antibody at room temperature for 1 h before. After washing away unbound antibodies, a detection antibody was applied. The development solution was then added and absorbance at 450 nm was measured using a CMaxPlus microplate reader (Molecular Devices, USA) for colorimetric detection. Finally, m6A levels were calculated based on a standard curve.

### Methylated RNA immunoprecipitation-quantitative reverse transcription PCR (MeRIP-qRT-PCR)

An EpiQuik™ CUT&RUN m6A RNA Enrichment (MeRIP) Kit 213 (#P-9018, Epigentek, USA) was used according to the manufacturer’s protocols to detect changes in KRT8 m6A mRNA methylation in various cell groups and tumor tissues of nude mice. In brief, total RNA was isolated and fragmented into approximately 200 nucleotides, immunoprecipitated with m6A antibody or IgG control bound to Magna ChIP Protein A/G Magnetic Beads. The m6A-precipitated RNA was released with elution buffer and purified by ethanol before RT-qPCR was conducted. The primer pairs of human-KRT8, ATGTTTGCGGAATGAATGGG (forward primer) and ATCCTCGTACTGTGCCTTGA (reverse primer), human-β-actin, CATGTACGTTGCTATCCAGGC8 (forward primer) and CTCCTTAATGTCACGCACGAT (reverse primer), mouse-KRT8, TCCGAGATGAACCGCAACAT (forward primer) and TGCTCATGTTCTGCATCCCA (reverse primer) and mouse-β-actin ACTGCCGCATCCTCTTCCT (forward primer) and TCAACGTCACACTTCATGATGGA (reverse primer) were used.

### Immunohistochemical staining

Immunohistochemistry was used to evaluate the expression levels of METTL3 and METTL14 in tumor tissues of nude mice. Tumor tissue paraffin sections were deparaffinized and rehydrated. Endogenous peroxidase activity was blocked using hydrogen peroxide. This was followed by heat-induced antigen retrieval in Tris–EDTA buffer. The sections were then incubated overnight at 4 °C with primary antibodies directed against METTL3 (1:500, #ab195352, RRID: AB_2721254, Abcam, UK) and METTL14 (1:200, #48699, RRID: AB_3097700, Cell Signaling Technology, USA). This step was succeeded by incubation with HRP-conjugated goat anti-rabbit (1:2000, #ab6721, RRID: AB_955447, Abcam, UK) secondary antibody at 37 °C. Visualization of the antigens was achieved using diaminobenzidine, and the results were documented with a standard light microscope. Quantification of the average optical density (AOD) values of stained areas was performed using Image-Pro Plus 6.0 software (Media Cybernetics, USA).

### Statistical analysis

Statistical analysis was performed using SPSS 16.0 software. Shapiro–Wilk and Levene’s tests were used to check normality and homogeneity of variance. Independent samples *t* test or one-way analysis of variance (ANOVA) was used followed by Tukey’s multiple comparison tests to compare controls and treated groups. The significance level was set at *α* = 0.05. All data are presented as means ± standard deviation (SD), with *P* < 0.05 indicating statistical significance.

## Results

### EZMLD inhibits cell viability by inducing cell apoptosis

**Fig. 1:**
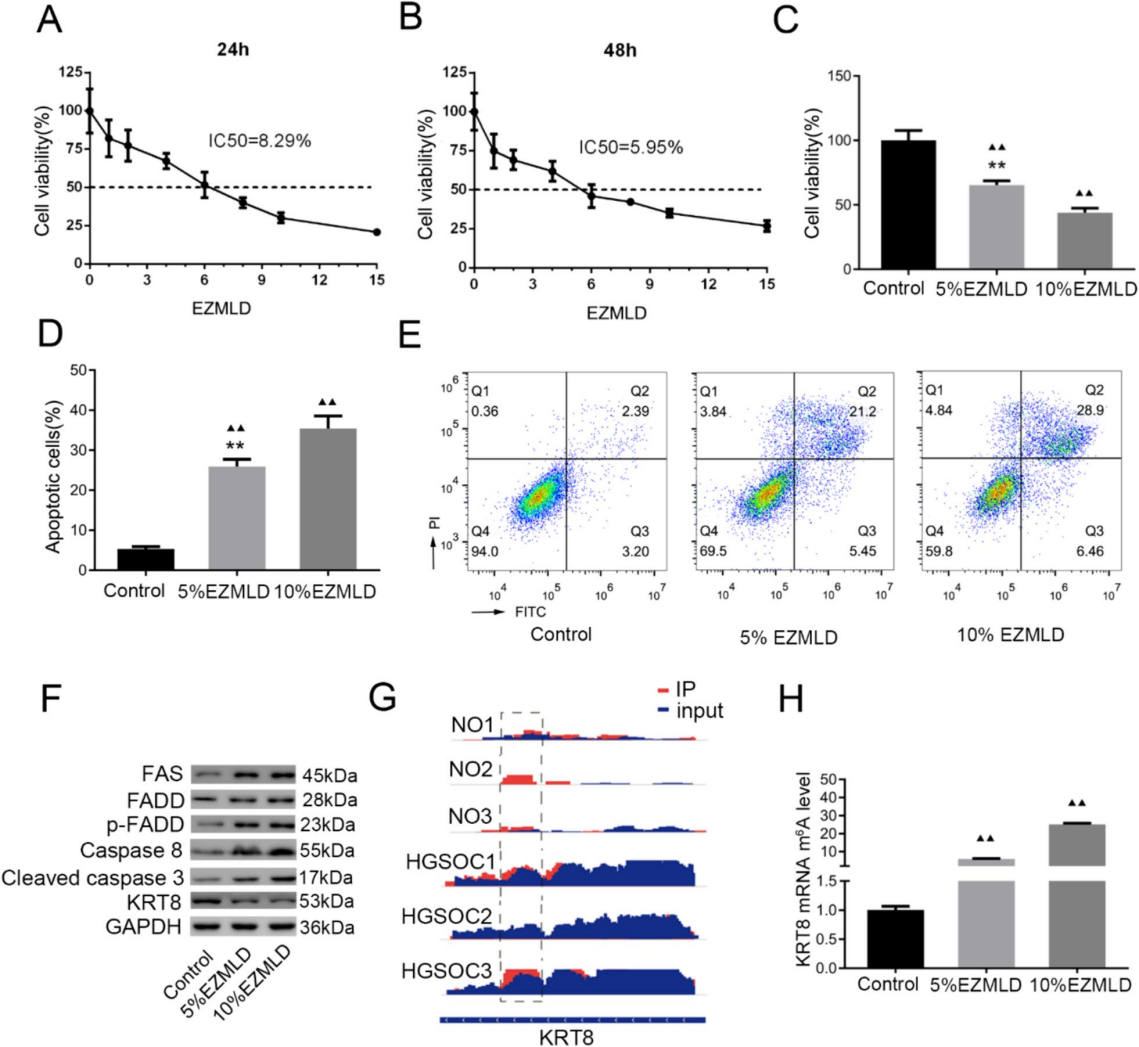
EZMLD inhibited cell viability, induced cell apoptosis, and upregulated KRT8 mRNA m6A methylation modification levels. **A** SKOV3 cells were treated with different concentrations of EZMLD for 24 h. **B** SKOV3 cells were treated with different concentrations of EZMLD for 48 h. IC_50_ was calculated. **C** EZMLD inhibited the cell viability of SKOV3 cell line. Cell viability was determined by the CCK8 assay. Error bars = 95% CIs. **D** Quantitative analysis of apoptotic cells. **E** EZMLD-induced cell apoptosis examined by flow cytometry. **F** Western blot analysis of apoptosis-associated proteins. **G** Status of the KRT8 mRNA m6A in HGSOC patients and normal ovary. NO, normal ovary. **H** MeRIP-qRT-PCR of KTR8 mRNA m6A levels. Data are means of three independent experiments ± SD. ^▴▴^Compared to control group, *P* < 0.01; ^**^Compared to 10% EZMLD, *P* < 0.01

To examine the anticancer effect of EZMLD on ovarian cancer cells, SKOV3 cells were treated with serum containing EZMLD at different concentrations, and cell viability was assessed using the CCK-8 assay. The IC_50_ of EZMLD-containing serum in SKOV3 cells for 24 h and 48 h was 8.29% and 5.95%, respectively (Fig. 1A, B). Therefore, we treated SKOV3 cells with 5% and 10% EZMLD-containing serum for 24 h, and assessed cell viability. As shown in Fig. 1C, SKOV3 cell viability decreased markedly in the 5% and 10% EZMLD-containing serum groups. The inhibitory effect on cells intensified with higher EZMLD concentrations compared to the control group.

To elucidate the potential mechanism underlying the anticancer effects of EZMLD, we investigated the triggering of apoptosis following EZMLD treatment. After treatment with different concentrations of EZMLD-containing serum for 24 h and buffer solution, SKOV3 cells were stained with Annexin V and PI, and the percentage of apoptotic cells was assessed by flow cytometry. The proportion of total apoptotic cells, including early (Annexin V positive) and late (Annexin V and PI positive) apoptotic cells, increased with the rise in EZMLD concentrations in SKOV3 cells (Fig. 1D, E).

To explore the potential mechanism, we analyzed the major proteins associated with apoptosis by Western blot. We found that after treatment with EZMLD, the expression of FADD was slightly altered, but the expression levels of FAS, phospho-FADD (Ser194) (p-FADD), Caspase-8, and cleaved Caspase-3 increased significantly (Fig. 1F). Previous studies reported that KRT8, a major intermediate filament protein, can resist FAS-mediated apoptosis [17, 18]. So, we also detected the expression of KRT8 and found that the protein expression level of KRT8 was significantly down-regulated (Fig. 1F).

### M6A methylation status of KRT8 mRNA

Our research used MeRIP-seq to detect m6A levels in 3 pairs of high-grade serous ovarian cancer and normal ovarian tissues. In ovarian cancer patients, we found a hypomethylated m6A state of the KRT8 mRNA (Fig. 1G). We speculated that the methylation status of KRT8 might play a role in the antitumor mechanism of EZMLD. Thus, we used MeRIP-qRT-PCR to detect changes in KRT8 mRNA m6A methylation in SKOV3 cells after treatment with EZMLD. We found that KRT8 mRNA m6A methylation modification levels increased significantly in the 5% and 10% EZMLD-containing serum groups compared to the control group (Fig. 1H).

### Role of METTL3/14 in m6A methylation and anticancer effects of EZMLD

**Fig. 2:**
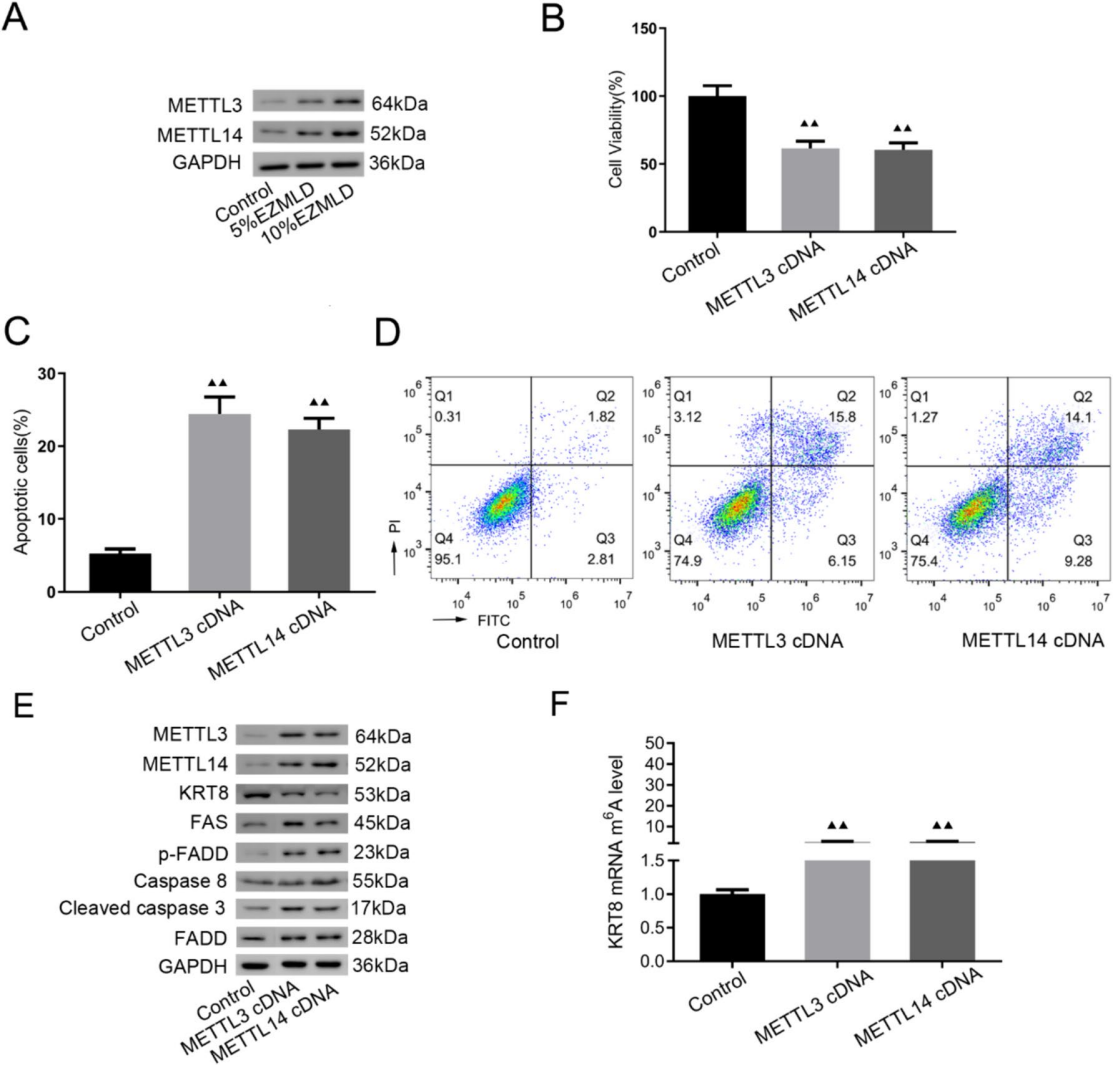
Exploration of role of METTL3/14 in SKOV3 cells. **A** The protein expression of METTL3 and METTL14 in SKOV3 cells after EZMLD treatment. **B** Induction of METTL3/14 cDNA in SKOV3 cells suppressed cell viability as determined by CCK8. **C**, **D** Flow cytometry analysis of apoptotic cells and quantification. **E** Western blot analysis of METTL3/14 and apoptosis-associated proteins. **F** Change in KTR8 mRNA m6A levels after induction of METTL3/14 cDNA in SKOV3 cells. Data are means of three independent experiments ± SD. ^▴▴^Compared to the control group, *P* < 0.01

We used Western blot to detect changes in related proteins to determine which enzymes are involved in modulating KRT8 m6A methylation. Unlike the blank serum group, the protein expression levels of METTL3 and METTL14 in SKOV3 cells treated with 5% and 10% EZMLD-containing serum demonstrated a significant increase (Fig. 2A). To determine the role of METTL3/14, we transfected METTL3/14 cDNA into SKOV3 cells and found that up-regulation of METTL3/14 significantly suppressed cell viability and increased cell apoptosis (Fig. 2B–D). Overexpression of METTL3/14 downregulated the KRT8 protein expression level and increased the expression levels of FAS, p-FADD, caspase-8, and cleaved caspase-3 proteins (Fig. 2E). Furthermore, the m6A methylation level of KRT8 increased significantly after overexpressing METTL3/14 (Fig. 2F). These results indicate that METTL3/14 plays a crucial role in modulating KRT8 m6A methylation, subsequently affecting KRT8 protein expression, cell viability, and apoptosis in SKOV3 cells.

**Fig. 3:**
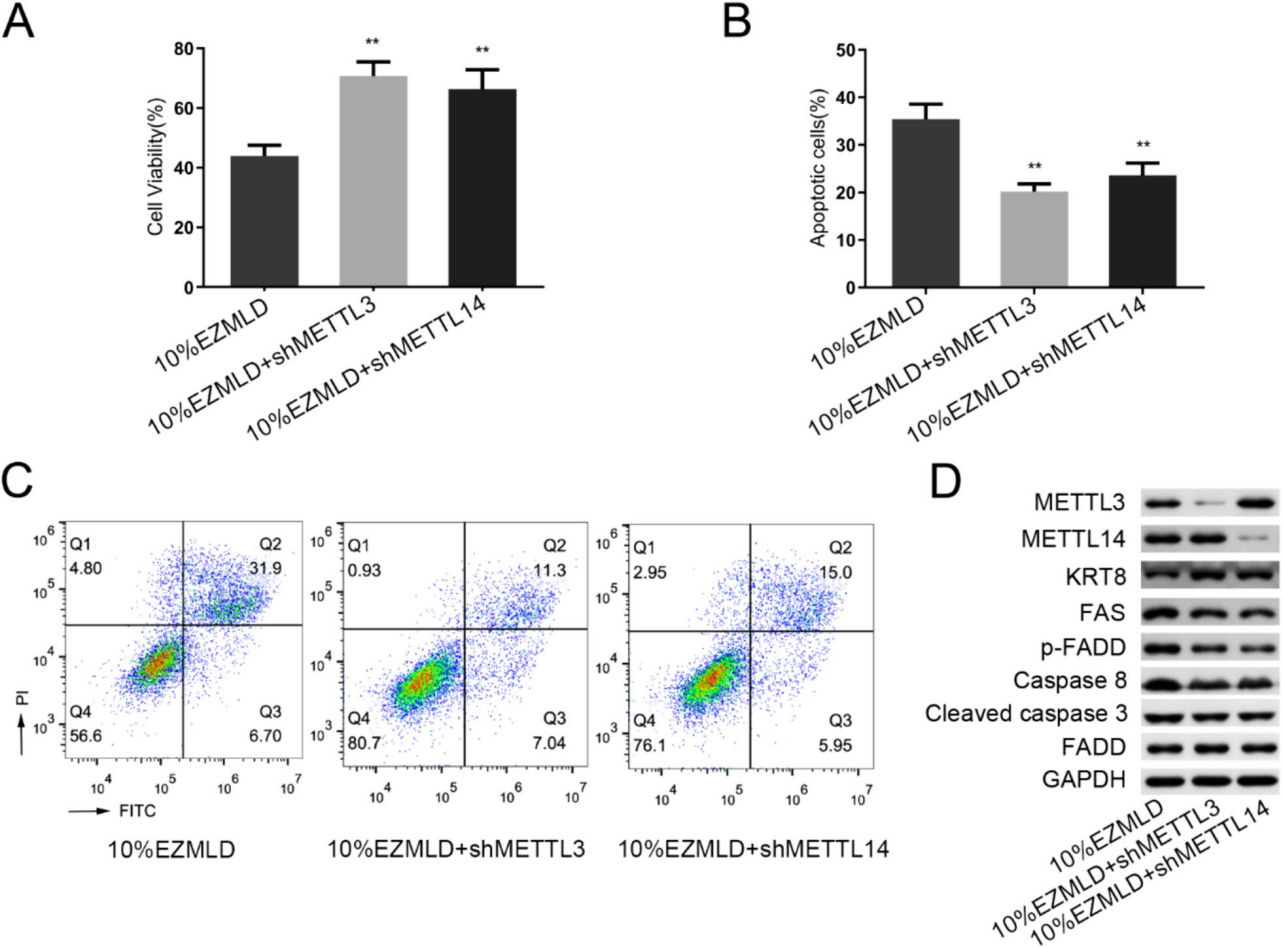
METTL3/14 knockdown could counteract the anticancer activities of EZMLD in SKOV3 cells. **A** METTL3/14 knockdown enhanced the cell viability of SKOV3 cells after EZMLD treatment. **B**, **C** METTL3/14 down-regulation reduced EZMLD-induced cell apoptosis. **D** METTL3/14 knockdown rescued the expression of apoptosis-associated proteins in EZMLD-treated SKOV3 cells. Data are means of three independent experiments ± SD. ^**^Compared to 10% EZMLD, *P* < 0.01

To explore whether METTL3/14 was involved in EZMLD-induced anticancer effects, we induced METTL3/14 knockdown by shRNA in SKOV3 cells and then subjected these cells to EZMLD treatment. METTL3/14 knockdown mitigated EZMLD’s anticancer activities in SKOV3 cells. Through the CCK8 assay, we discovered that METTL3/14 knockdown significantly improved the cell viability of SKOV3 cells after treatment with EZMLD (Fig. 3A). Furthermore, the apoptosis of these cells was also significantly reduced (Fig. 3B, C). METTL3/14 knockdown could counteract the up-regulation of METTL3/14 induced by EZMLD in SKOV3 cells (Fig. 3D). EZMLD-induced elevation of proteins such as FAS, p-FADD, Caspase-8, and cleaved Caspase-3 was reversed by METTL3/14 knockdown (Fig. 3D). The expression of KRT8 was significantly upregulated after transfection with METTL3/14 knockdown after exposure to EZMLD (Fig. 3D). These data suggest that METTL3/14 plays a vital role in the anticancer effect of EZMLD.

### In vivo effects of EZMLD on SKOV3 xenografts in nude mice

**Fig. 4:**
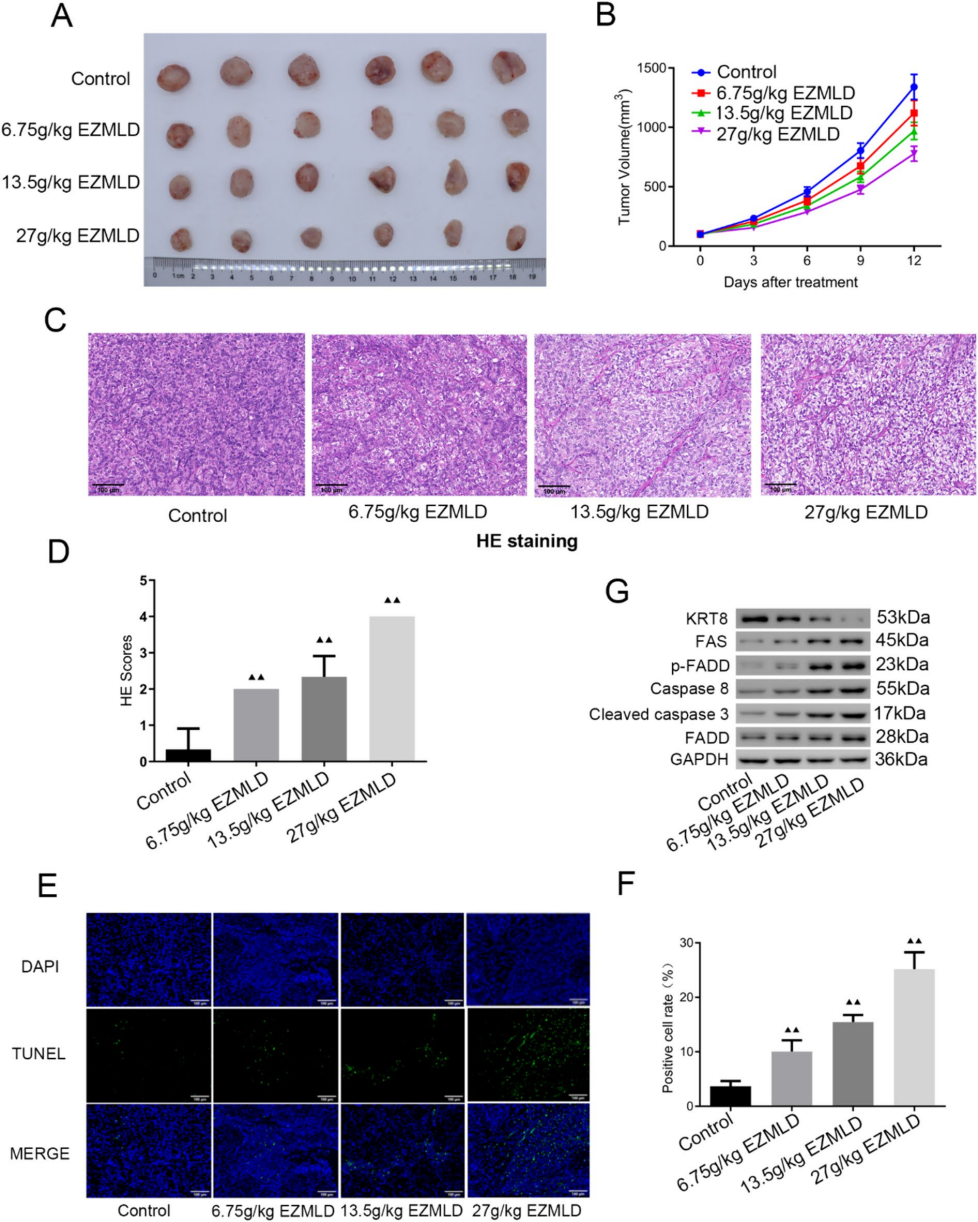
In vivo effects of EZMLD on SKOV3 xenografts in nude mice. **A** Representative images of nude mice bearing tumors formed by SKOV3 cells and administered with saline and different concentrations of EZMLD (0, 6.75, 13.5, 27 g/kg). Treatments were administered once daily by gavage for two consecutive weeks. **B** Tumor volumes after EZMLD treatment. **C**, **D** HE staining showed that EZMLD aggravated tumor tissue damage. **E**, **F** The TUNEL assay showed that EZMLD increased the percentage of apoptotic cells in tumor tissues of SKOV3 cell nude mouse xenografts. **G** Western blot analysis of apoptosis-associated proteins in SKOV3 cell nude mouse xenografts after EZMLD treatment. Data are means of three independent experiments ± SD. ^▴▴^Compared to the control group, *P* < 0.01

To elucidate the effects of EZMLD in vivo, we established nude mouse subcutaneous xenograft models using SKOV3 cells treated with different dosages of EZMLD (0, 6.75, 13.5, 27 g/kg). The results showed that EZMLD inhibited the growth of subcutaneous xenograft tumors in nude mice (Fig. 4A, B; Table 1). Furthermore, HE staining suggested that EZMLD aggravated tumor tissue damage (Fig. 4C, D). TUNEL staining was used to detect apoptosis in tumor tissues of each group of mice. The results indicated that, compared to the SKOV3 cell nude mouse xenografts treated with saline, there was a significant increase in the percentage of apoptotic cells in the tumor tissues of SKOV3 cell nude mouse xenografts treated with different concentrations of EZMLD (Fig. 4E, F). Western blot results showed that after treatment with EZMLD, the expression level of KRT8 protein in tumor tissues of nude mice decreased significantly. And the expression of the FAS, p-FADD, Caspase-8, and cleaved Caspase-3 proteins increased markedly, while the FADD protein showed only minor changes (Fig. 4G).

### M6A methylation and the antitumor mechanism of EZMLD in vivo

**Fig. 5:**
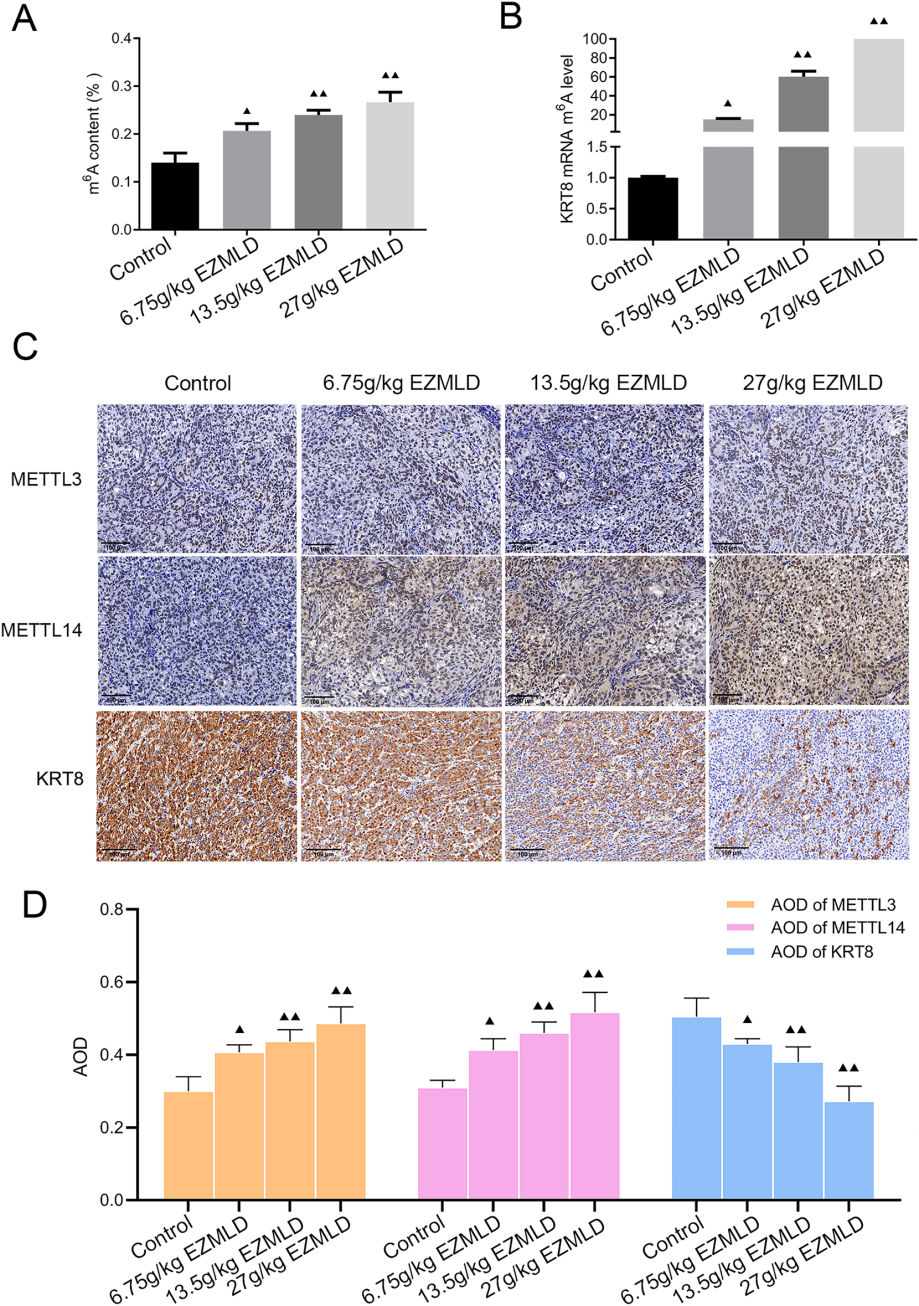
Exploration of the role of m6A methylation in EZMLD treatment in vivo*.* **A** Global m6A methylation quantification assay of m6A content in xenograft tumors of SKOV3 cells in nude mice after EZMLD treatment. **B** MeRIP-qRT-PCR of m6A methylation changes in KRT8 mRNA after EZMLD treatment in vivo. **C**, **D** Immunohistochemical assay showed that the expression of METTL3/14 proteins increased and the KRT8 protein decreased after EZMLD treatment in vivo. AOD, average optical density. Data are means of three independent experiments ± SD. Compared to the control group, ^▴^ *P* < 0.05, ^▴▴^ *P* < 0.01

To further validate the role of m6A methylation regulation in the antitumor effects of EZMLD in vivo, a methylation quantification experiment was conducted to measure the m6A levels in SKOV3 cell xenograft tumors in nude mice. Compared to SKOV3 xenografts treated with saline in nude mice, m6A content increased significantly in tumors treated with different concentrations of EZMLD (Fig. 5A). MeRIP-qRT-PCR was used to assess changes in m6A methylation in KRT8 mRNA of various xenografts. Compared to SKOV3 xenografts treated with saline, those treated with EZMLD showed significantly elevated levels of KRT8 mRNA methylation (Fig. 5B).

Immunohistochemical results revealed that EZMLD treatment significantly increased METTL3 and METTL14 protein expression and decreased KRT8 expression in xenografted tumors in nude mice (Fig. 5C, D).

**Fig. 6:**
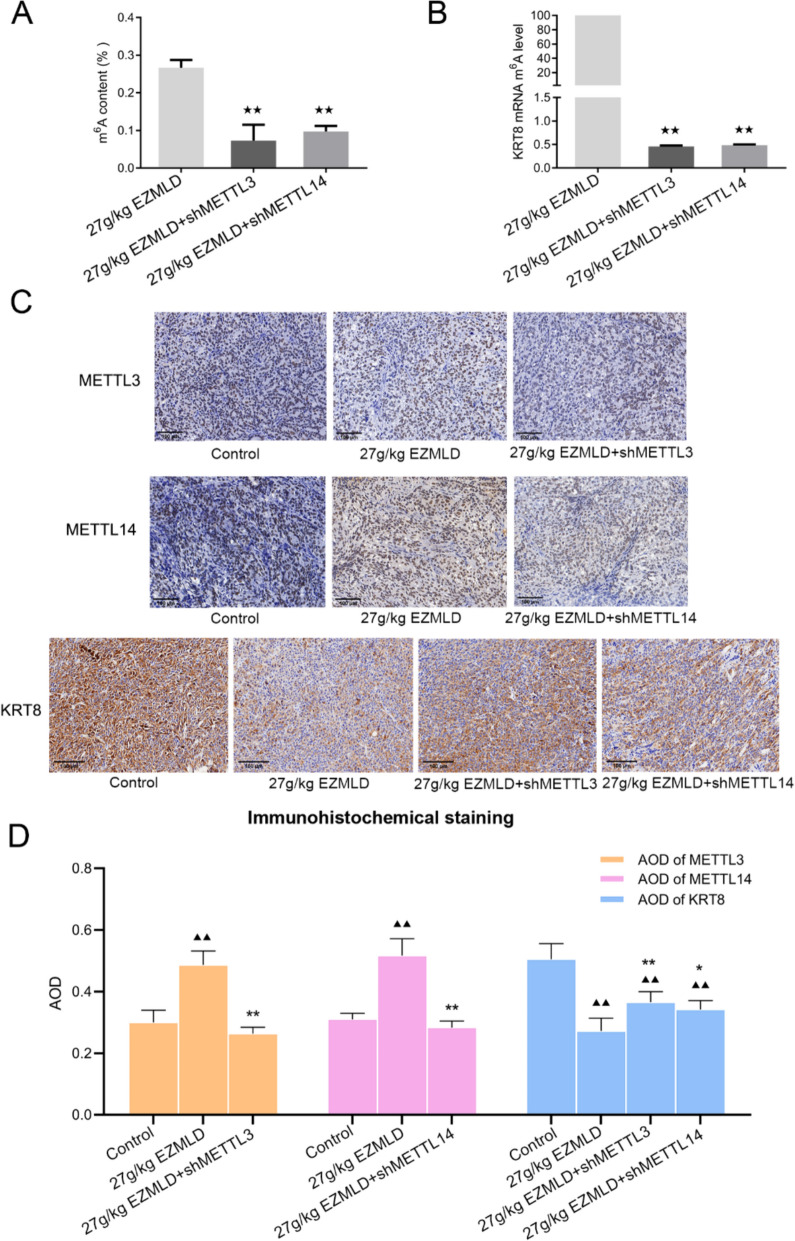
Exploration of underlying regulatory mechanism of METTL3/14 in EZMLD treatment in vivo. **A** Global m6A methylation quantification assay of the m6A percentage content in SKOV3 cell xenografts with downregulation of METTL3/14 and 27 g/kg of EZMLD treatment. **B** MeRIP-qRT-PCR of m6A methylation changes in KRT8 mRNA in SKOV3 cell xenografts with down-regulation of METTL3/14 and 27 g/kg EZMLD treatment. **C**, **D** METTL3/14 knockdown counteracted upregulation of METTL3/14 and downregulation of KRT8 induced by EZMLD detected by immunohistochemical assay. Compared to the control group, ^▴▴^ *P* < 0.01. Compared to SKOV3 cells xenografts treated with 27 g/kg EZMLD, ^*^ *P* < 0.05, ^**^ *P* < 0.01

**Fig. 7:**
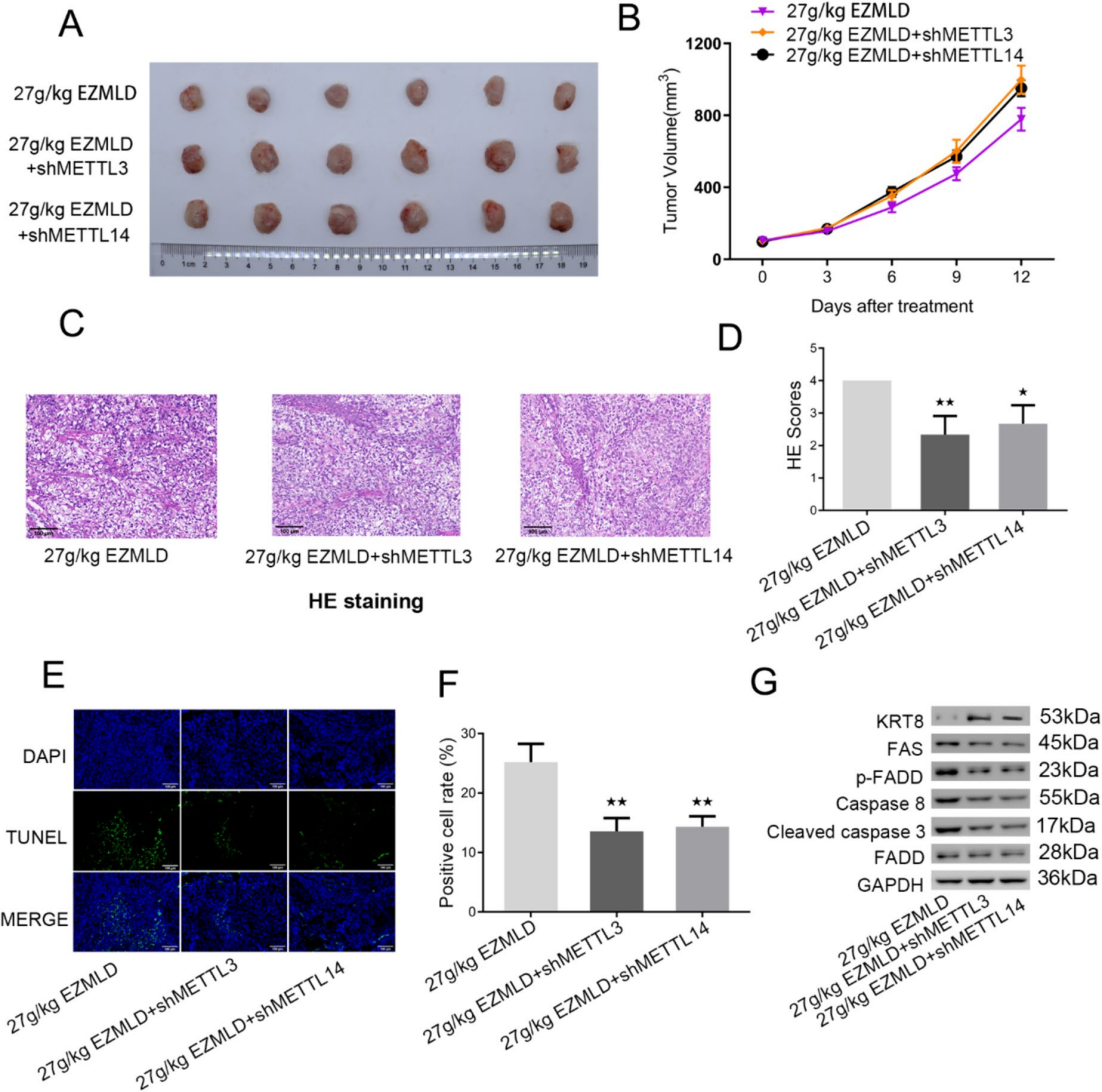
METTL3/14 knockdown mitigated EZMLD’s antitumor effects and modulated apoptosis-related protein expression in vivo. **A**, **B** Representative images and tumor volumes in nude mice with subcutaneous injection of SKOV3 cells and SKOV3 cells with METTL3/14 knockdown and then treated with EZMLD. **C**, **D** HE staining showed alleviation of tumor tissue damage in xenografts with down-regulation of METTL3/14 after 27 g/kg EZMLD treatment. **E**, **F** TUNEL assay showing a reduction in the apoptosis rate of positive cells in tumor tissues in xenografts with down-regulation of METTL3/14 after 27 g/kg of EZMLD treatment. **G** Western blot analysis of apoptosis-associated proteins in SKOV3 cells and cells with METTL3/14 knockdown nude mouse xenografts and then treated with EZMLD. Compared to SKOV3 cell xenografts treated with 27 g/kg of EZMLD, ^*^ *P* < 0.05, ^**^ *P* < 0.01

To further explore the in vivo mechanism of METTL3/14 methyltransferases in EZMLD treatment, ovarian cancer xenografts were established in nude mice with SKOV3 cells with METTL3 and METTL14 knockdown. They were treated with 27 g/kg of EZMLD. After down-regulating METTL3/14, the m6A content in SKOV3 cell xenografts in nude mice decreased significantly (Fig. 6A), and the methylation level of KRT8 mRNA was significantly reduced (Fig. 6B). Furthermore, METTL3/14 knockdown could counteract the upregulation of METTL3/14 and the downregulation of KRT8 induced by EZMLD (Fig. 6C, D). Moreover, METTL3/14 knockdown could mitigate the antitumor effects of EZMLD in vivo. Compared to SKOV3 cell xenografts, xenografts with down-regulation of METTL3/14 showed an increase in tumor size after 27 g/kg of EZMLD treatment (Fig. 7A, B; Table 1). HE staining showed alleviated tumor tissue damage, and cells were more tightly packed (Fig. 7C, D). The results of the TUNEL assay indicated a significant reduction in the rate of positive cell apoptosis in tumor tissues (Fig. 7E, F). Concurrently, Western blot analysis demonstrated increased KRT8 protein expression, while the expression of proteins such as FAS, p-FADD, Caspase-8, and cleaved Caspase-3 decreased (Fig. 7G). These results demonstrate that EZMLD acts primarily by modulating the expression of METTL3/14, which in turn influences the expression of KRT8 and then FAS-mediated apoptotic proteins.

## Discussion

This study elucidated the antitumor effects of EZMLD on ovarian cancer cells. Data provide a comprehensive understanding of the cytotoxic mechanisms of EZMLD at the genetic level, revealing potential biomarkers that could guide future research and therapeutic approaches for ovarian cancer. Although notable advances have been made in the detection and treatment of ovarian cancer in recent years, the mortality rate remains high. Drug resistance is one of the main causes of treatment failure and patient death [19]. In recent years, TCM and its primary components have been shown to have antitumor effects in various cancers. According to TCM theories, Mo Han Lian and Nv Zhen Zi are the primary ingredients in EZMLD, also called monarch drugs in TCM, and can nourish and balance kidneys. Bai Zhu and Bai Shao act as auxiliary ingredients, called minister drugs, which can strengthen the spleen, aid digestion, soothe the liver, and nourish the blood. These four herbs replenish congenital *Yin* and nourish the postnatal foundation. Mao Ren Shen and Zhe Bei Mu are also auxiliary drugs. Gui Zhi can warm the spleen's *yang* energy to dispel dampness and facilitate the circulation of the meridians to expel water and regulate *qi*. Fu Ling strengthens the spleen and purifies impurities, serving as an adjuvant ingredient. All these herbs cooperate and achieve the effect of reducing masses and dissipating nodules. In previous studies, the monarch drugs Mo Han Lian and Nv Zhen Zi have been reported to exhibit anticancer effects against hepatocellular carcinoma [20–22]. Nv Zhen Zi extracts have also been reported to induce human glioma cell death [23], and enhance the sensitivity of human colorectal carcinoma cells to doxorubicin-induced apoptosis [24].

Numerous active ingredients in the components of the decoction have also demonstrated anticancer properties in various tumors. For example, atractylenolide II, isolated from Bai Zhu, has been reported to inhibit cell proliferation and induce apoptosis of gastric carcinoma and melanoma cells [25, 26]. In our study, we found that EZMLD could inhibit the cell viability of SKOV3 ovarian cancer cells by inducing apoptosis and triggering the Fas-mediated extrinsic apoptotic pathway. Fas is a death receptor that belongs to the tumor necrosis factor receptor gene superfamily. By binding to the Fas ligand, Fas induces the recruitment of the adaptor molecule such as FADD and caspase-8. Activated caspase-8 then initiates apoptosis by direct cleaving downstream effector caspases such as caspase-3, thereby leading to a controlled cell death process [27].

Growing evidence indicates that the development of ovarian cancer results from the interaction of genetics, epigenetics, and transcriptomics [28, 29]. In recent times, many studies have highlighted a link between m6A and the occurrence and progression of ovarian cancer [30]. In this study, we observed hypomethylation of m6A in KRT8 mRNA from ovarian cancer tissues, posttreatment with EZMLD led to a significant increase in m6A content in vivo and elevated m6A methylation of KRT8 mRNA in both in vitro and in vivo. We also observed that after EZMLD treatment, the expression of the KRT8 protein decreased. This indicates that EZMLD may act by modulating the m6A methylation status of the KRT8 mRNA, thus affecting its stability and translation efficiency.

Whether METTL3 and METTL14 play a tumorigenic or tumor-suppressor role in cancer remains controversial and inconsistent across various studies. For instance, METTL3 may play a tumor-suppressor role in renal cell carcinoma's cell proliferation, migration, invasion, and cell cycle functions [31]. However, it could also promote the tumorigenesis of human renal cell carcinoma by mediating HHLA2 mRNA m6A modification [32]. Similarly, elevated METTL3 suppresses colorectal cancer proliferation and migration [33], while METTL3 promotes colorectal cancer tumorigenesis and metastasis [34, 35]. METTL14 has been reported to exert contrasting effects, promoting the growth and metastasis of pancreatic cancers [36] and breast cancers [37]. In contrast, it inhibits the proliferation and metastasis of gastric cancers [38, 39] and colorectal cancers [40]. Similarly, although most literature supports that METTL3/14 promotes ovarian carcinogenesis [41, 42], there are reports suggesting that METTL3/14 inhibits ovarian carcinogenesis. For example, ovarian cancer cell growth was enhanced in METTL3-cKO mice [43]; while METTL14 overexpression decreased ovarian cancer proliferation by inhibition of TROAP expression via an m6A RNA methylation-dependent mechanism [44].These contradictions may be due to the complex functions of the METTL3/14 genes and the heterogeneity of the tumor. In our study, we found that the expression level of METTL3/14 was up-regulated in SKOV3 cells after EZMLD treatment. Additionally, overexpression of METT3/14 induced anticancer effects similar to those of EZMLD, while METTL3/14 knockdown mitigated the anticancer effects of EZMLD. Taken together, these data indicated that EZMLD may function by modulating METTL3/14. Combined with alterations in the expression of the KRT8 protein and downstream proteins involved in the FAS-mediated apoptosis pathway, our results suggest that EZMLD exerts its effects by modulating the METTL3/14-KRT8-FAS signal cascade. In vivo studies on nude mouse xenografts further provided strong evidence of the therapeutic potential of EZMLD. Concentration-dependent inhibition of tumor growth and increased apoptosis further validate in vitro observations. Additionally, alterations in protein expression levels within tumor tissues corroborate the molecular pathways identified in cell cultures.

Although this study has revealed significant findings, there are several limitations of the research. First, our focus was primarily on cell apoptosis and related pathways induced by EZMLD, leaving other effects and a more in-depth exploration of potential pathways influenced by EZMLD for future investigations. Second, our focus was on the research of the formulation itself, and potential synergies with other therapeutic methods should have been discussed. Third, chemical components of the EZMLD decoction or drug-containing serum, and their correlation with METTL3 and METTL14, require further analysis. And finally, the toxicity profile of the decoction requires further exploration.

## Conclusions

Our study demonstrates that EZMLD exerts significant antitumor effects on SKOV3 ovarian cancer cells in vitro and in vivo by modulating METTL3/14 expression. This modulation alters the m6A methylation of KRT8 mRNA, which influences the levels of FAS apoptosis-related proteins, thus promoting cell apoptosis. These findings contribute to a deeper understanding of the molecular mechanisms underpinning EZMLD’s antitumor effects and establish a foundation for further exploration of its clinical applications.

### Supplementary Information

Supplementary Material 1.

## Data Availability

The datasets used and/or analyzed during the current study are available from the corresponding author on reasonable request.

## Abbreviations

EZMLD: Erzhimaoling decoction
TCM: Traditional Chinese medicine
PFS: Progression-free survival
m6A: *N*6-methyladenosine
shRNA: Short hairpin RNA
CCK-8: Cell Counting Kit-8
MeRIP-seq: Methylated RNA immunoprecipitation sequencing
HE: Hematoxylin and eosin
TUNEL: Terminal deoxynucleotidyl transferase d-UTP nick end labeling
AOD: Average optical density
SD: Standard deviation
KRT8: Keratin 8
MeRIP-qRT-PCR: Methylated RNA immunoprecipitation-quantitative reverse transcription PCR
ANOVA: Analysis of variance
p-FADD: Phospho-FADD (Ser194)
