# Supplementary figures and images for: Anticancer effects of Erzhimaoling decoction in high-grade serous ovarian cancer in vitro and in vivo
Source: Eur J Med Res. 2024 Aug 5;29:405. doi: 10.1186/s40001-024-01968-4 (PMC11299366; doi:10.1186/s40001-024-01968-4)

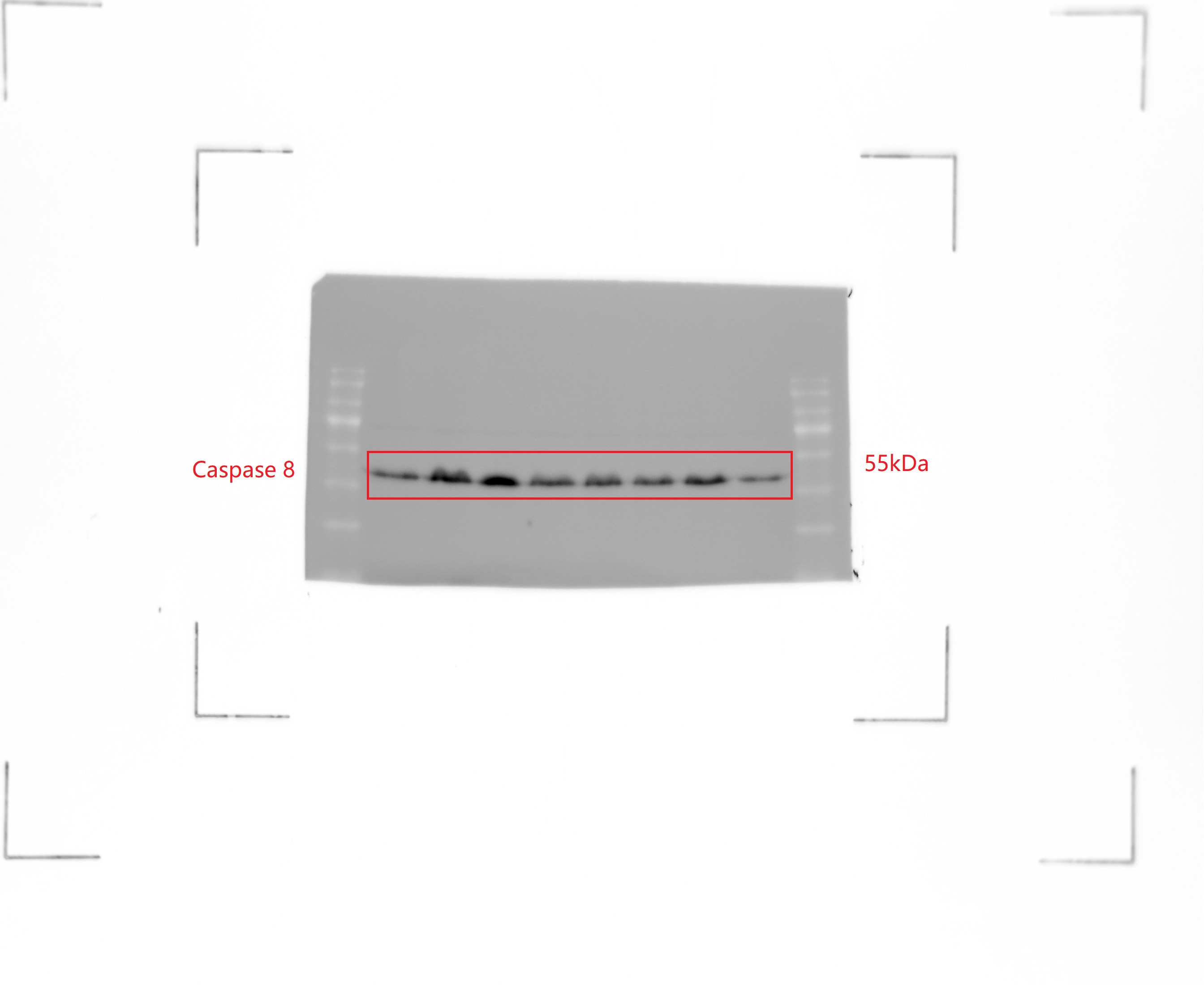

Supplement: Supplementary file 1 — Supplementary Material 1. [file 40001_2024_1968_MOESM1_ESM.zip › western blot original images/FIGURE1 original image/Caspase 8.jpg]

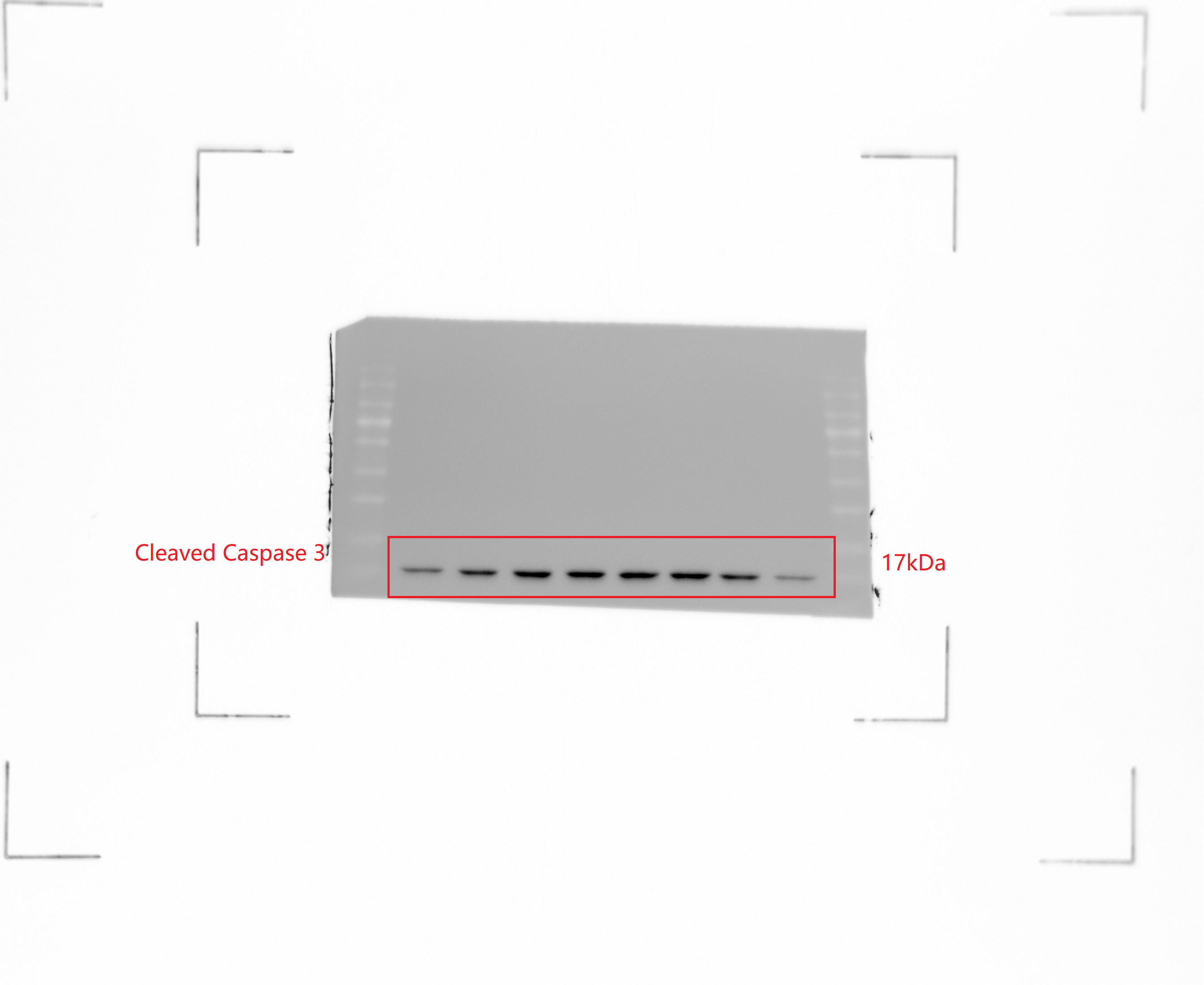

Supplement: Supplementary file 1 — Supplementary Material 1. [file 40001_2024_1968_MOESM1_ESM.zip › western blot original images/FIGURE1 original image/Cleaved Caspase 3.jpg]

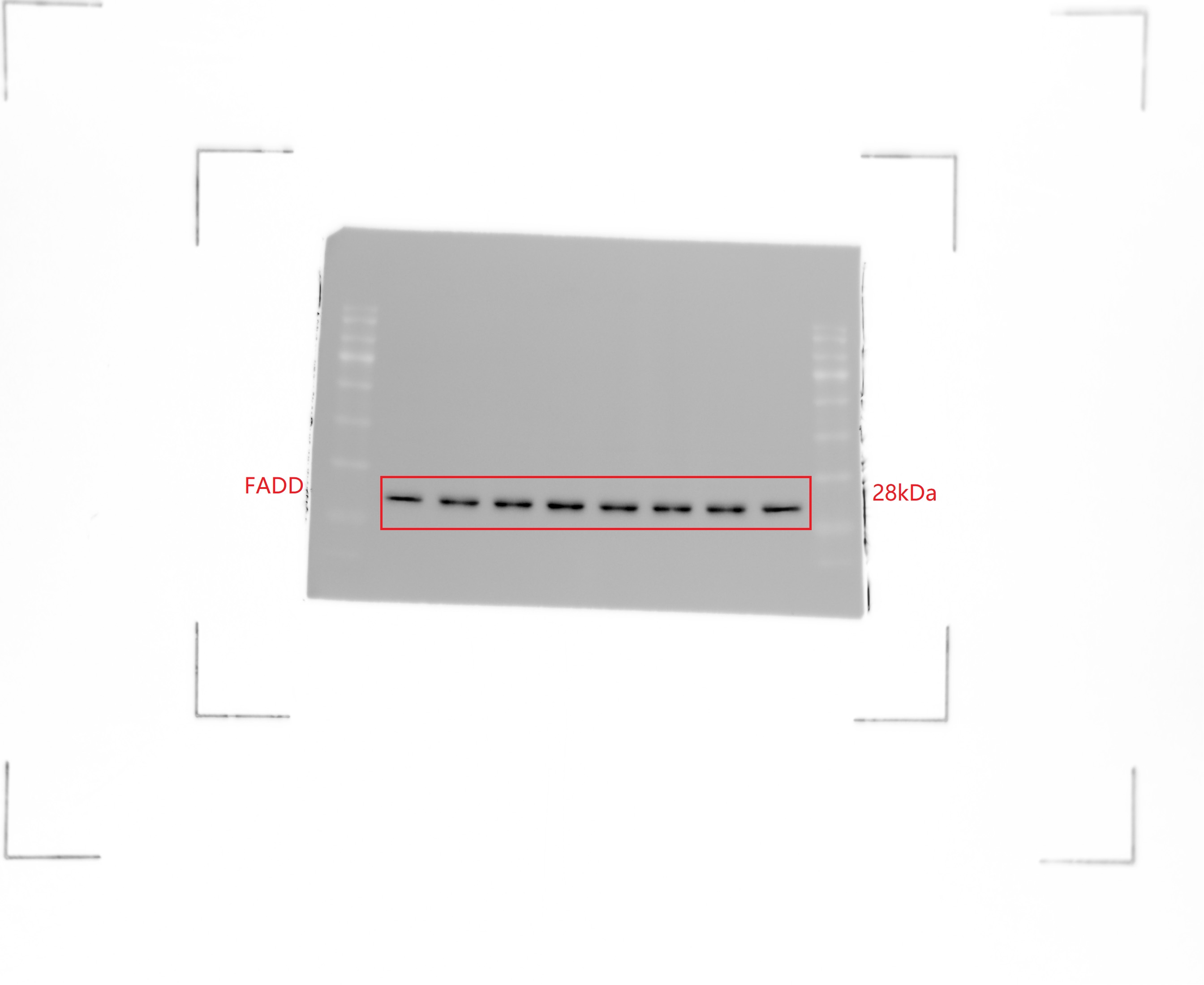

Supplement: Supplementary file 1 — Supplementary Material 1. [file 40001_2024_1968_MOESM1_ESM.zip › western blot original images/FIGURE1 original image/FADD.jpg]

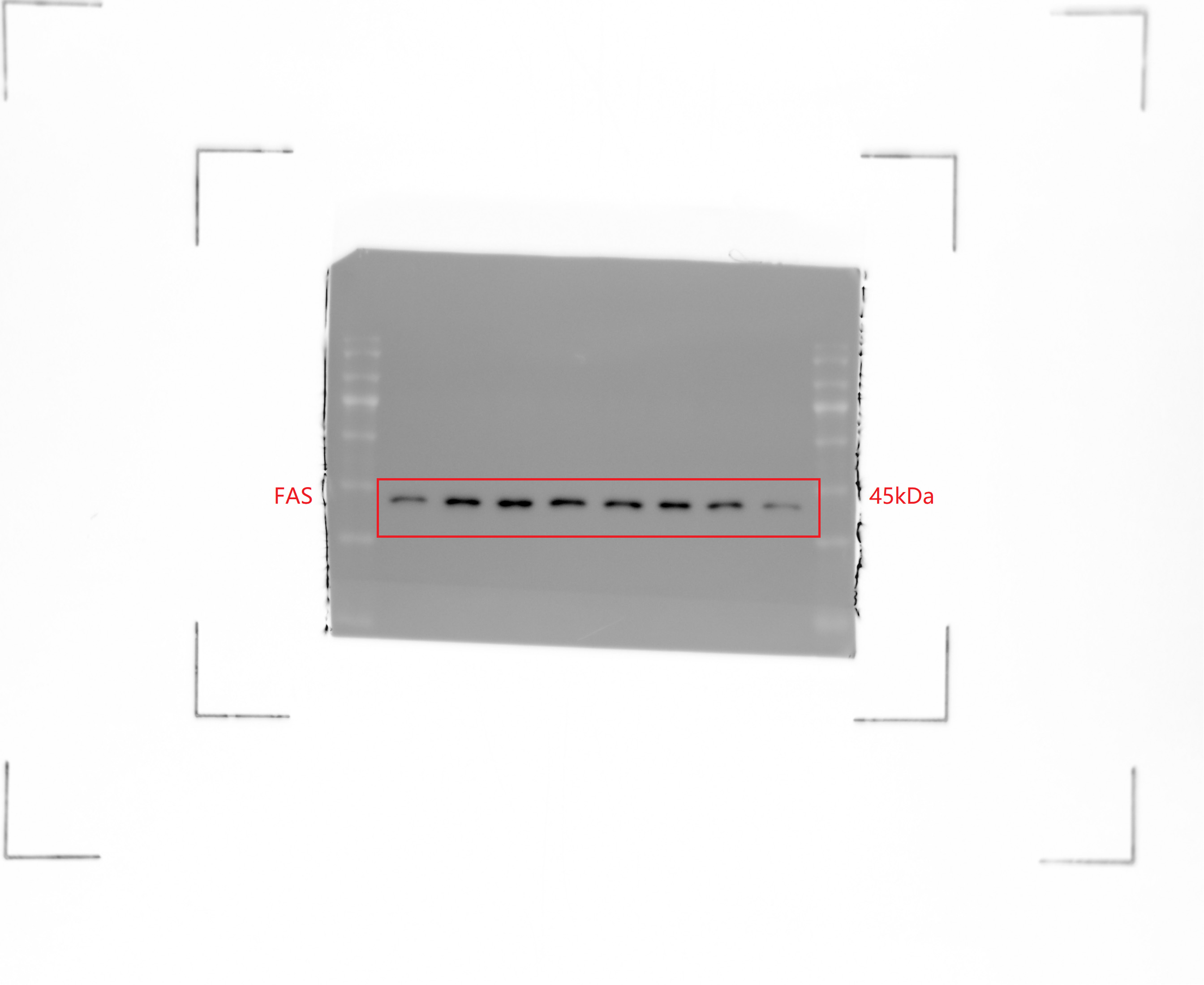

Supplement: Supplementary file 1 — Supplementary Material 1. [file 40001_2024_1968_MOESM1_ESM.zip › western blot original images/FIGURE1 original image/FAS.jpg]

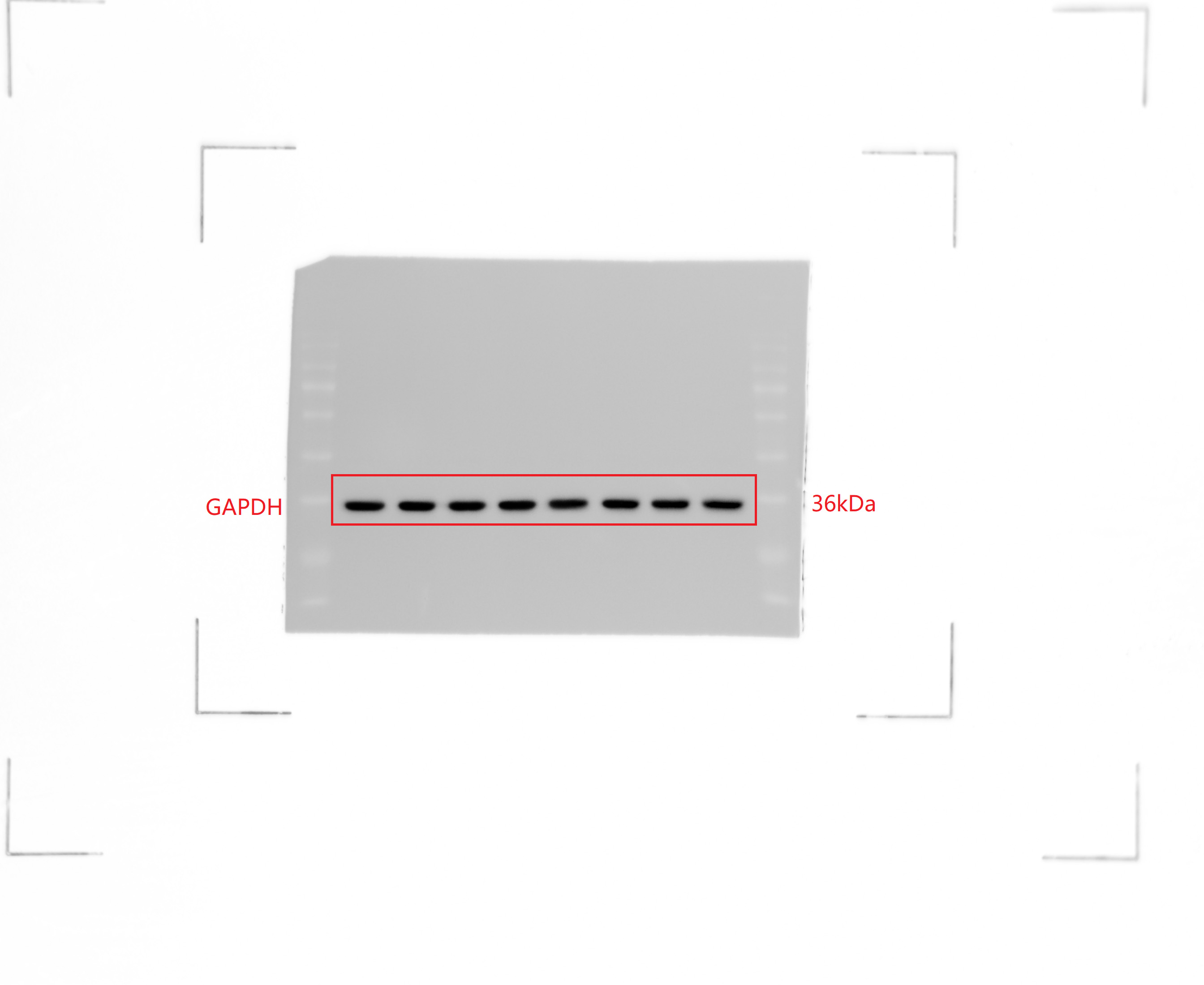

Supplement: Supplementary file 1 — Supplementary Material 1. [file 40001_2024_1968_MOESM1_ESM.zip › western blot original images/FIGURE1 original image/GAPDH.jpg]

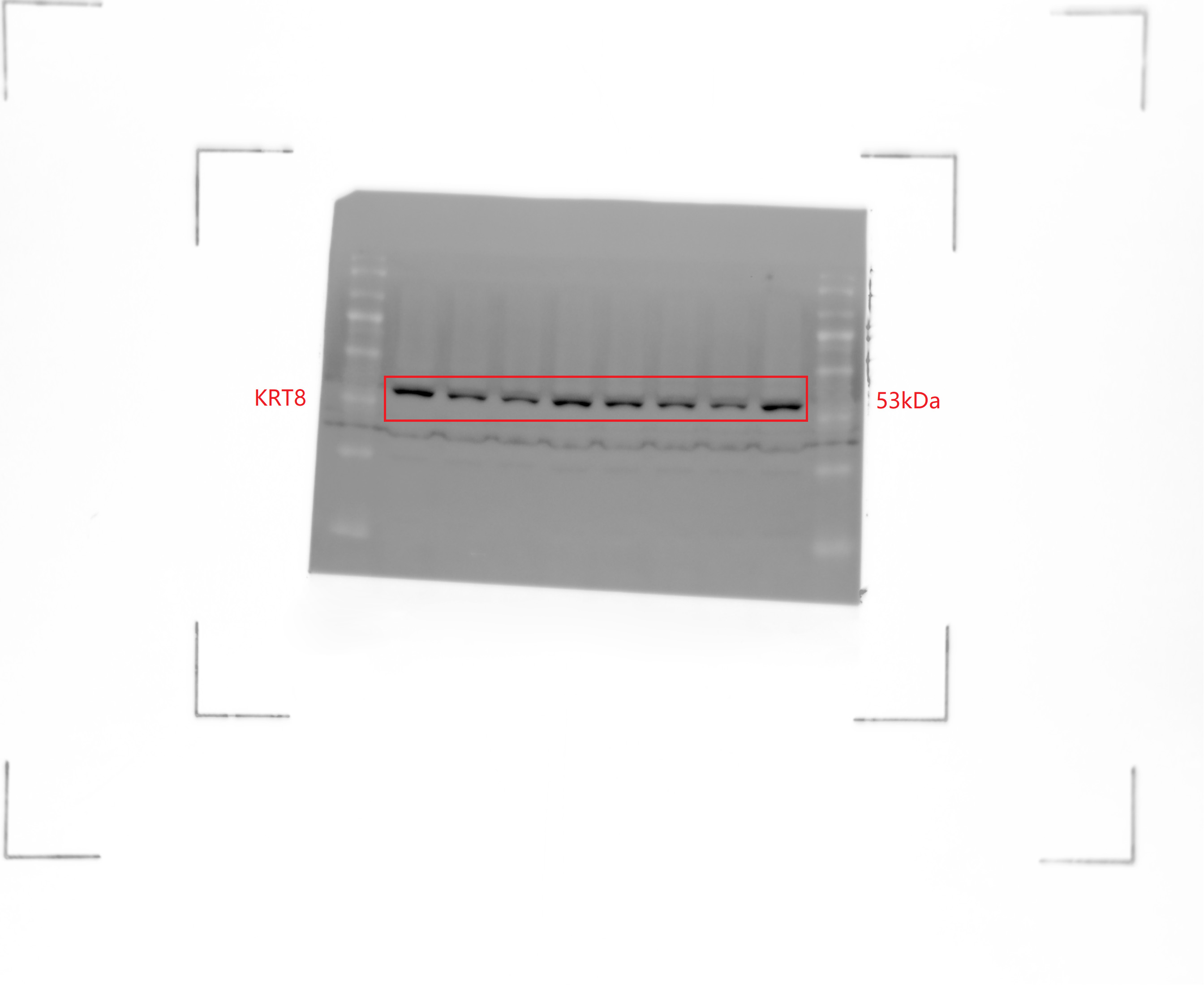

Supplement: Supplementary file 1 — Supplementary Material 1. [file 40001_2024_1968_MOESM1_ESM.zip › western blot original images/FIGURE1 original image/KRT8.jpg]

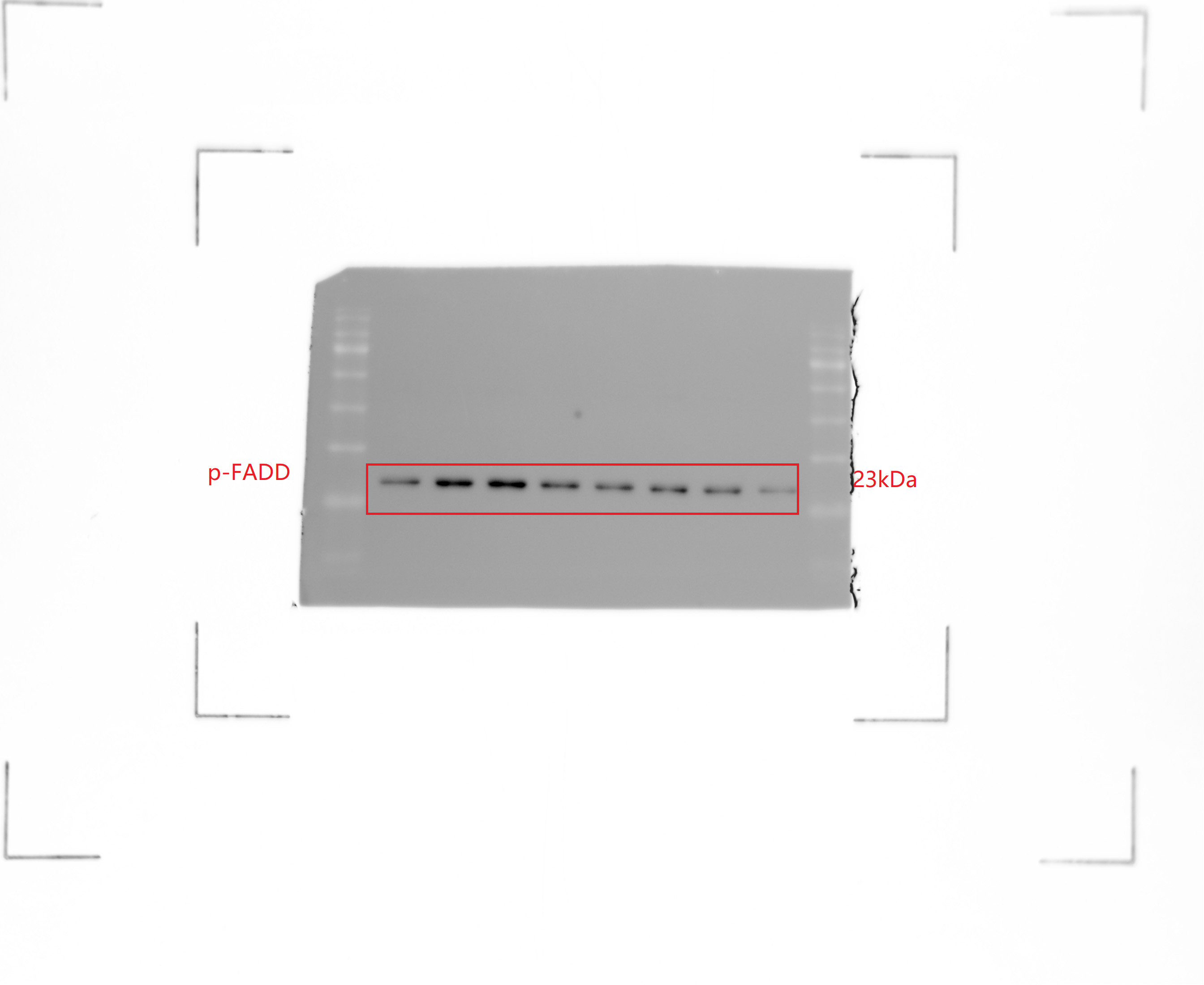

Supplement: Supplementary file 1 — Supplementary Material 1. [file 40001_2024_1968_MOESM1_ESM.zip › western blot original images/FIGURE1 original image/p-FADD.jpg]

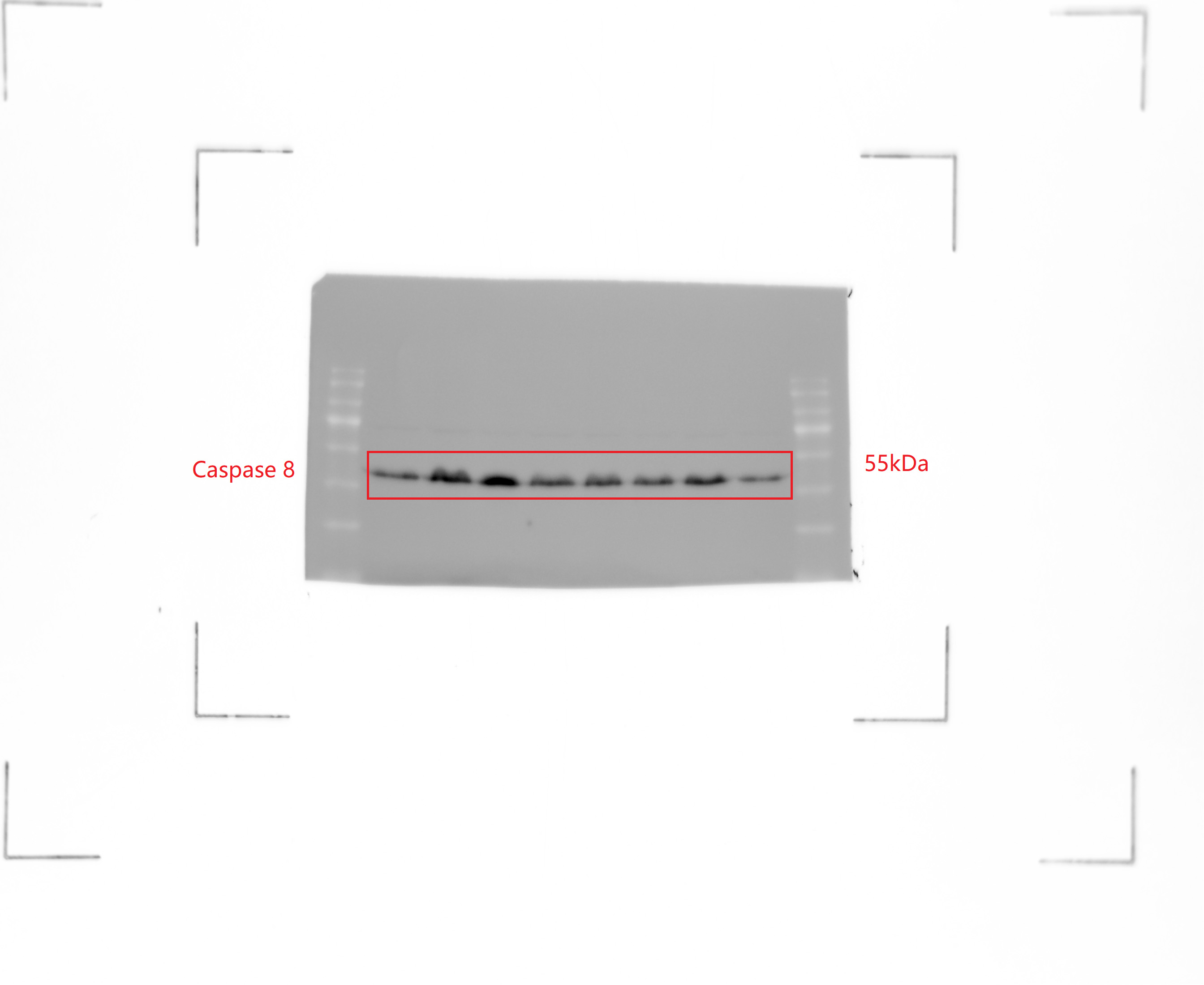

Supplement: Supplementary file 1 — Supplementary Material 1. [file 40001_2024_1968_MOESM1_ESM.zip › western blot original images/FIGURE3 original image/Caspase 8.jpg]

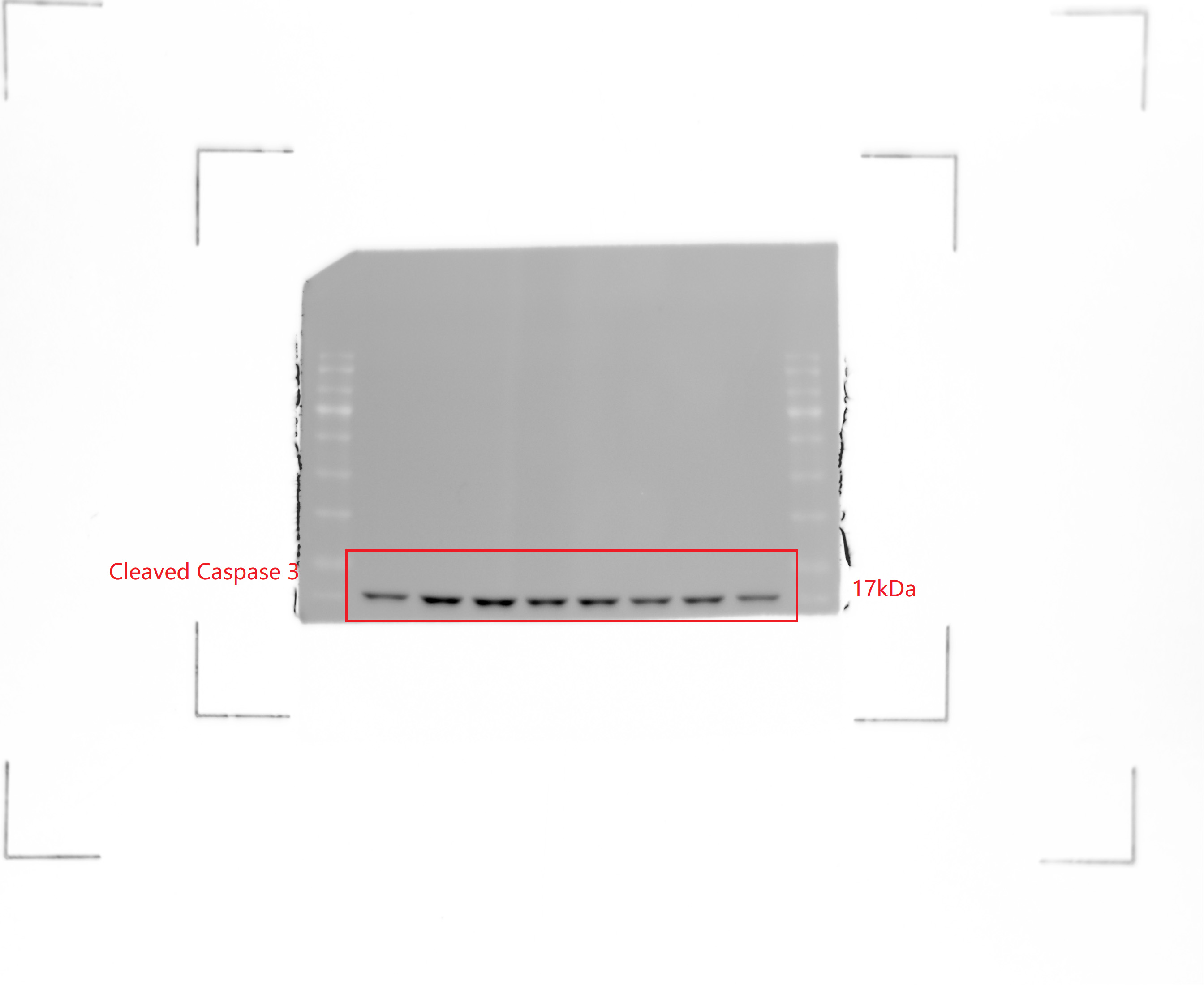

Supplement: Supplementary file 1 — Supplementary Material 1. [file 40001_2024_1968_MOESM1_ESM.zip › western blot original images/FIGURE3 original image/Cleaved Caspase 3.jpg]

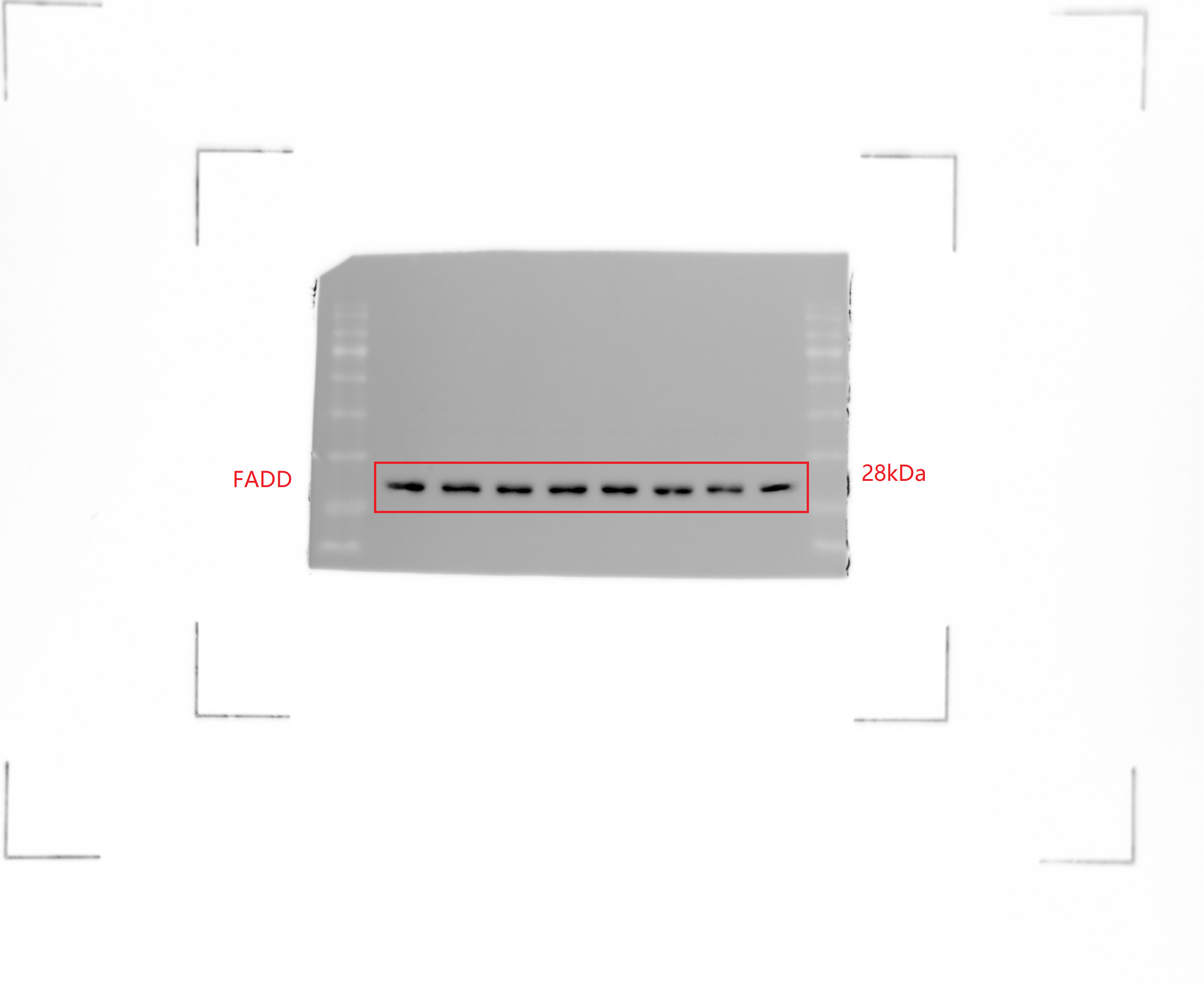

Supplement: Supplementary file 1 — Supplementary Material 1. [file 40001_2024_1968_MOESM1_ESM.zip › western blot original images/FIGURE3 original image/FADD.jpg]

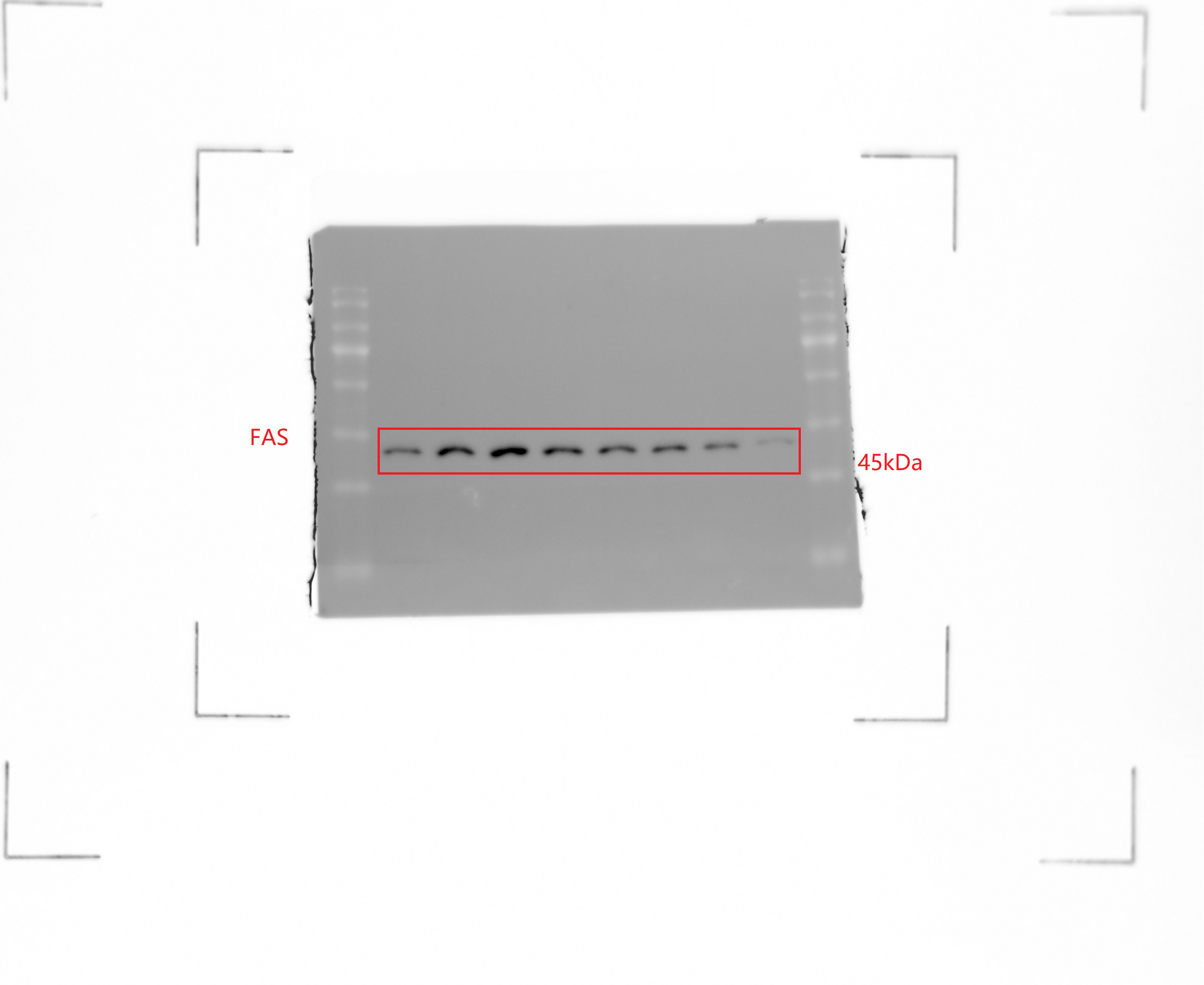

Supplement: Supplementary file 1 — Supplementary Material 1. [file 40001_2024_1968_MOESM1_ESM.zip › western blot original images/FIGURE3 original image/FAS.jpg]

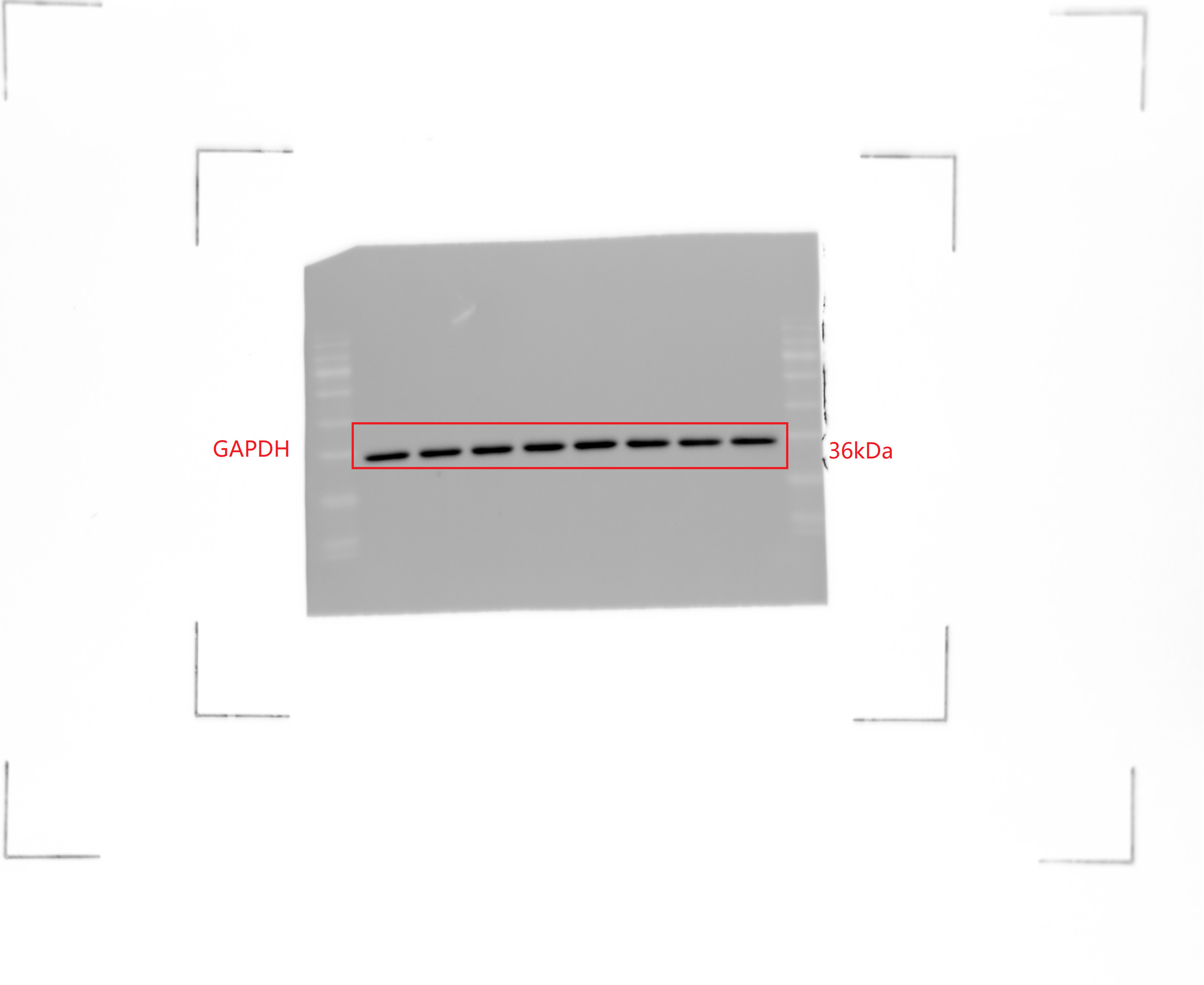

Supplement: Supplementary file 1 — Supplementary Material 1. [file 40001_2024_1968_MOESM1_ESM.zip › western blot original images/FIGURE3 original image/GAPDH.jpg]

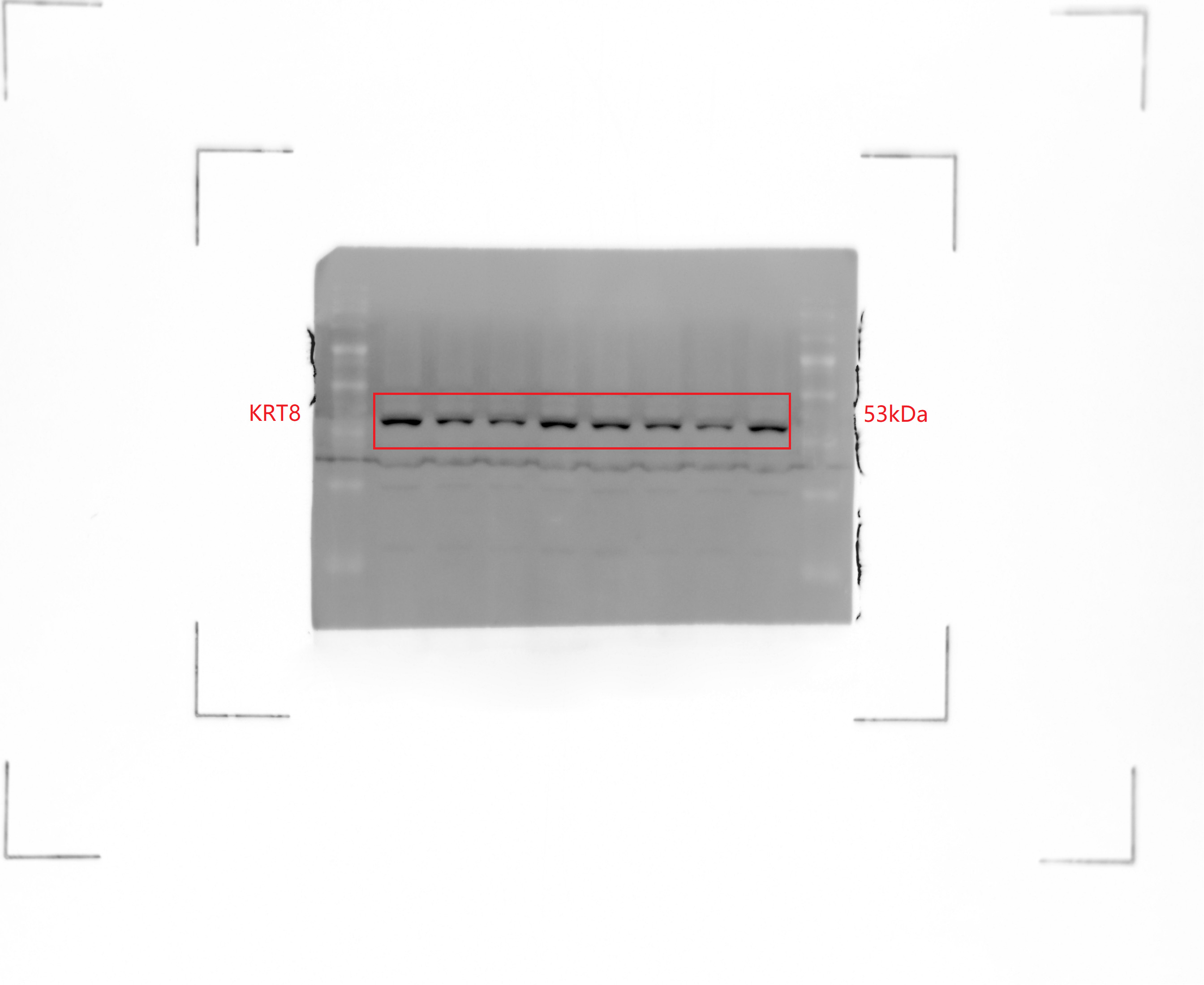

Supplement: Supplementary file 1 — Supplementary Material 1. [file 40001_2024_1968_MOESM1_ESM.zip › western blot original images/FIGURE3 original image/KRT8.jpg]

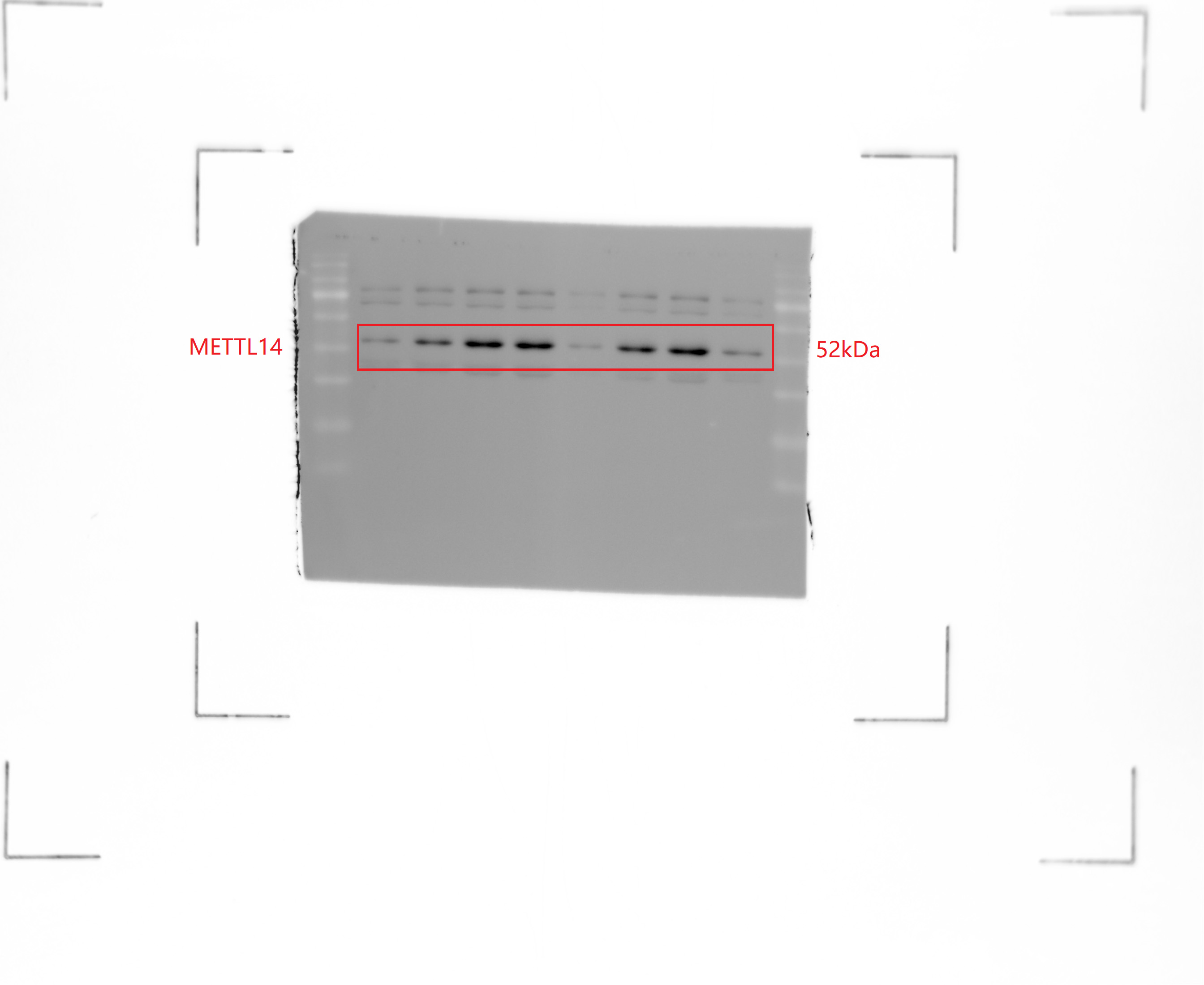

Supplement: Supplementary file 1 — Supplementary Material 1. [file 40001_2024_1968_MOESM1_ESM.zip › western blot original images/FIGURE3 original image/METTL14.jpg]

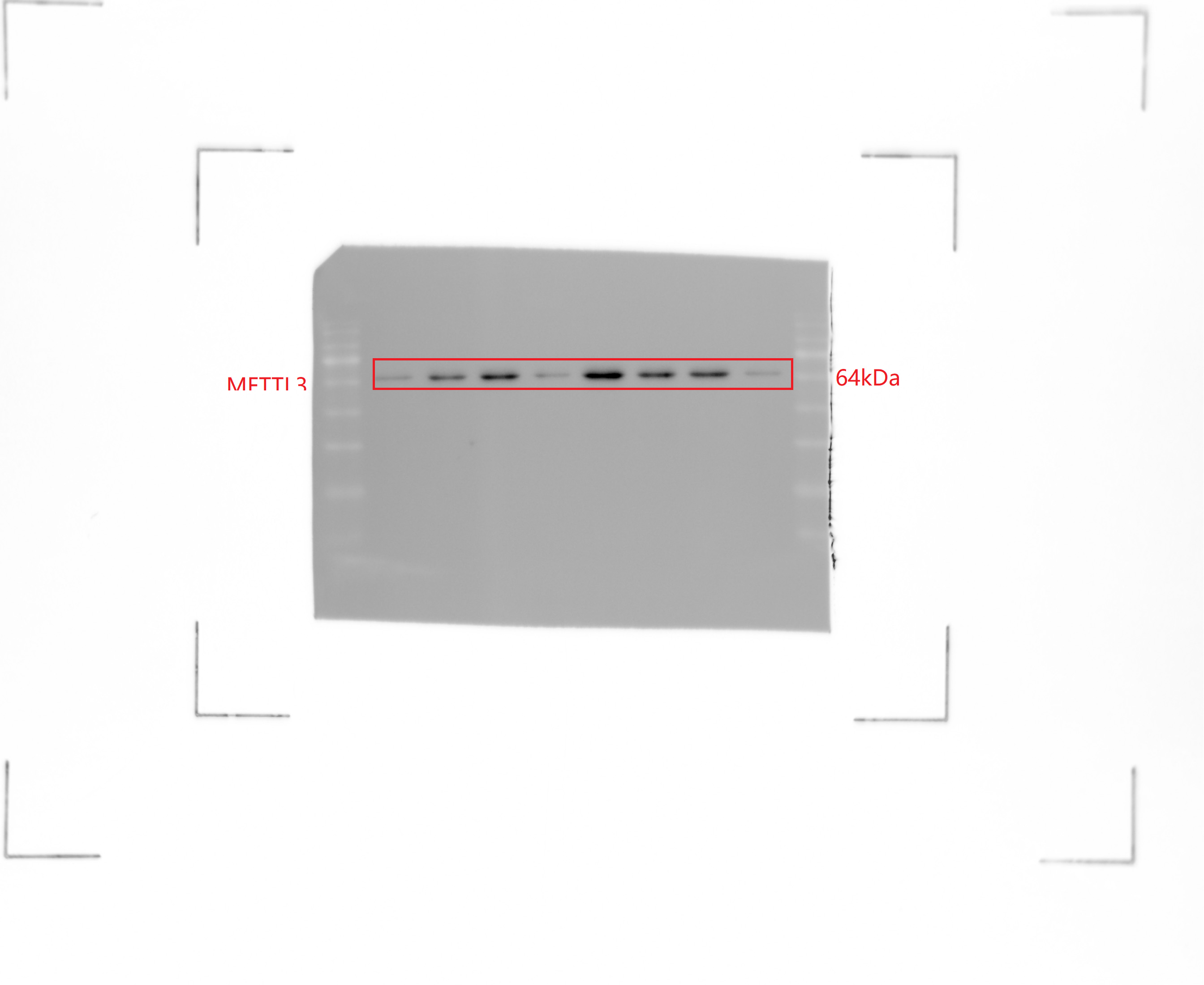

Supplement: Supplementary file 1 — Supplementary Material 1. [file 40001_2024_1968_MOESM1_ESM.zip › western blot original images/FIGURE3 original image/METTL3.jpg]

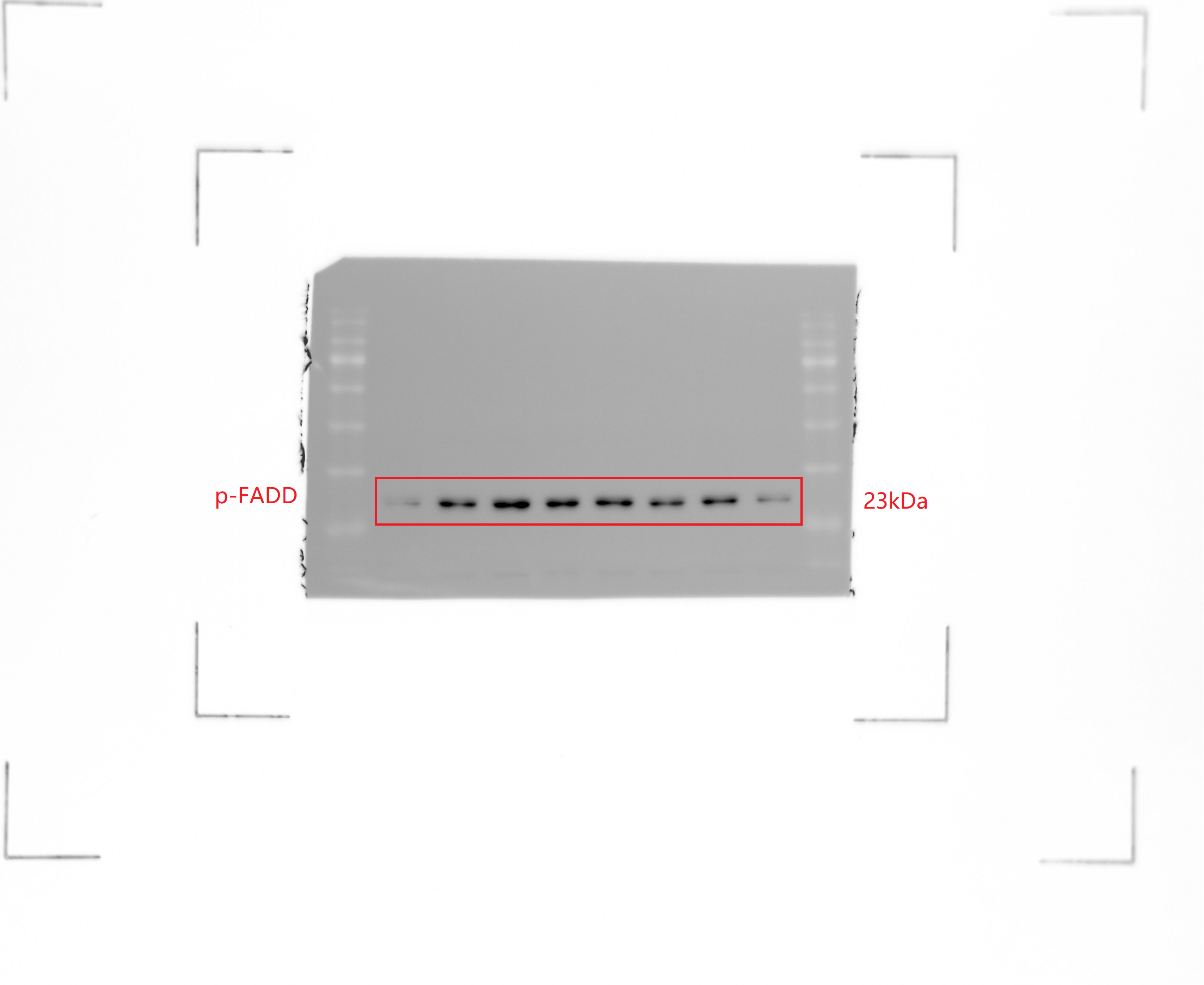

Supplement: Supplementary file 1 — Supplementary Material 1. [file 40001_2024_1968_MOESM1_ESM.zip › western blot original images/FIGURE3 original image/p-FADD.jpg]

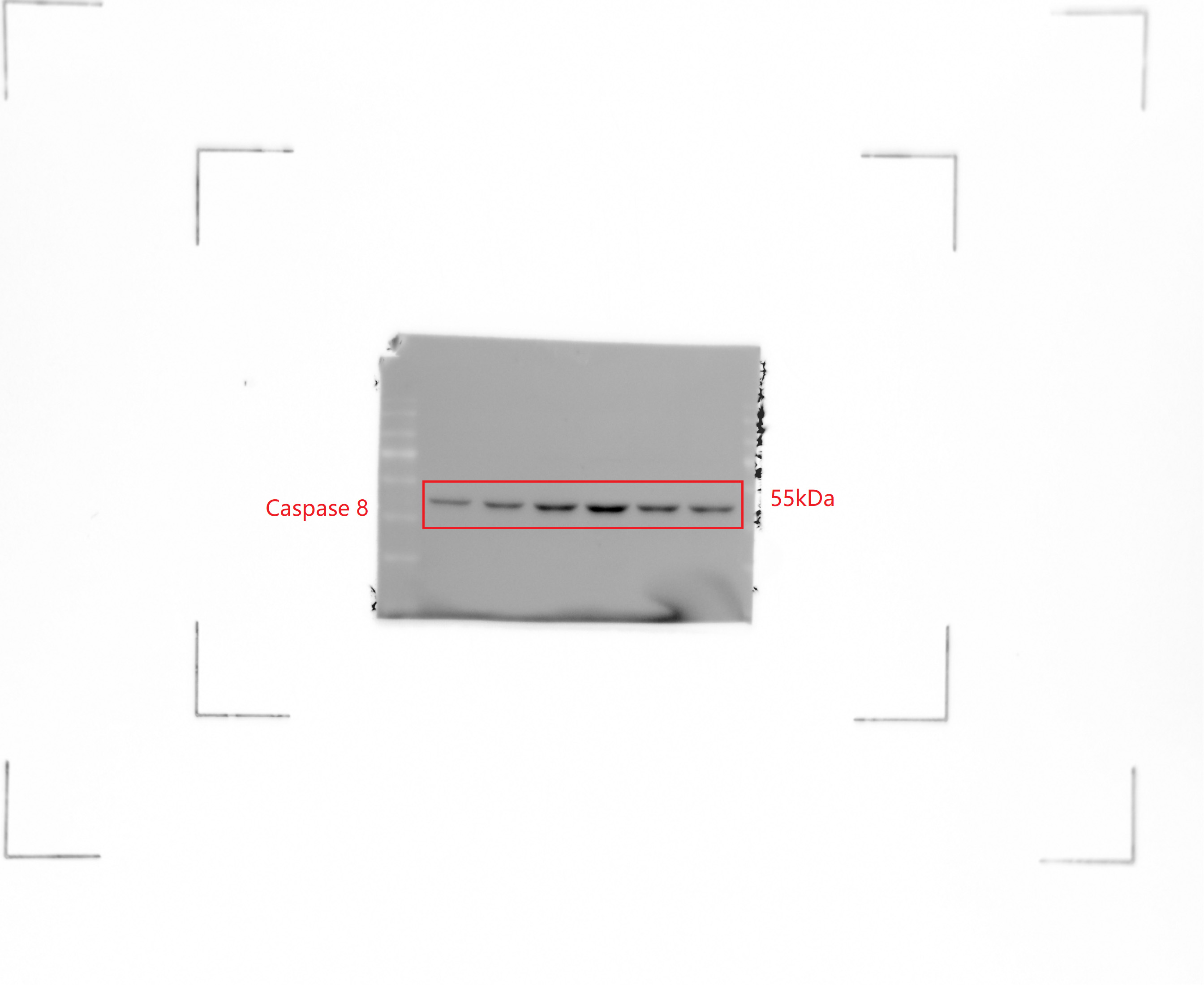

Supplement: Supplementary file 1 — Supplementary Material 1. [file 40001_2024_1968_MOESM1_ESM.zip › western blot original images/FIGURE4 orignal image/Caspase 8.jpg]

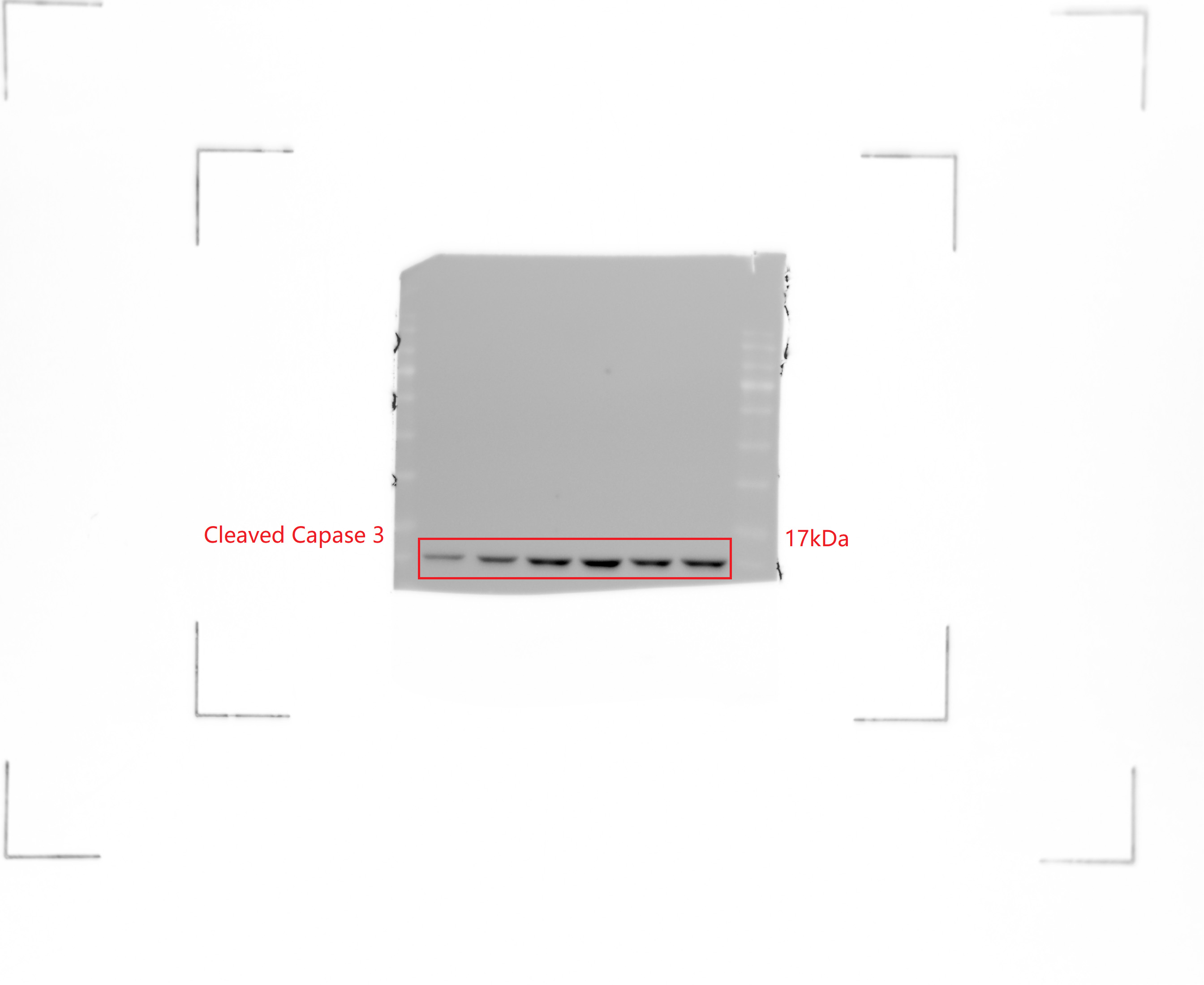

Supplement: Supplementary file 1 — Supplementary Material 1. [file 40001_2024_1968_MOESM1_ESM.zip › western blot original images/FIGURE4 orignal image/Cleaved Caspase 3.jpg]

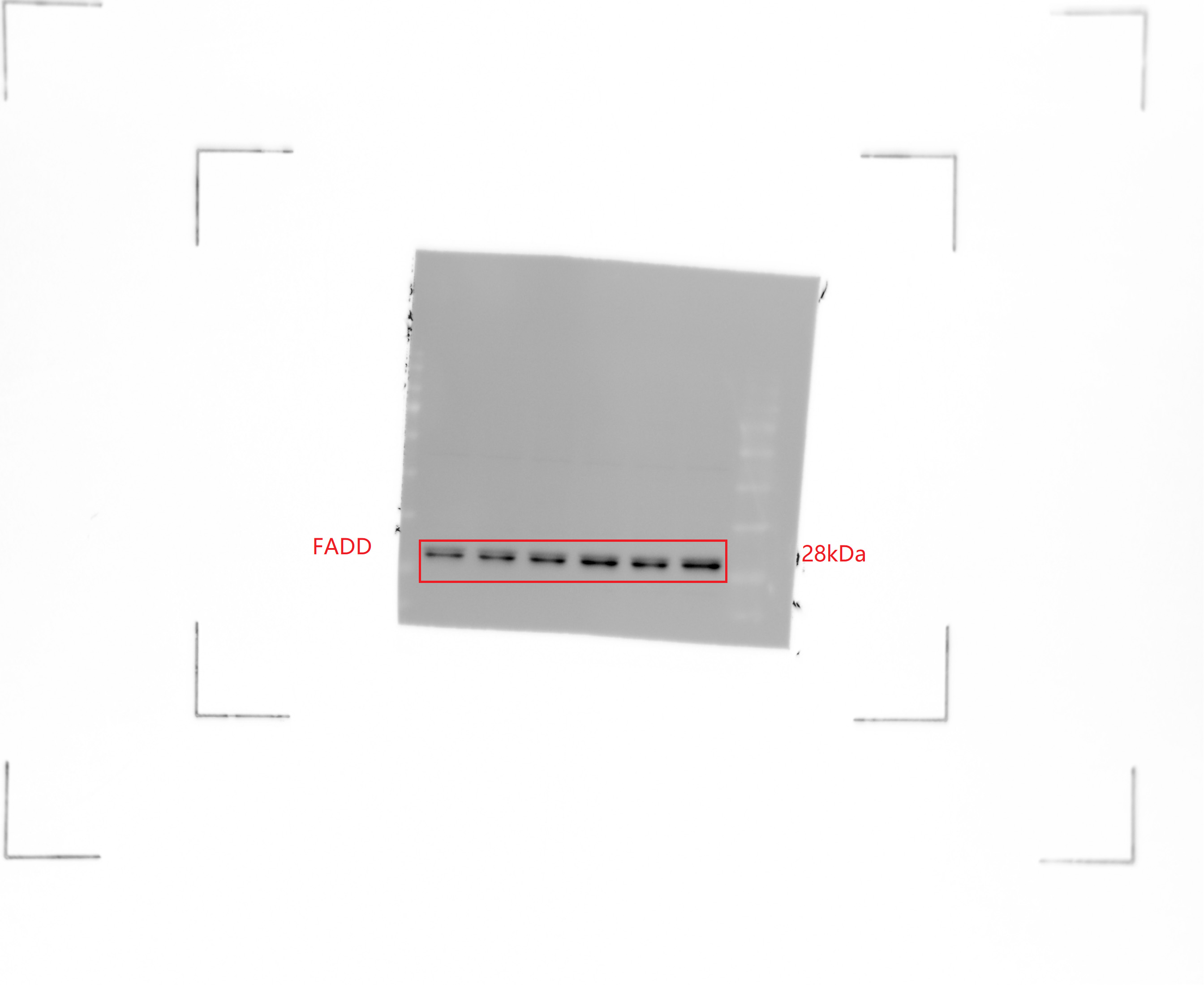

Supplement: Supplementary file 1 — Supplementary Material 1. [file 40001_2024_1968_MOESM1_ESM.zip › western blot original images/FIGURE4 orignal image/FADD.jpg]

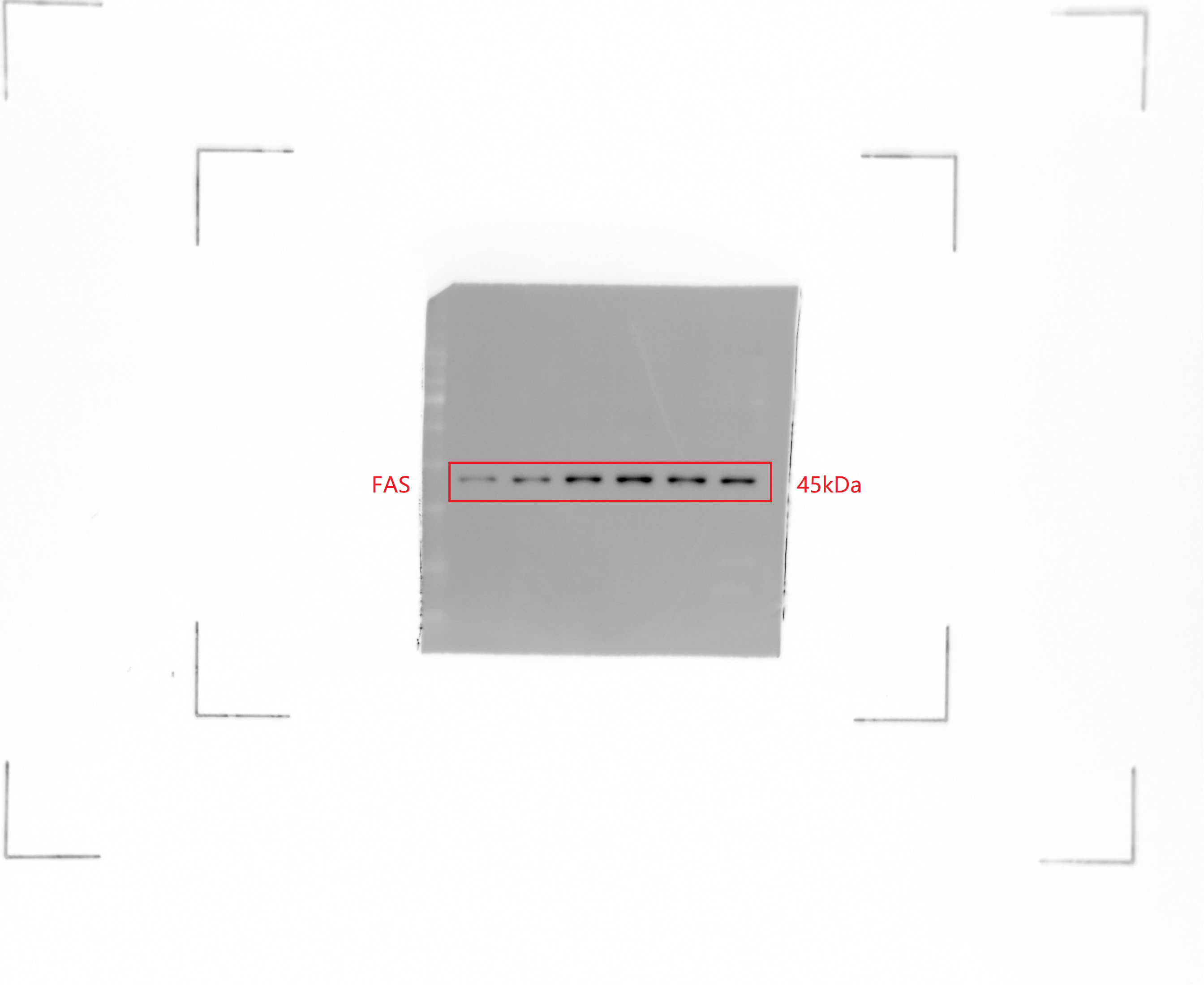

Supplement: Supplementary file 1 — Supplementary Material 1. [file 40001_2024_1968_MOESM1_ESM.zip › western blot original images/FIGURE4 orignal image/FAS.jpg]

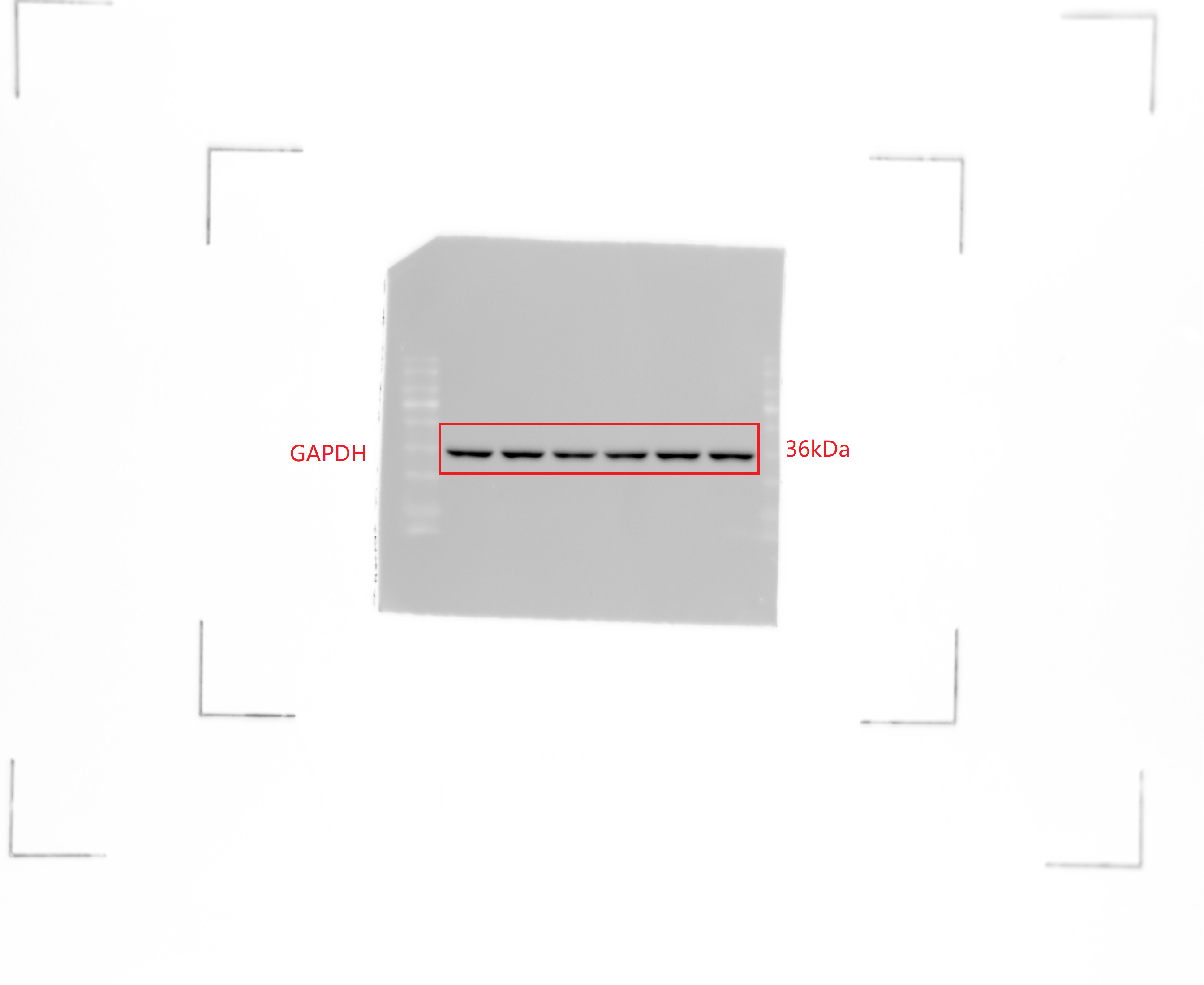

Supplement: Supplementary file 1 — Supplementary Material 1. [file 40001_2024_1968_MOESM1_ESM.zip › western blot original images/FIGURE4 orignal image/GAPDH.jpg]

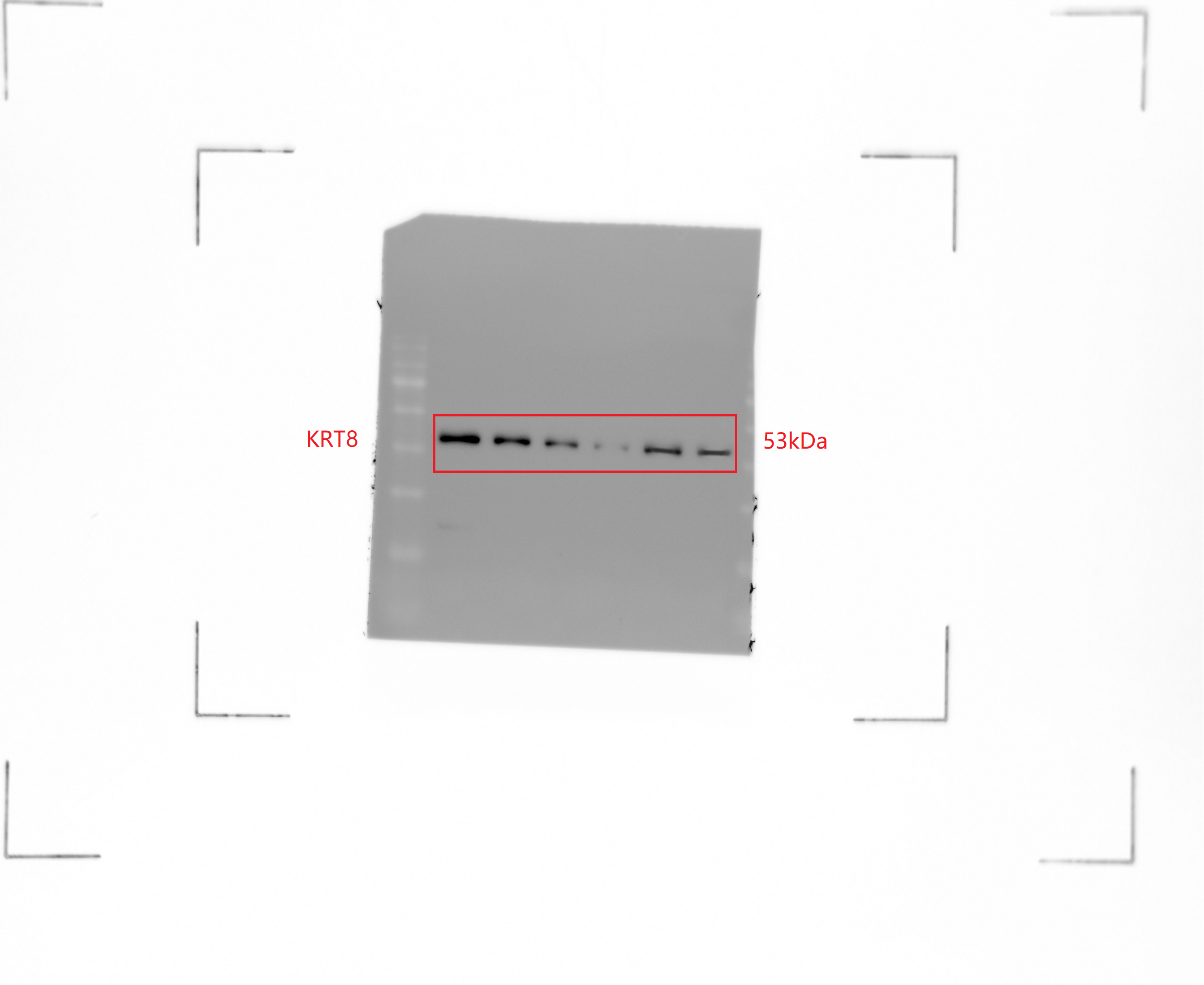

Supplement: Supplementary file 1 — Supplementary Material 1. [file 40001_2024_1968_MOESM1_ESM.zip › western blot original images/FIGURE4 orignal image/KRT8.jpg]

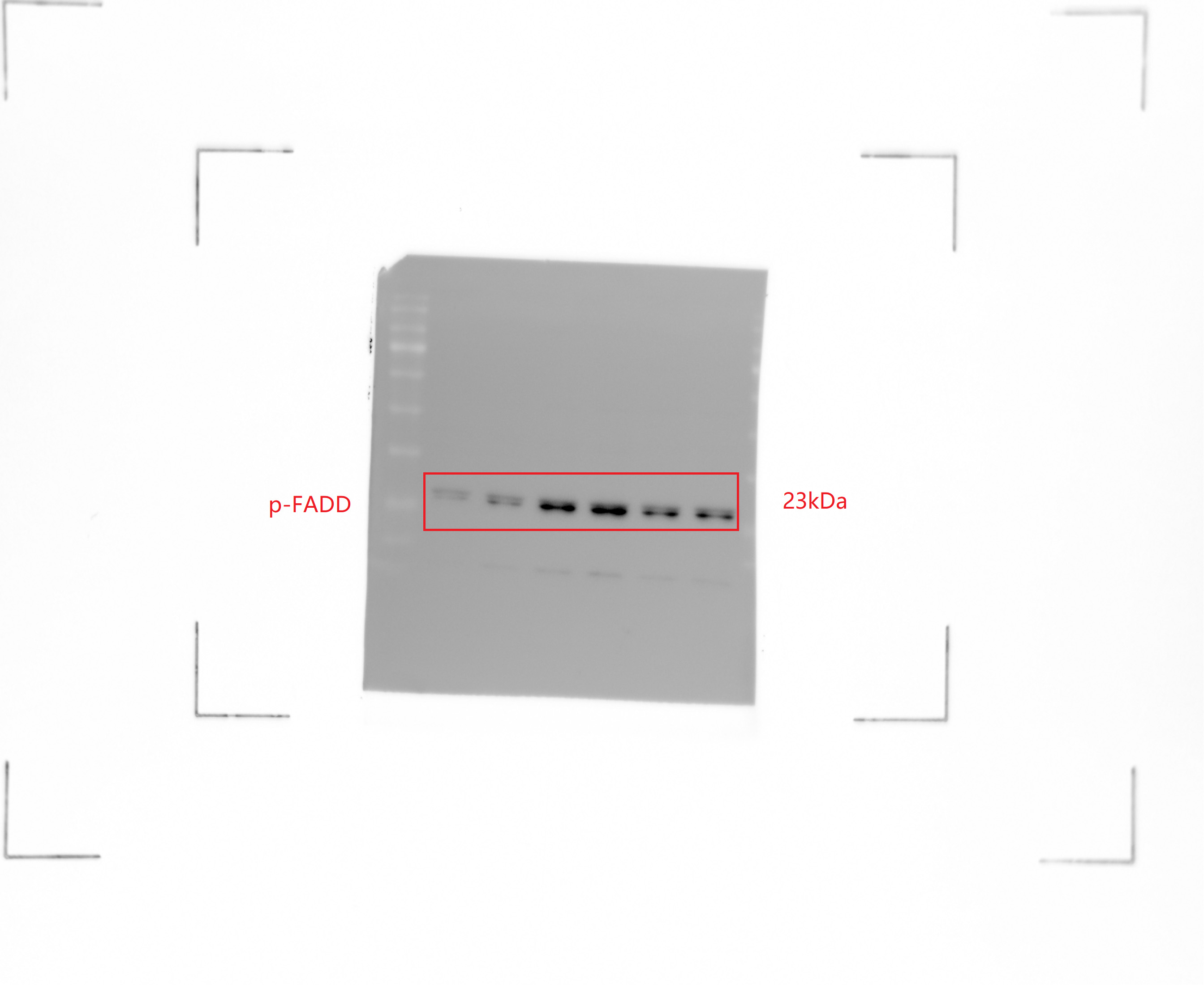

Supplement: Supplementary file 1 — Supplementary Material 1. [file 40001_2024_1968_MOESM1_ESM.zip › western blot original images/FIGURE4 orignal image/p-FADD.jpg]

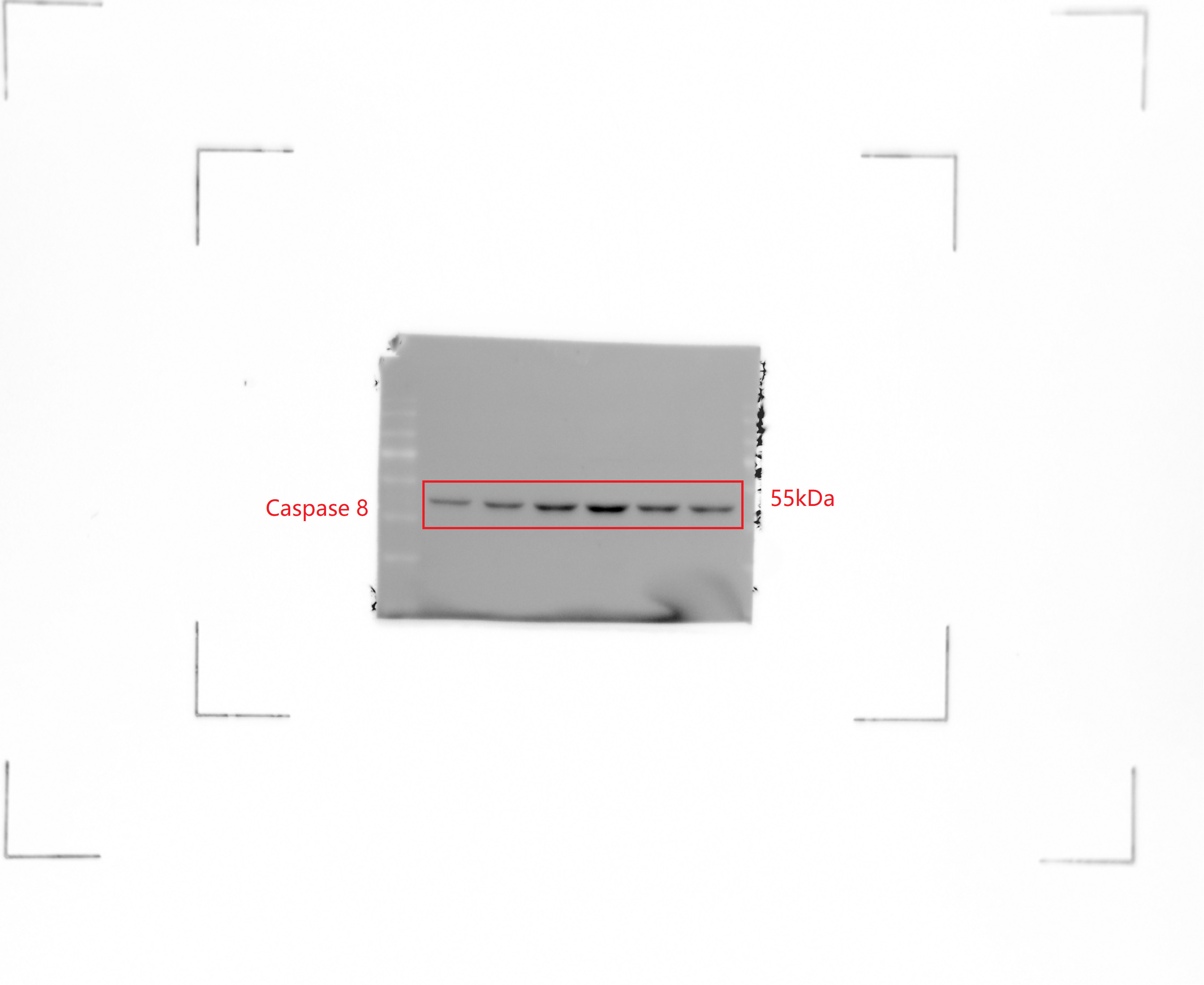

Supplement: Supplementary file 1 — Supplementary Material 1. [file 40001_2024_1968_MOESM1_ESM.zip › western blot original images/FIGURE7 original image/Caspase 8.jpg]

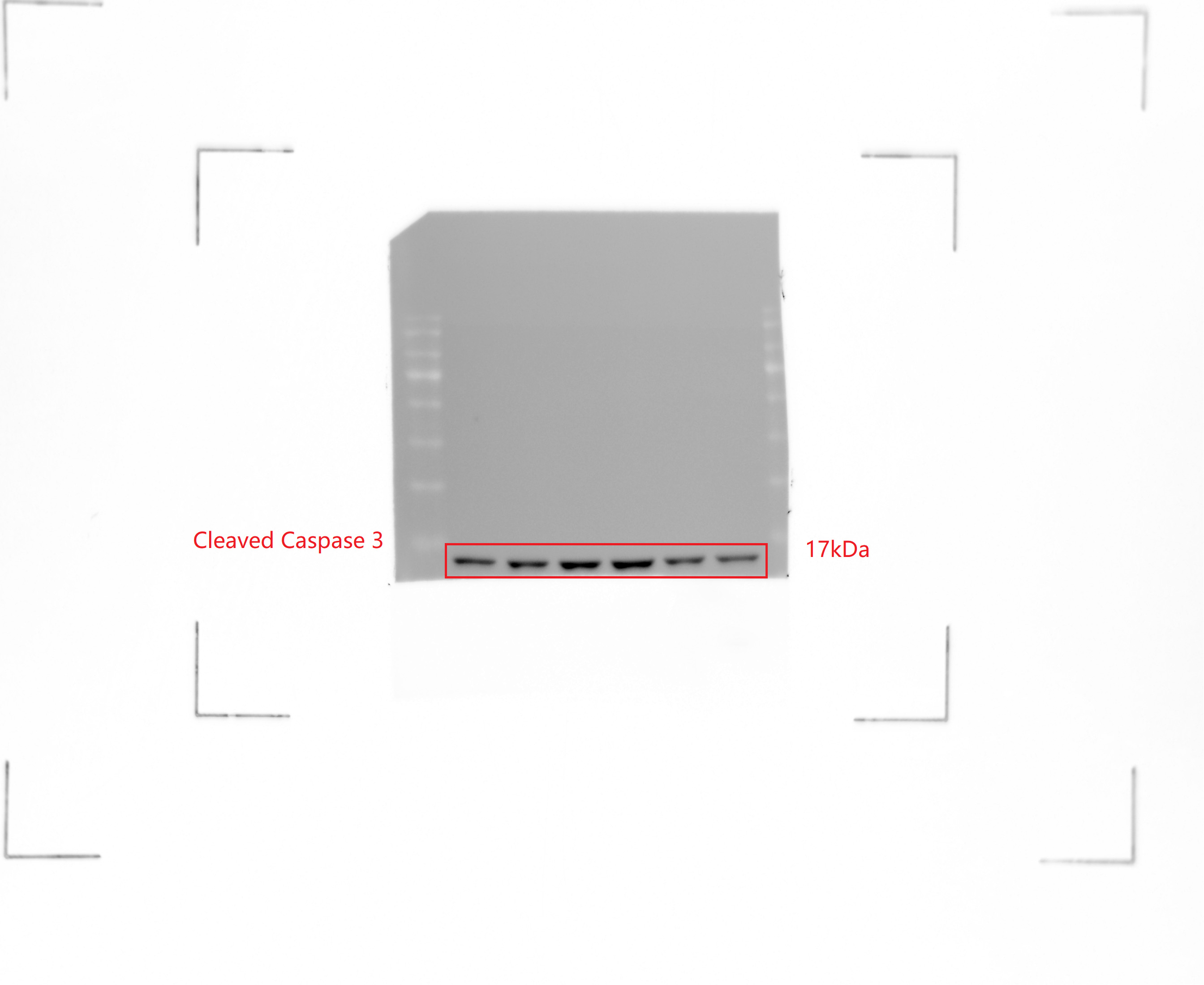

Supplement: Supplementary file 1 — Supplementary Material 1. [file 40001_2024_1968_MOESM1_ESM.zip › western blot original images/FIGURE7 original image/Cleaved Caspase 3.jpg]

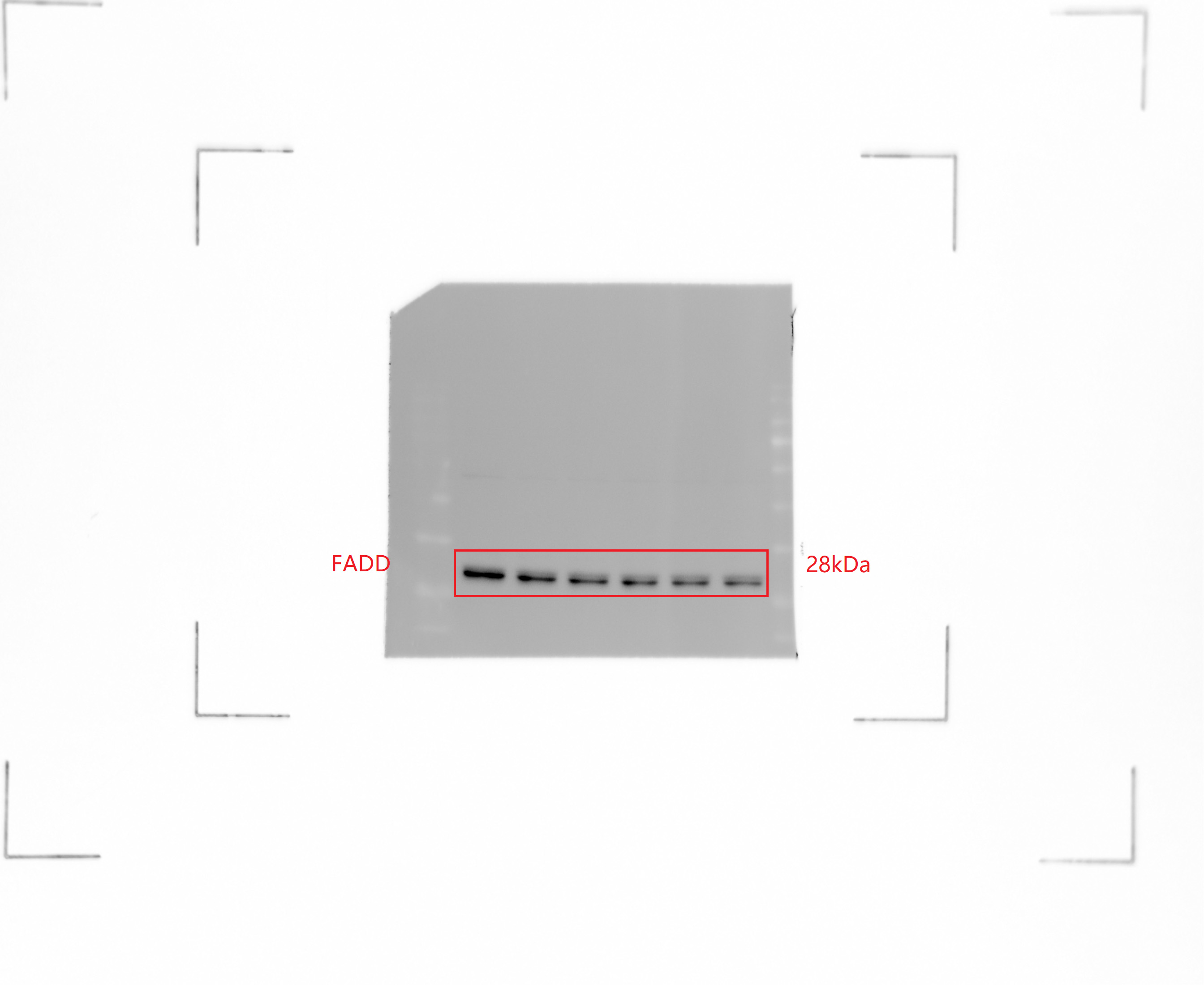

Supplement: Supplementary file 1 — Supplementary Material 1. [file 40001_2024_1968_MOESM1_ESM.zip › western blot original images/FIGURE7 original image/FADD.jpg]

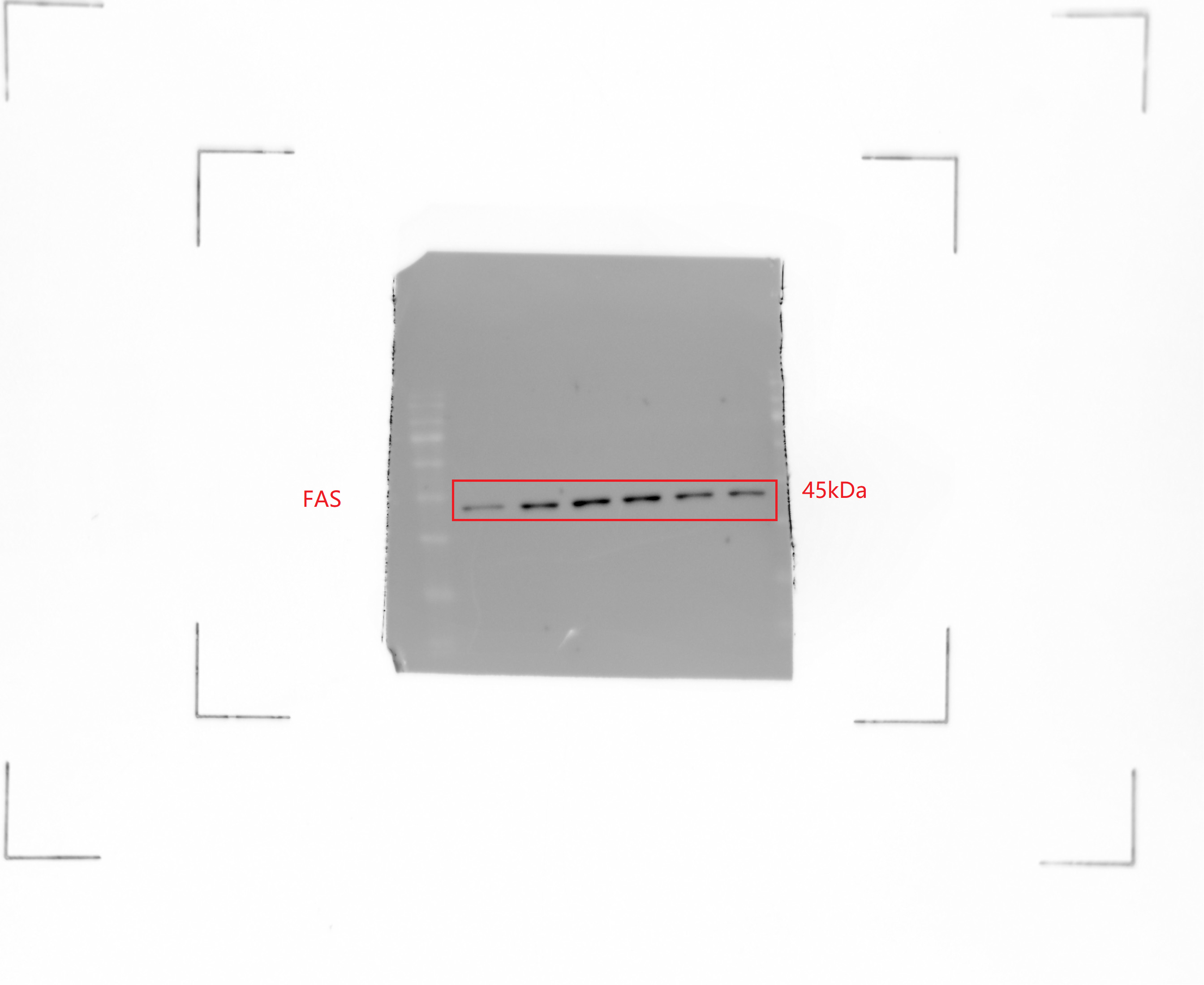

Supplement: Supplementary file 1 — Supplementary Material 1. [file 40001_2024_1968_MOESM1_ESM.zip › western blot original images/FIGURE7 original image/FAS.jpg]

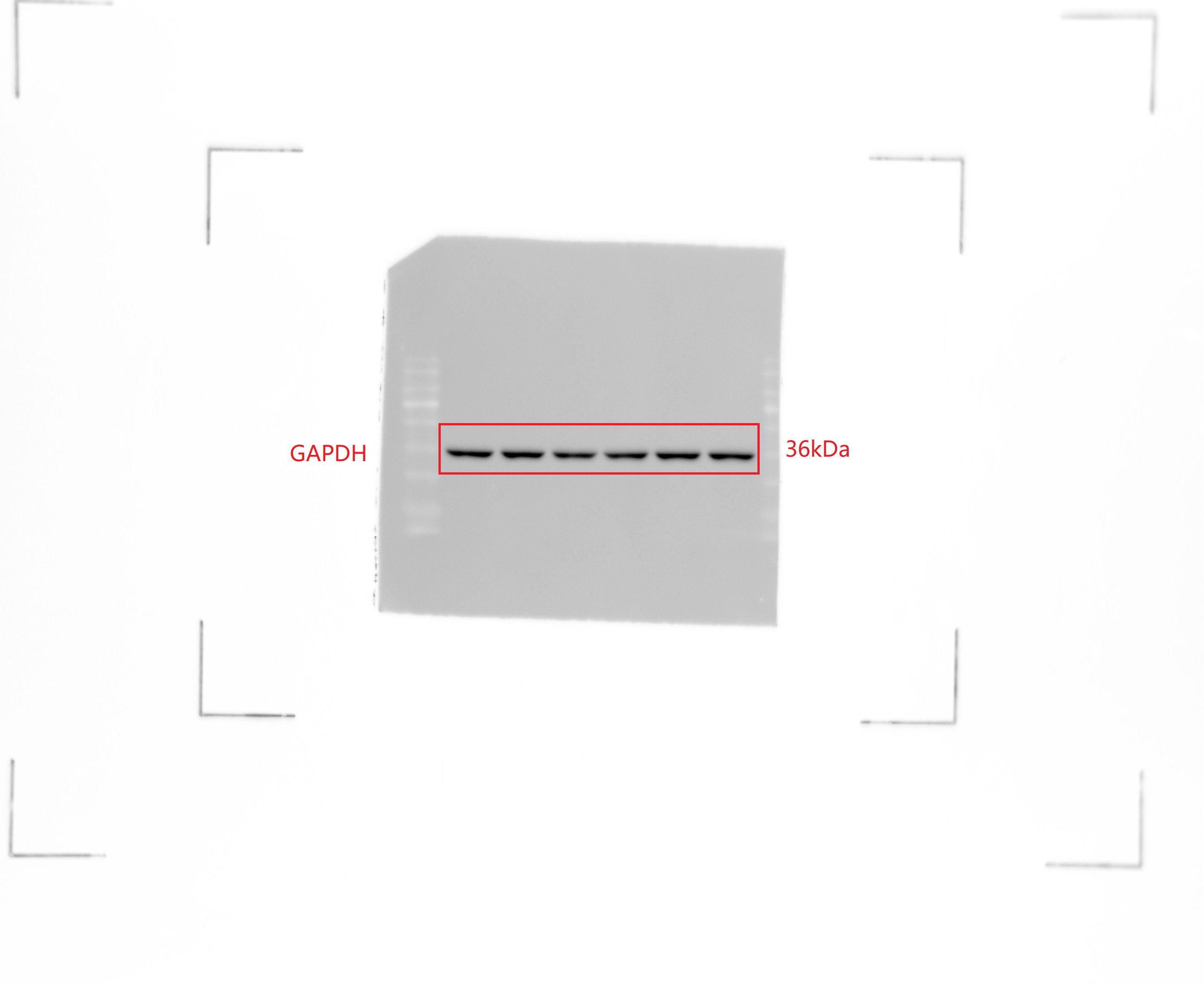

Supplement: Supplementary file 1 — Supplementary Material 1. [file 40001_2024_1968_MOESM1_ESM.zip › western blot original images/FIGURE7 original image/GAPDH.jpg]

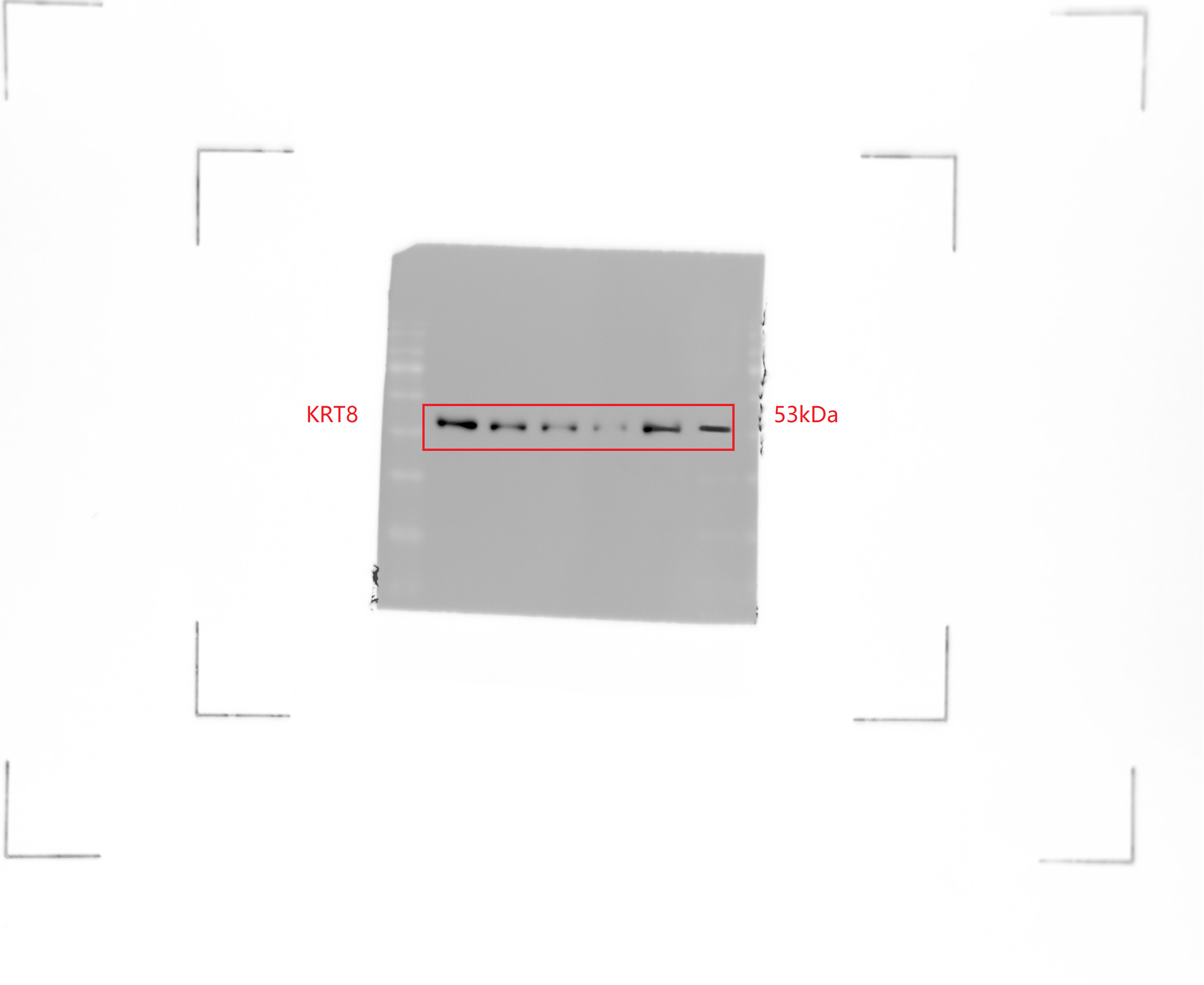

Supplement: Supplementary file 1 — Supplementary Material 1. [file 40001_2024_1968_MOESM1_ESM.zip › western blot original images/FIGURE7 original image/KRT8.jpg]

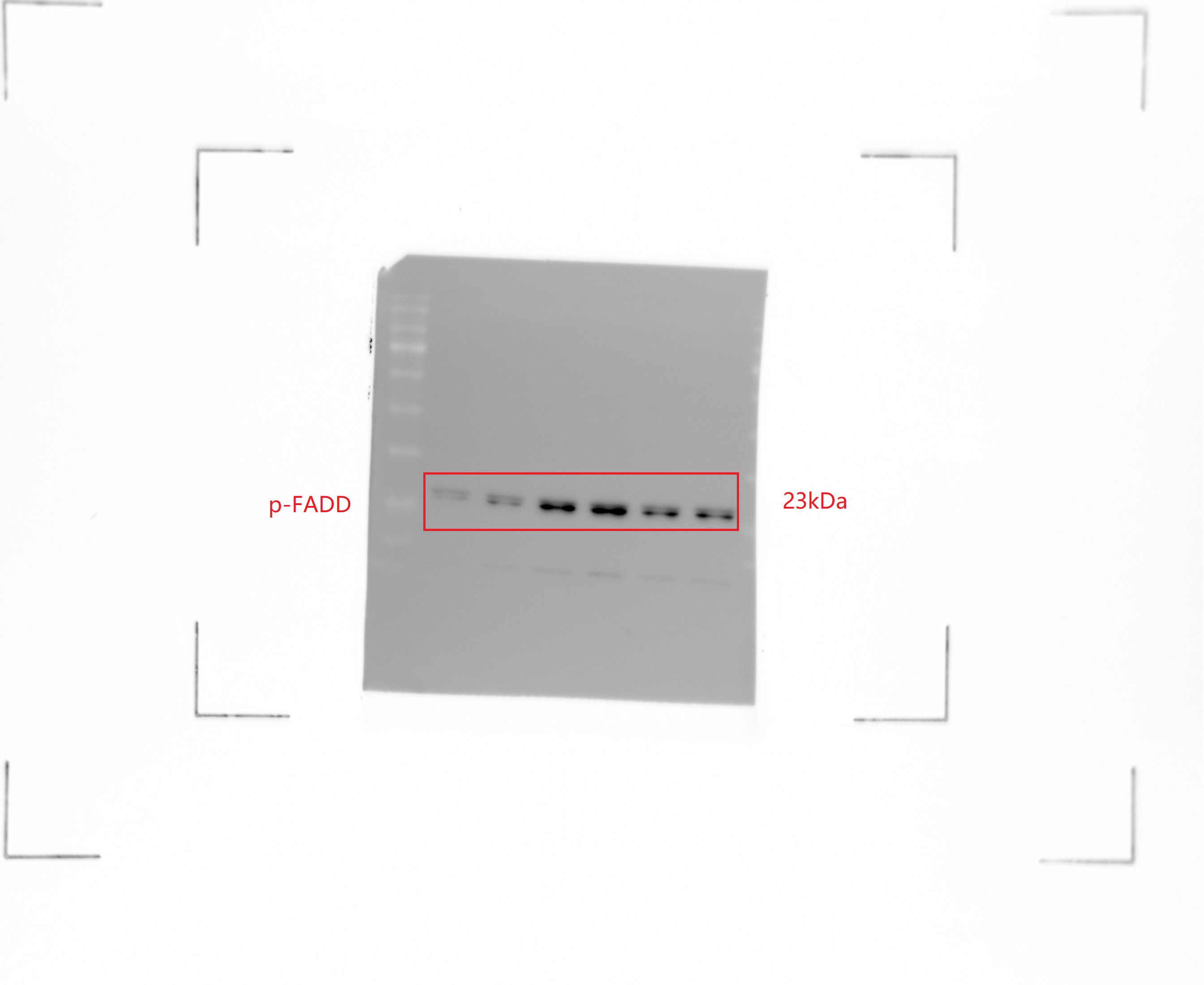

Supplement: Supplementary file 1 — Supplementary Material 1. [file 40001_2024_1968_MOESM1_ESM.zip › western blot original images/FIGURE7 original image/p-FADD.jpg]

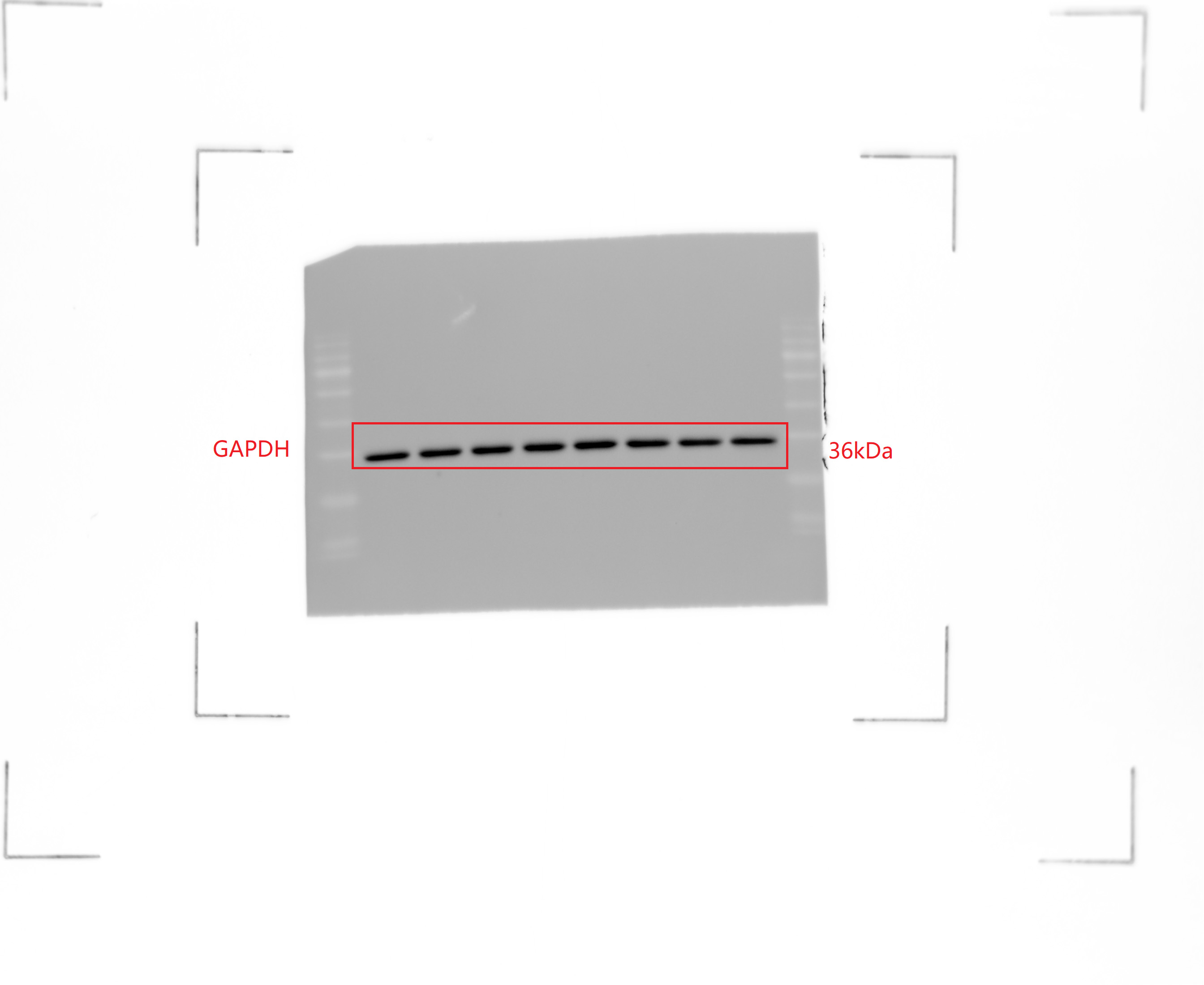

Supplement: Supplementary file 1 — Supplementary Material 1. [file 40001_2024_1968_MOESM1_ESM.zip › western blot original images/FIRUGE2 original image/2A-GAPDH.jpg]

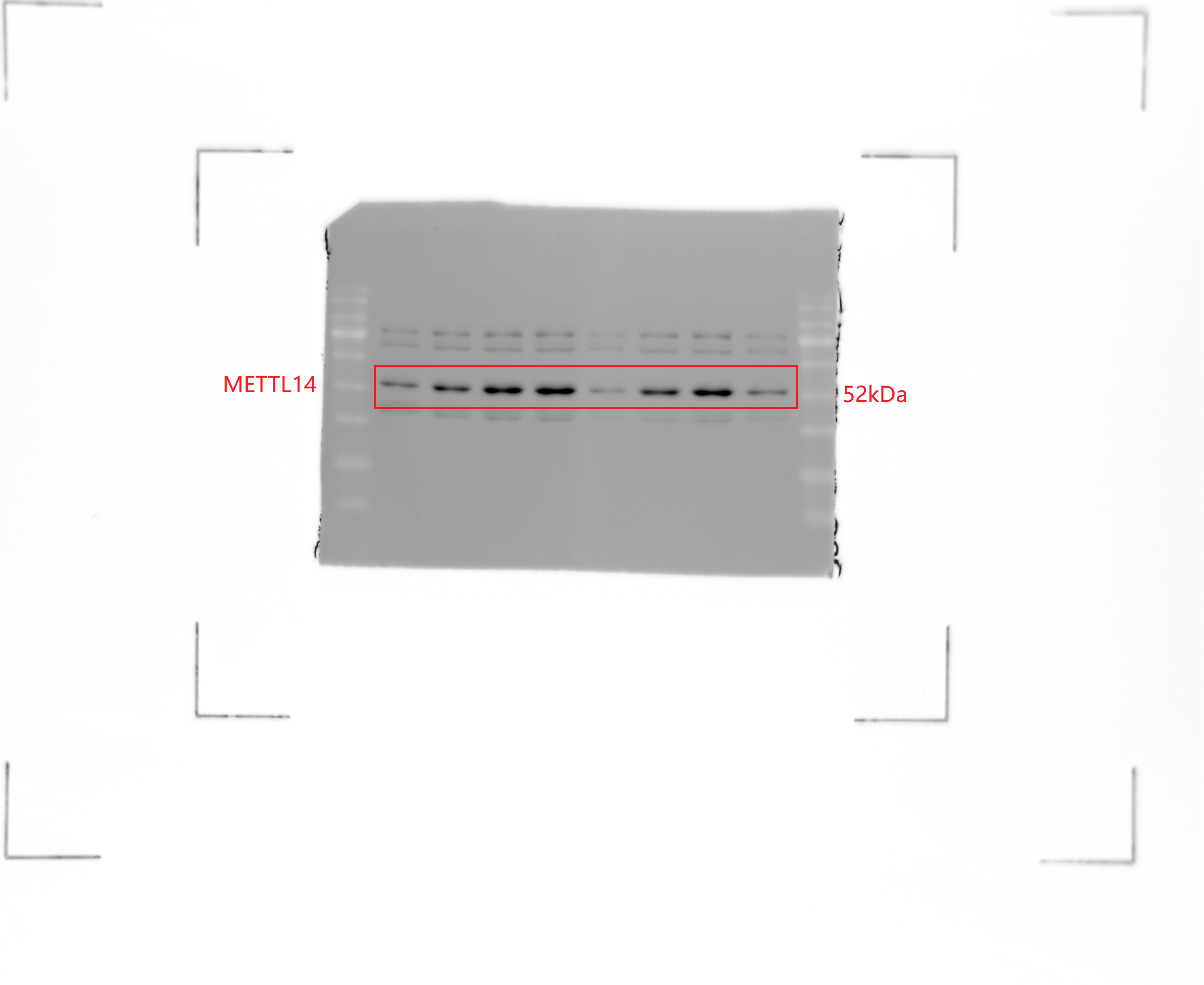

Supplement: Supplementary file 1 — Supplementary Material 1. [file 40001_2024_1968_MOESM1_ESM.zip › western blot original images/FIRUGE2 original image/2A-METTL14.jpg]

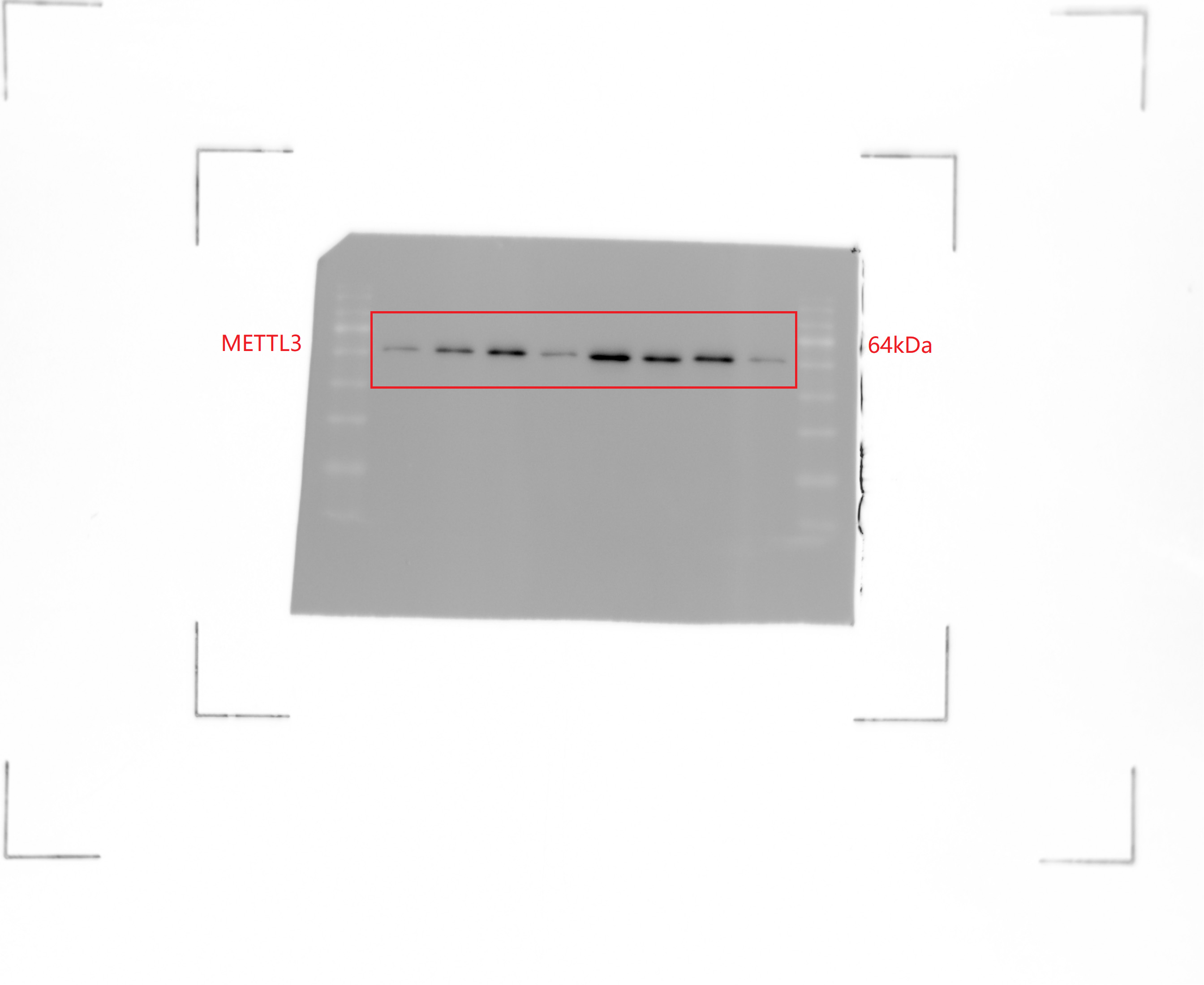

Supplement: Supplementary file 1 — Supplementary Material 1. [file 40001_2024_1968_MOESM1_ESM.zip › western blot original images/FIRUGE2 original image/2A-METTL3.jpg]

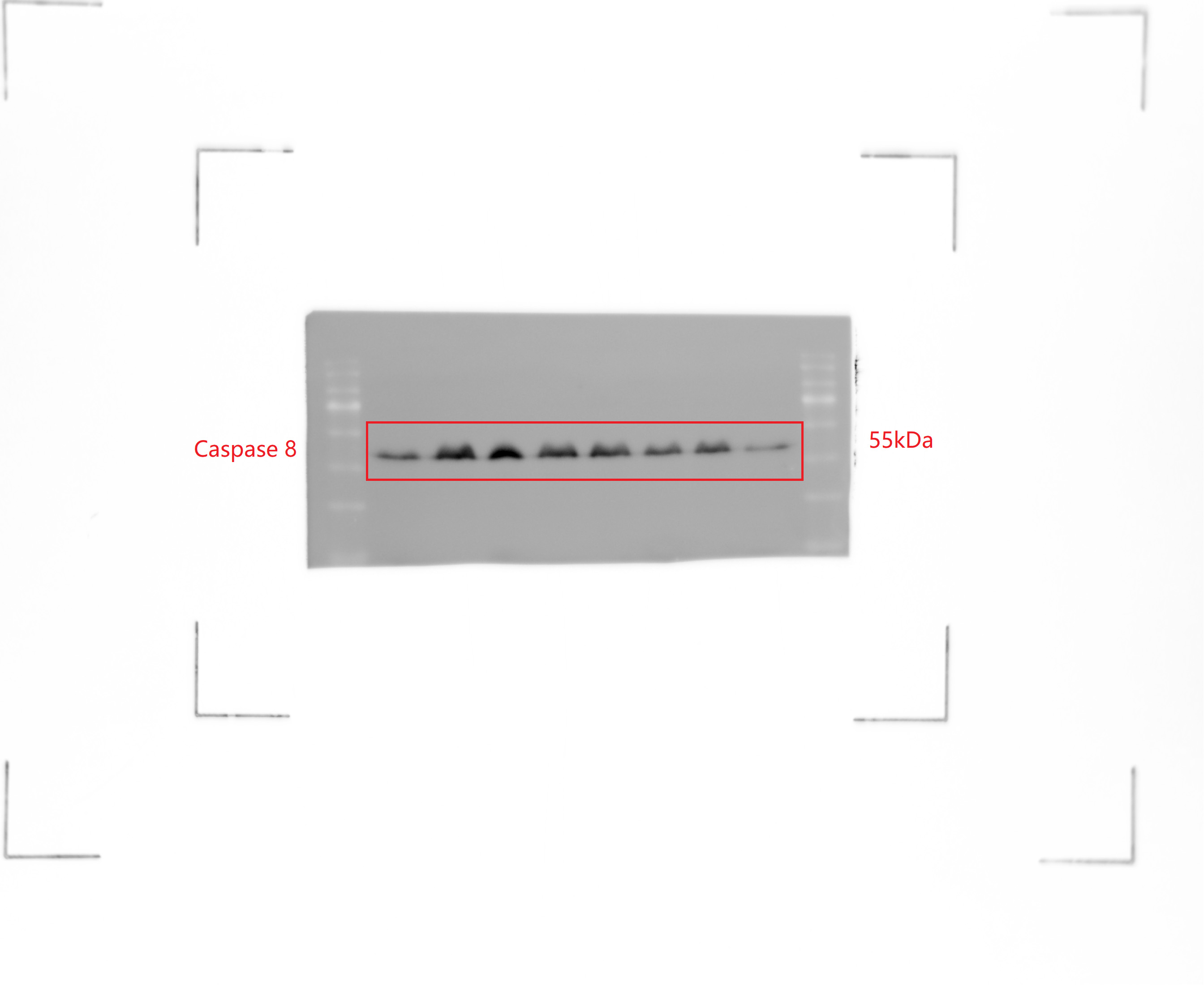

Supplement: Supplementary file 1 — Supplementary Material 1. [file 40001_2024_1968_MOESM1_ESM.zip › western blot original images/FIRUGE2 original image/2E-Caspase 8.jpg]

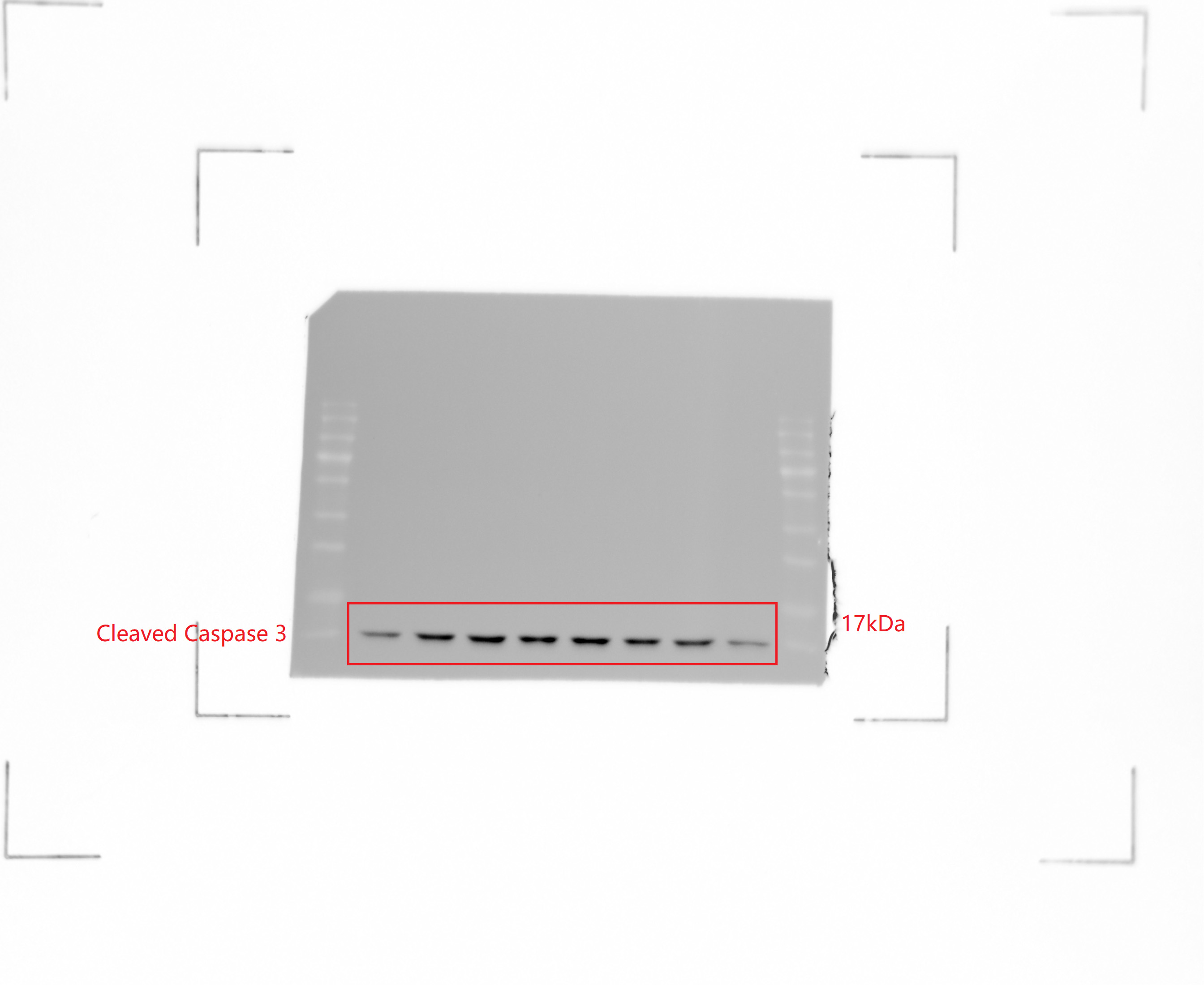

Supplement: Supplementary file 1 — Supplementary Material 1. [file 40001_2024_1968_MOESM1_ESM.zip › western blot original images/FIRUGE2 original image/2E-Cleaved Caspase 3.jpg]

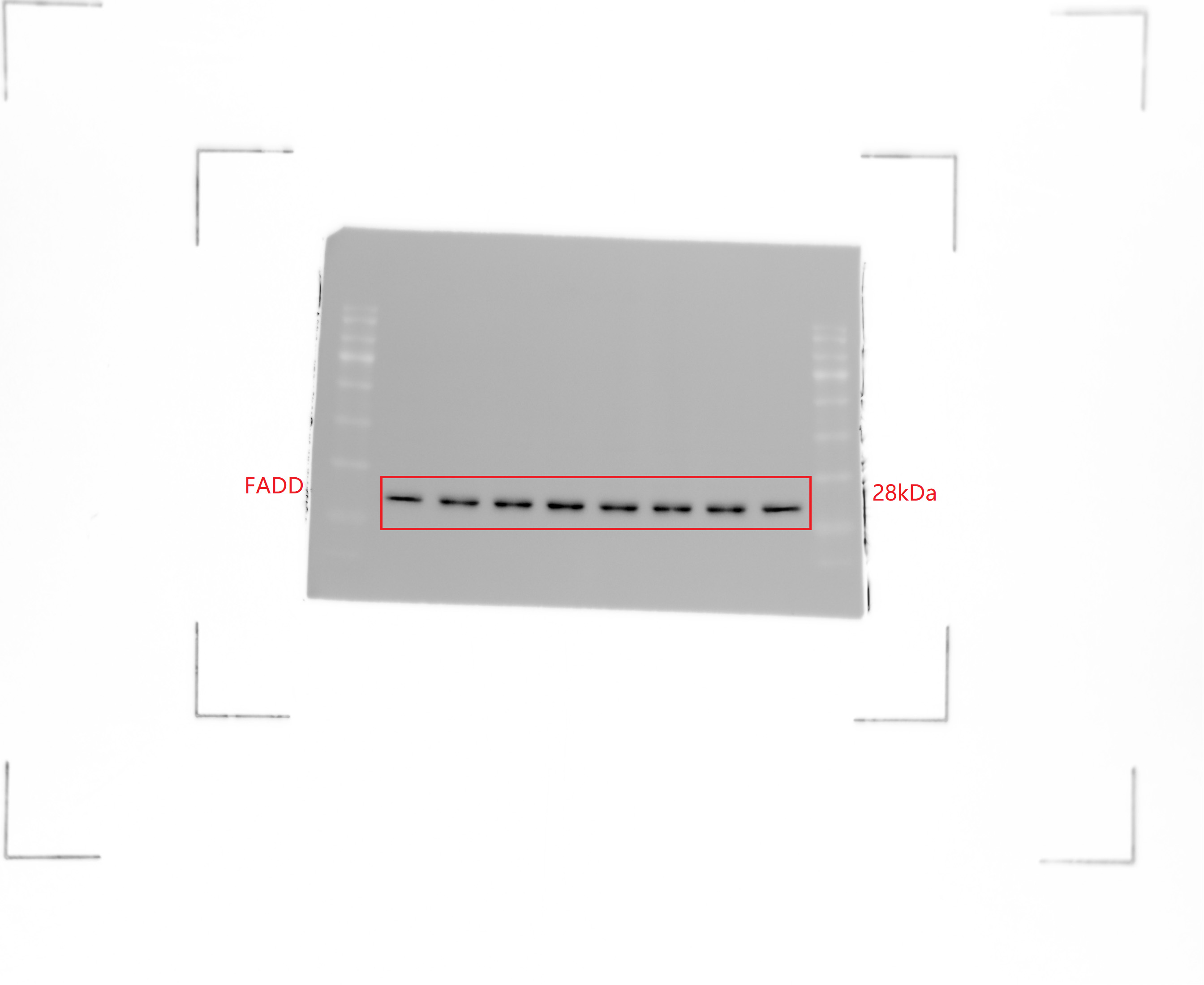

Supplement: Supplementary file 1 — Supplementary Material 1. [file 40001_2024_1968_MOESM1_ESM.zip › western blot original images/FIRUGE2 original image/2E-FADD.jpg]

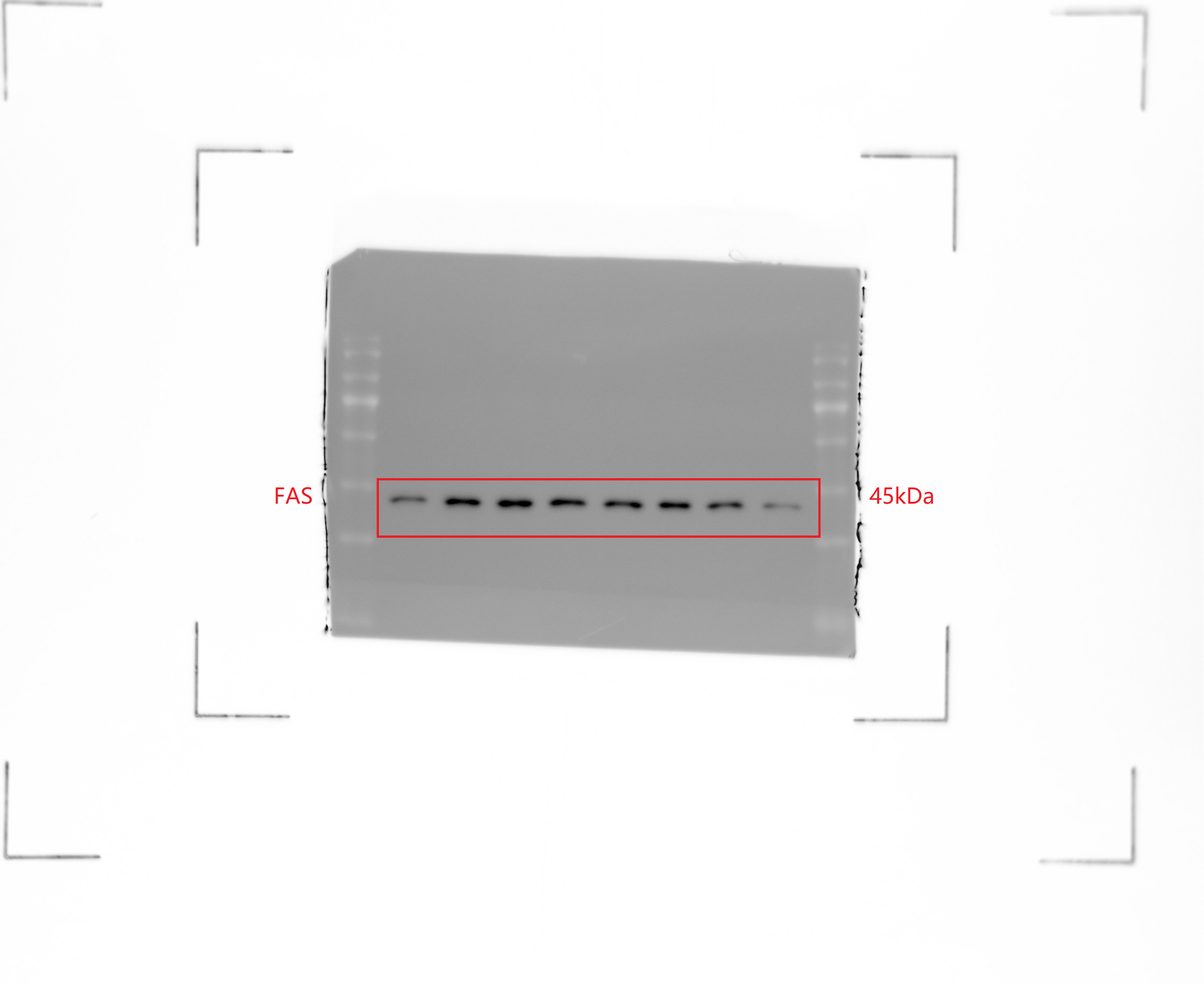

Supplement: Supplementary file 1 — Supplementary Material 1. [file 40001_2024_1968_MOESM1_ESM.zip › western blot original images/FIRUGE2 original image/2E-FAS.jpg]

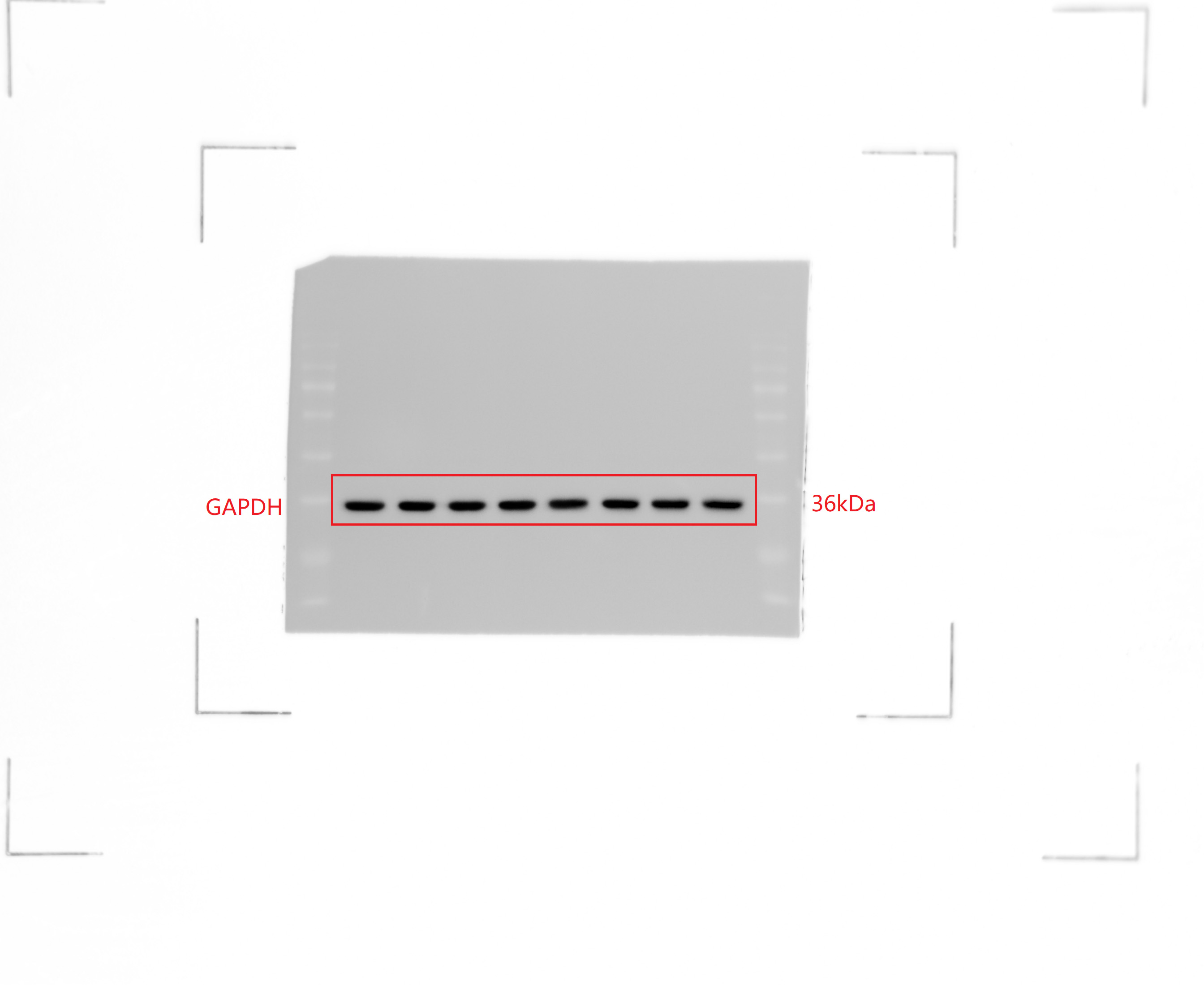

Supplement: Supplementary file 1 — Supplementary Material 1. [file 40001_2024_1968_MOESM1_ESM.zip › western blot original images/FIRUGE2 original image/2E-GAPDH.jpg]

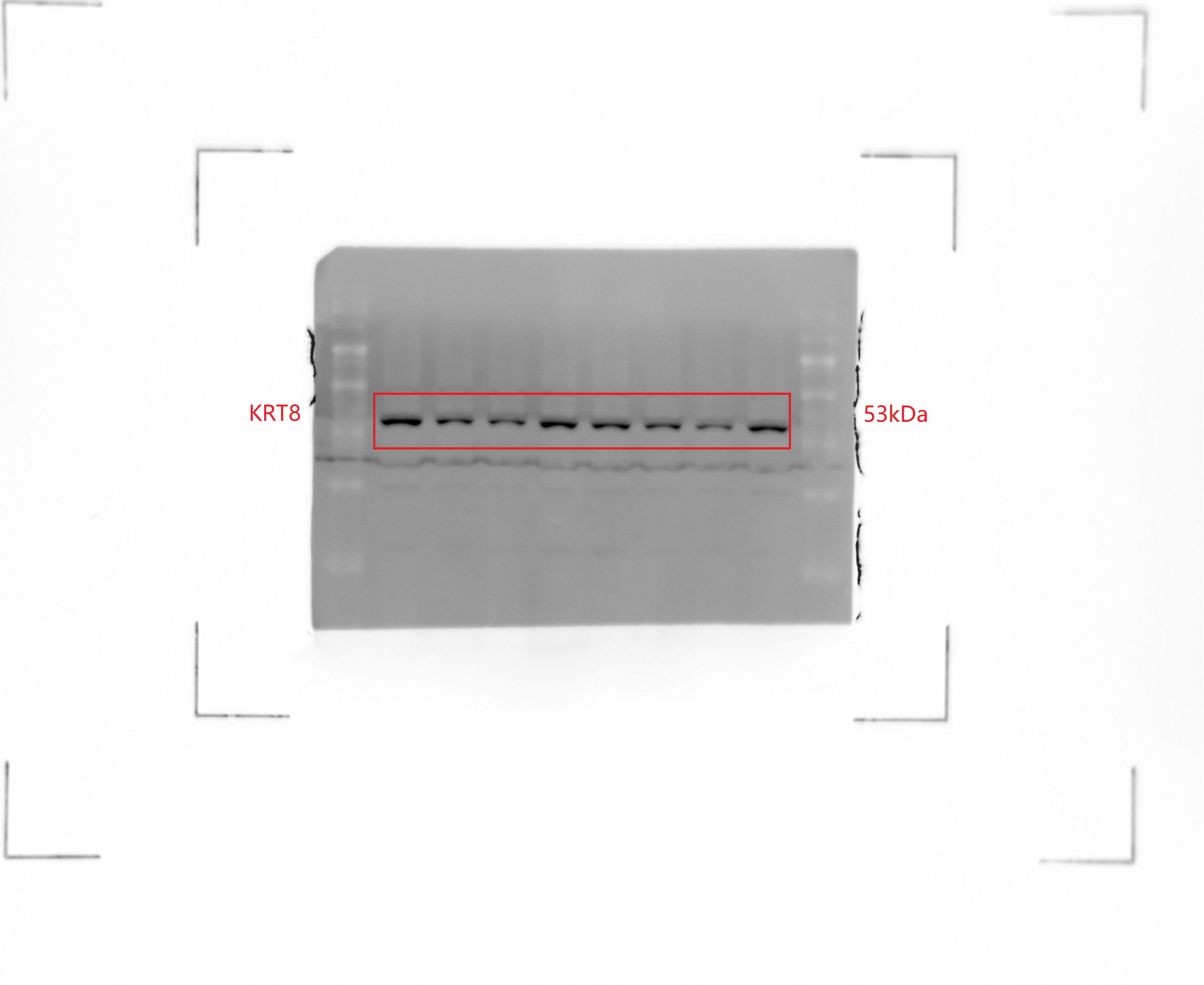

Supplement: Supplementary file 1 — Supplementary Material 1. [file 40001_2024_1968_MOESM1_ESM.zip › western blot original images/FIRUGE2 original image/2E-KRT8.jpg]

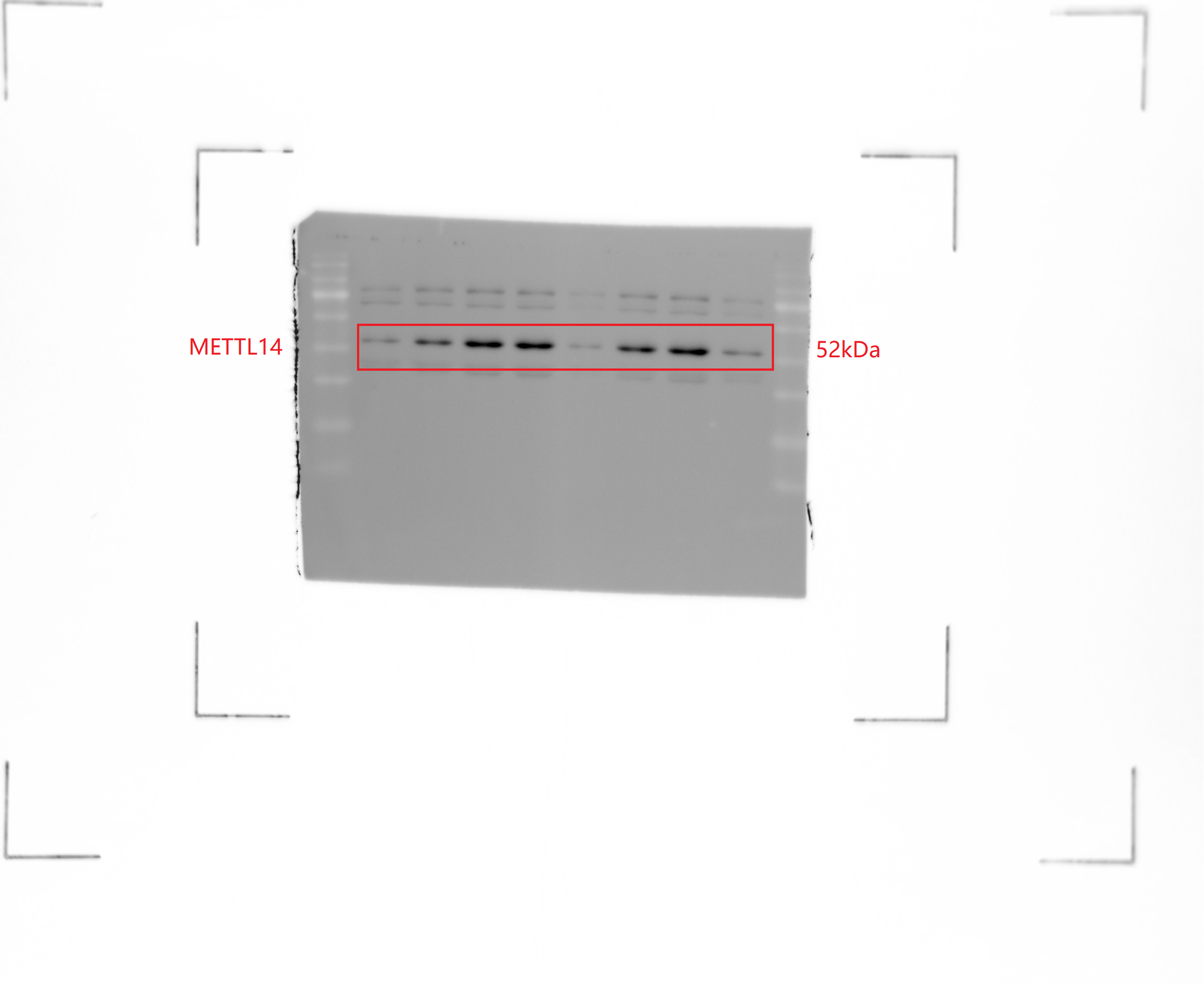

Supplement: Supplementary file 1 — Supplementary Material 1. [file 40001_2024_1968_MOESM1_ESM.zip › western blot original images/FIRUGE2 original image/2E-METTL14.jpg]

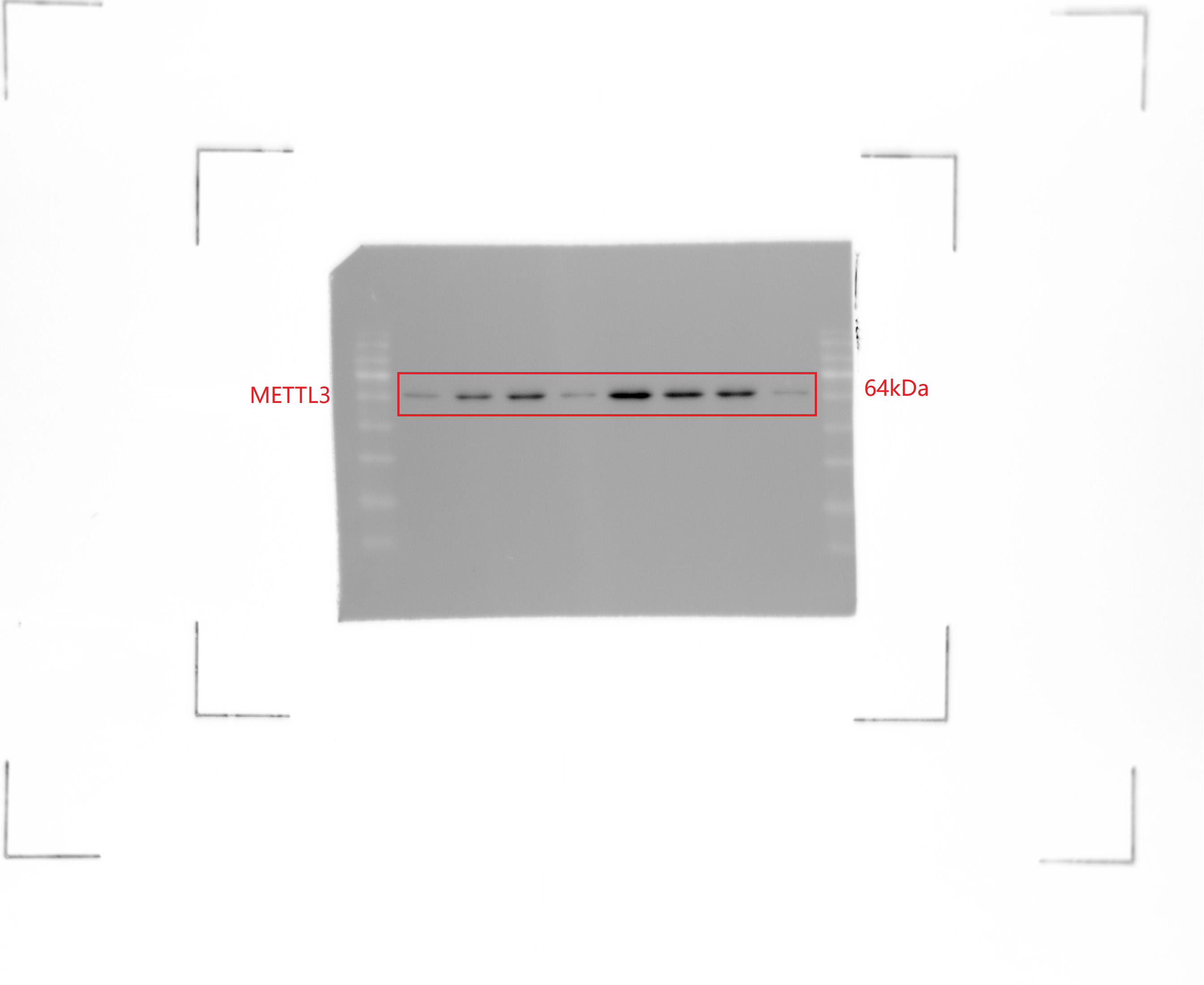

Supplement: Supplementary file 1 — Supplementary Material 1. [file 40001_2024_1968_MOESM1_ESM.zip › western blot original images/FIRUGE2 original image/2E-METTL3.jpg]

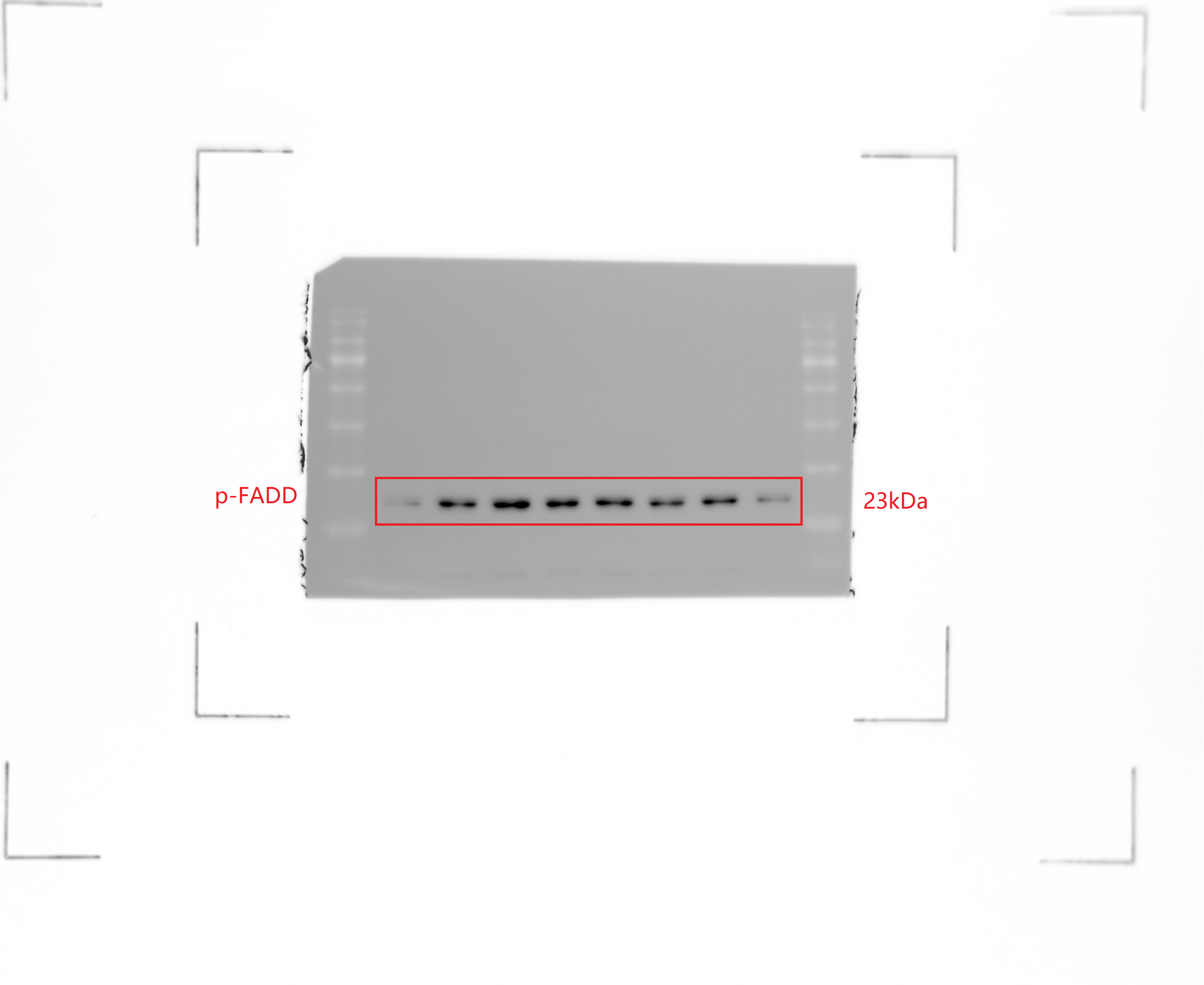

Supplement: Supplementary file 1 — Supplementary Material 1. [file 40001_2024_1968_MOESM1_ESM.zip › western blot original images/FIRUGE2 original image/2E-p-FADD.jpg]

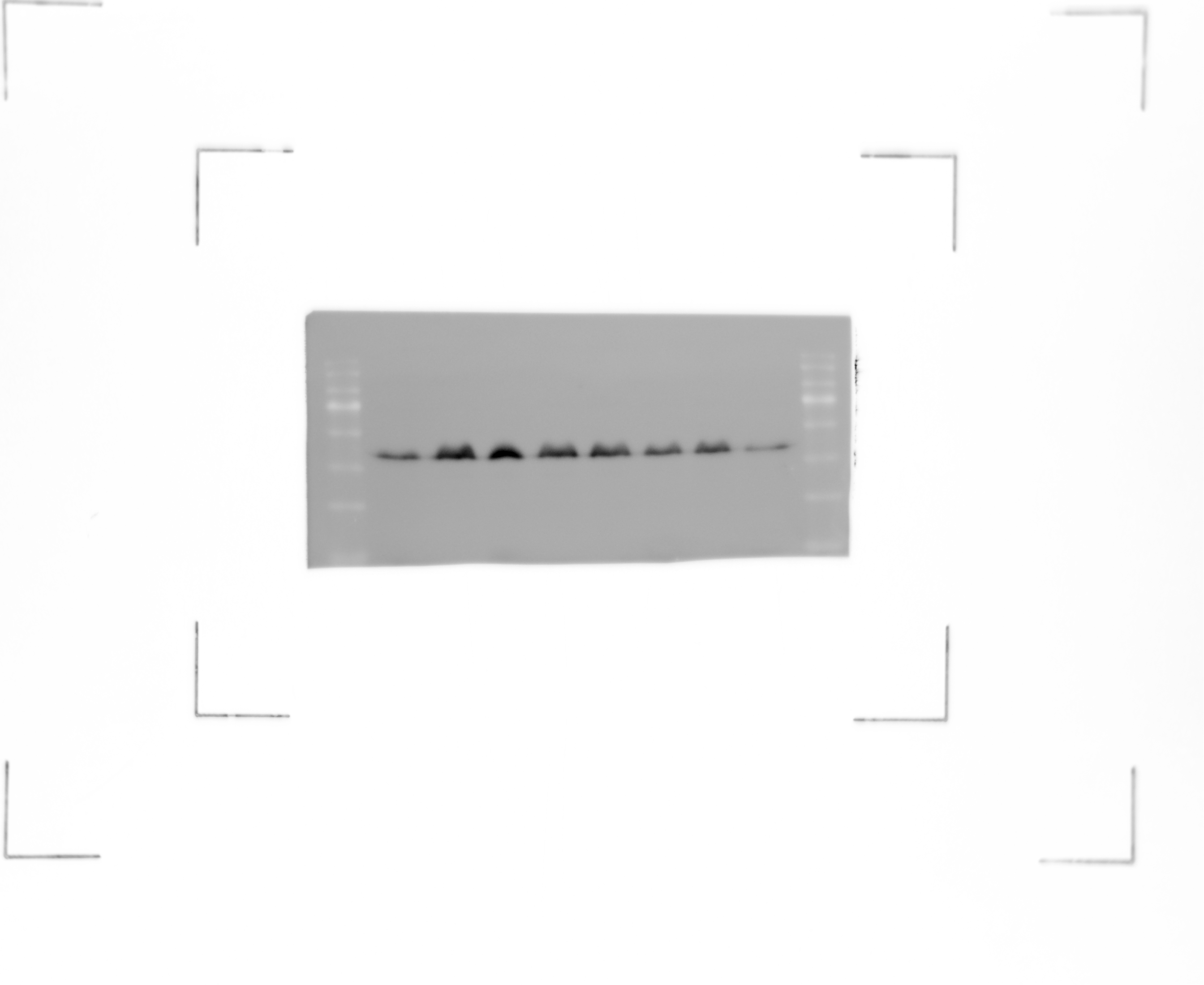

Supplement: Supplementary file 1 — Supplementary Material 1. [file 40001_2024_1968_MOESM1_ESM.zip › western blot original images/original images for all western blots/FIGURES1-3/Caspase 8-1.tif]

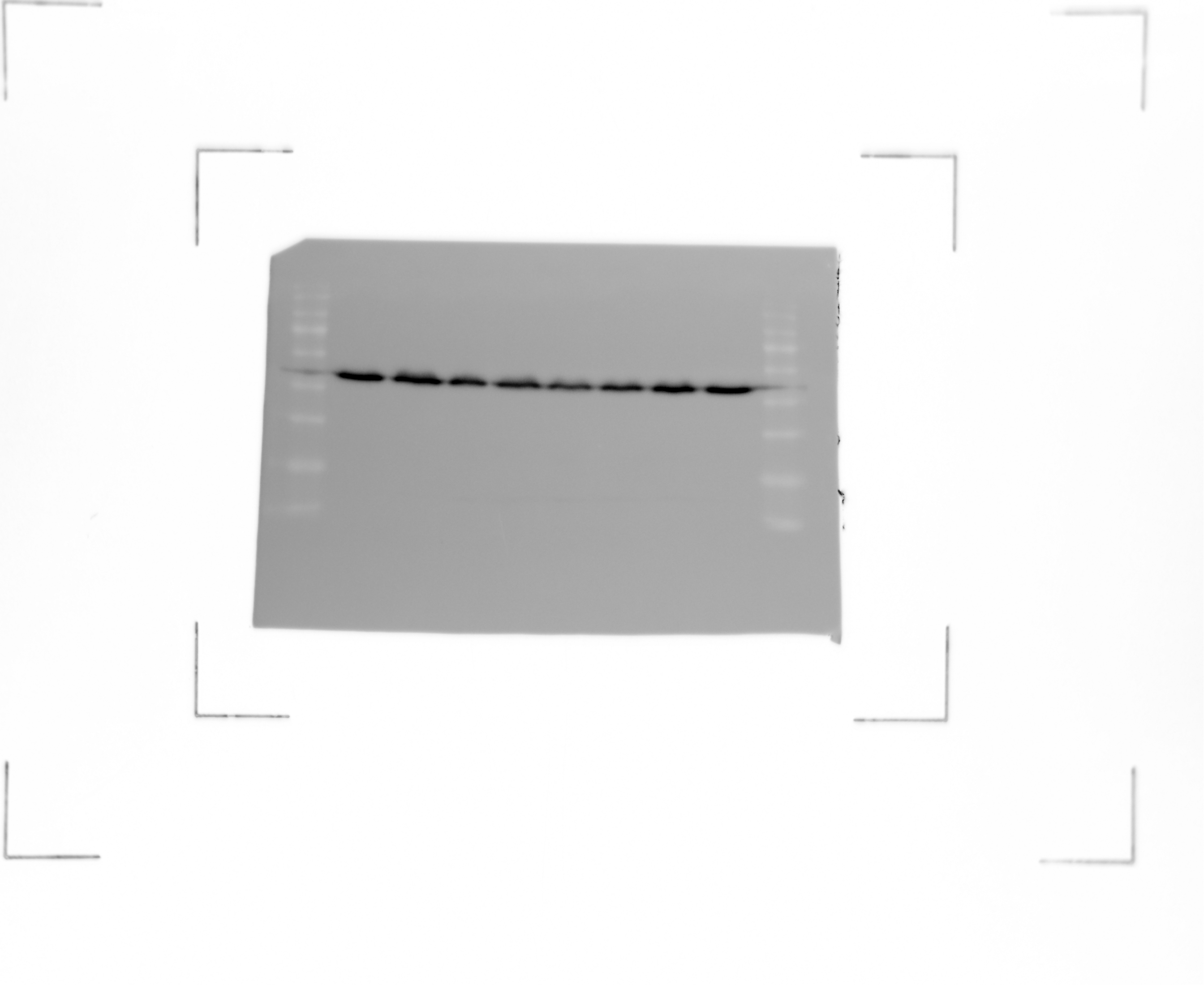

Supplement: Supplementary file 1 — Supplementary Material 1. [file 40001_2024_1968_MOESM1_ESM.zip › western blot original images/original images for all western blots/FIGURES1-3/Caspase 8-2.tif]

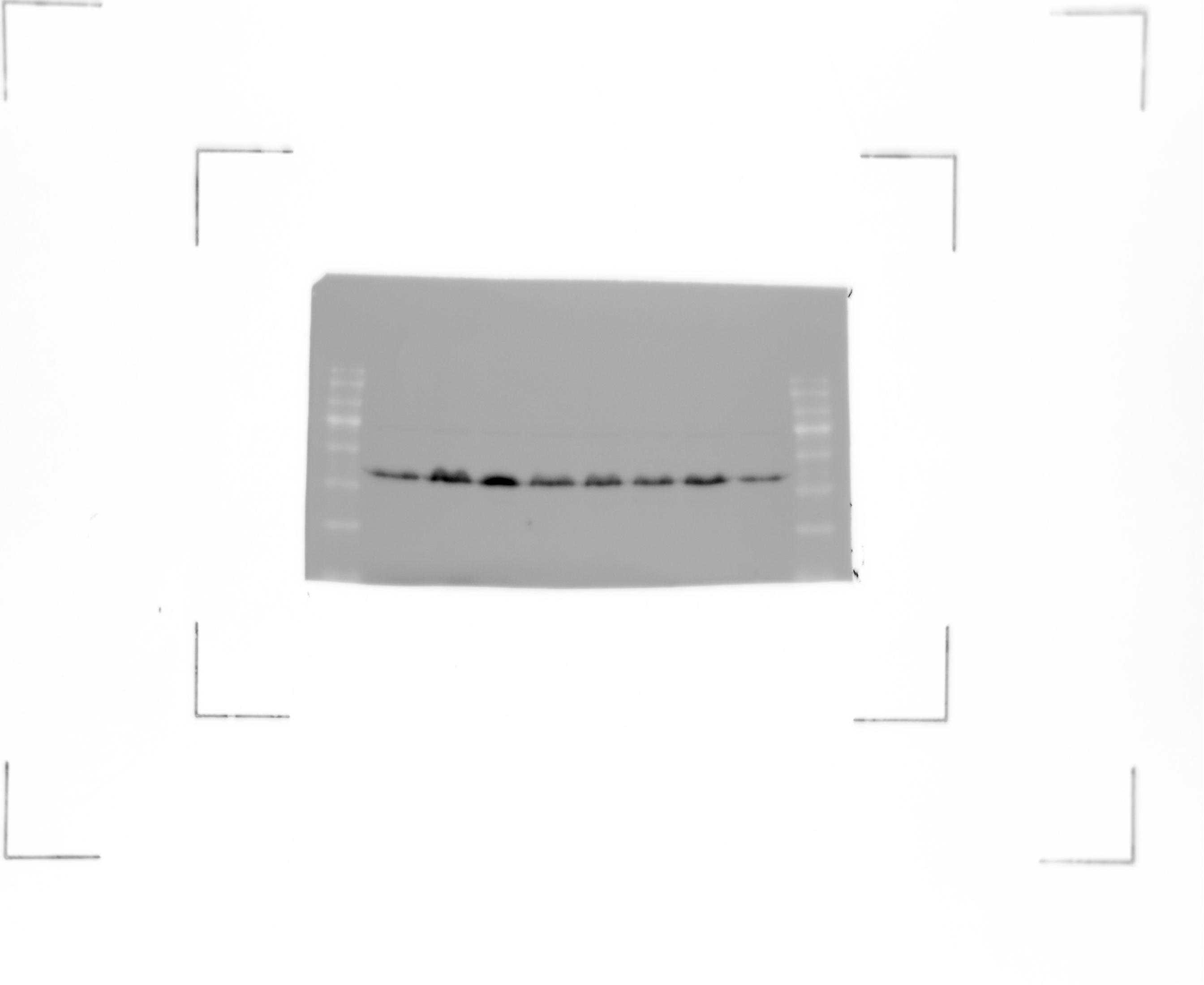

Supplement: Supplementary file 1 — Supplementary Material 1. [file 40001_2024_1968_MOESM1_ESM.zip › western blot original images/original images for all western blots/FIGURES1-3/Caspase 8-3.tif]

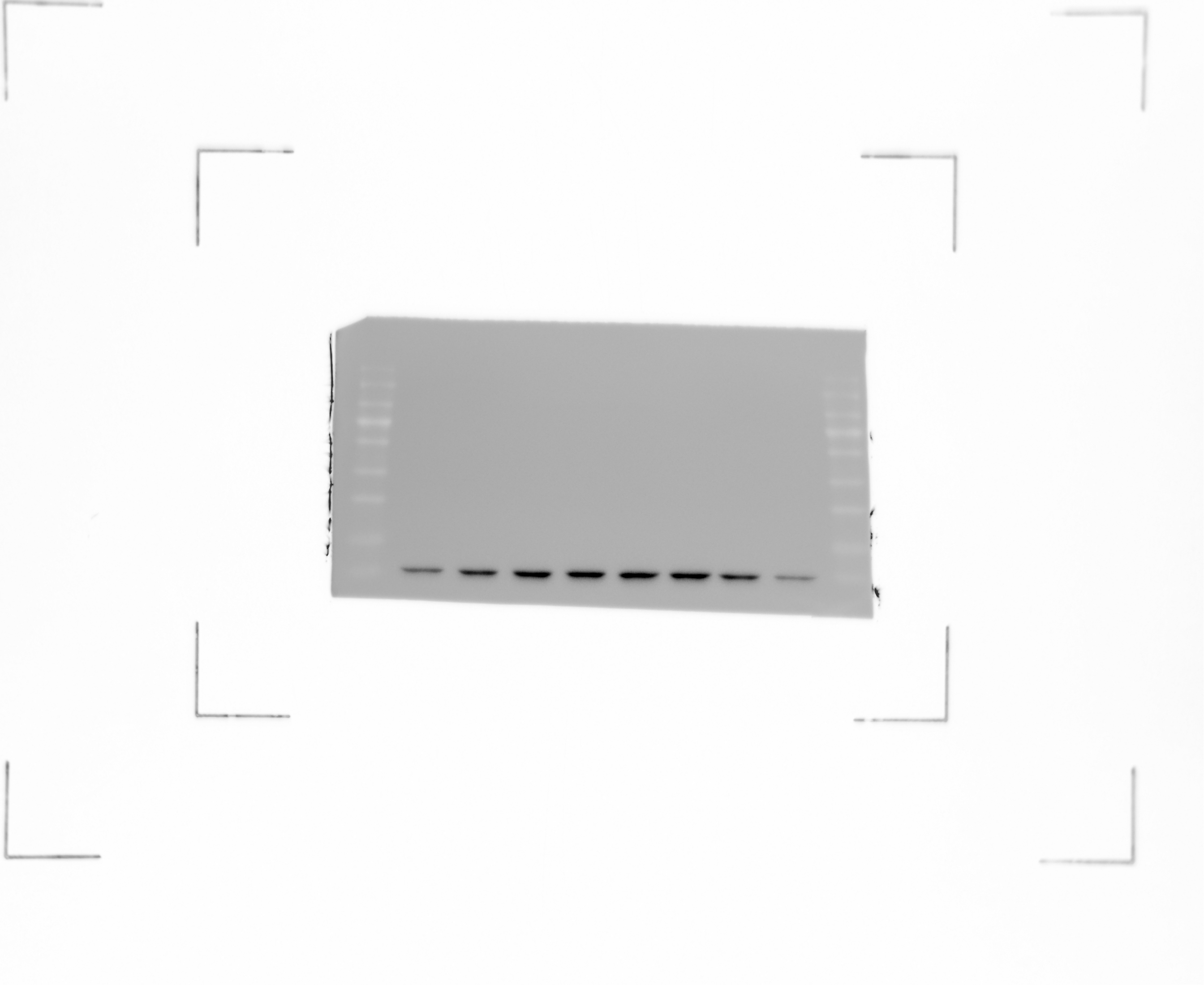

Supplement: Supplementary file 1 — Supplementary Material 1. [file 40001_2024_1968_MOESM1_ESM.zip › western blot original images/original images for all western blots/FIGURES1-3/Cleaved Caspase 3-1.tif]

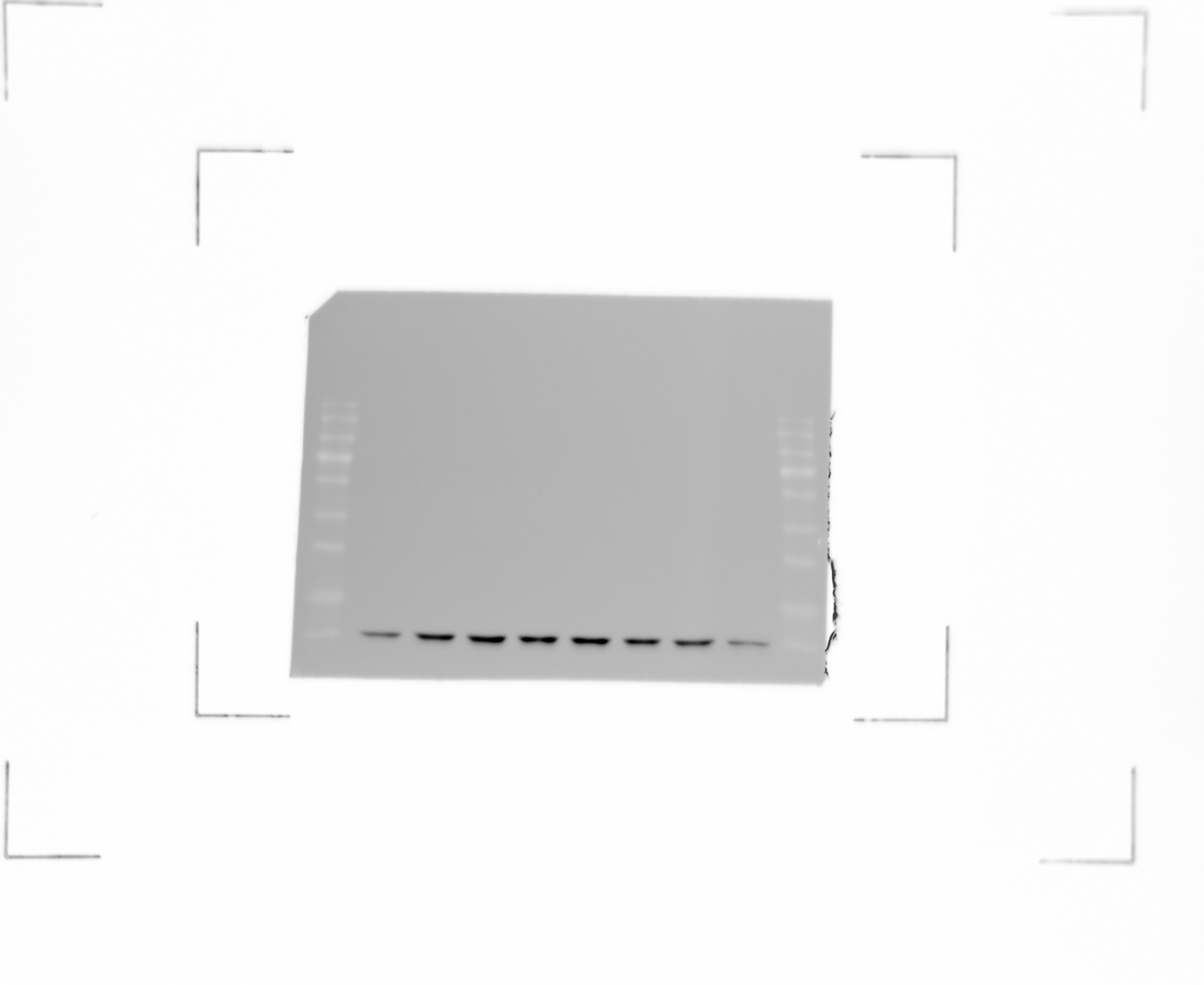

Supplement: Supplementary file 1 — Supplementary Material 1. [file 40001_2024_1968_MOESM1_ESM.zip › western blot original images/original images for all western blots/FIGURES1-3/Cleaved Caspase 3-2.tif]

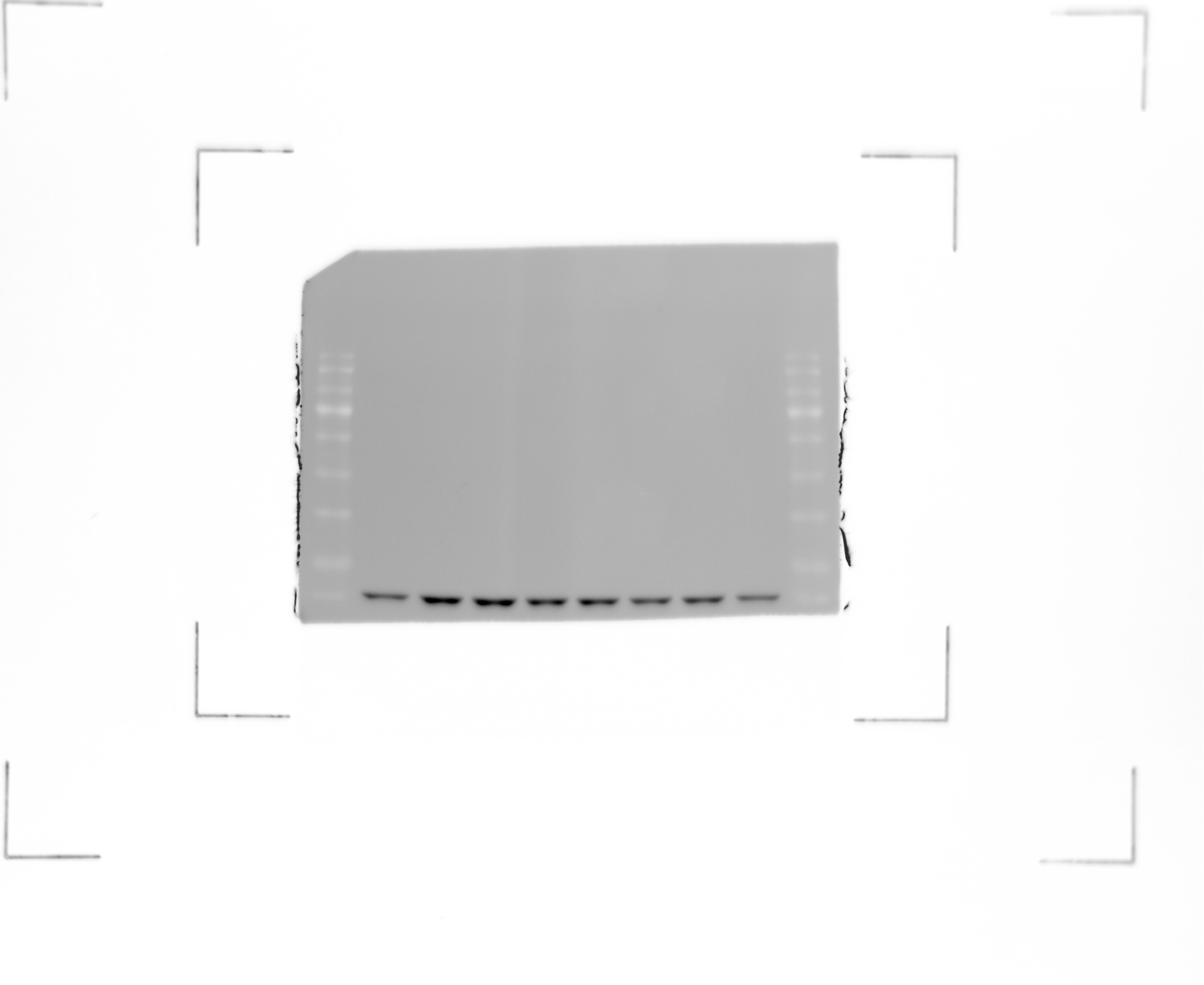

Supplement: Supplementary file 1 — Supplementary Material 1. [file 40001_2024_1968_MOESM1_ESM.zip › western blot original images/original images for all western blots/FIGURES1-3/Cleaved Caspase 3-3.tif]

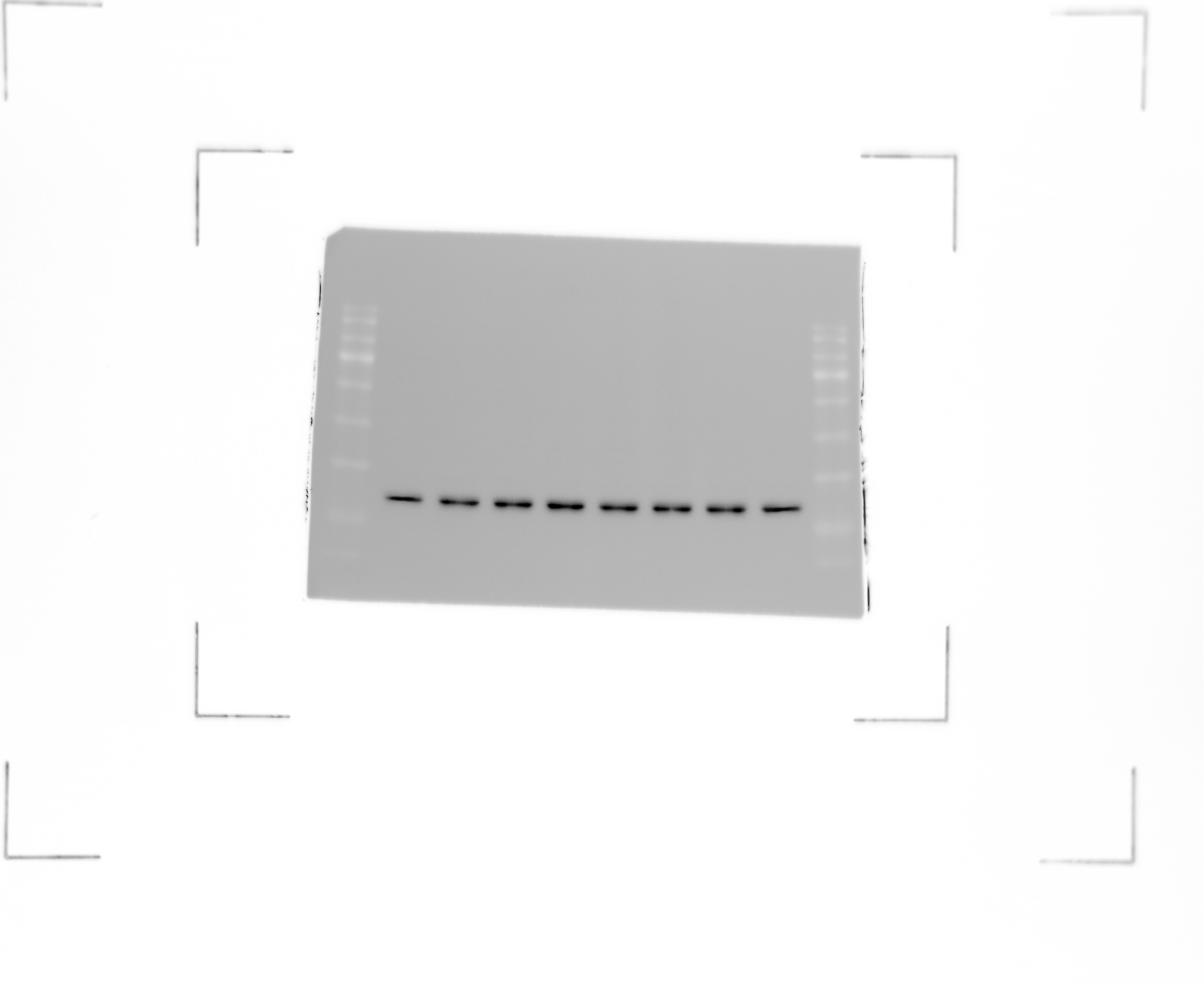

Supplement: Supplementary file 1 — Supplementary Material 1. [file 40001_2024_1968_MOESM1_ESM.zip › western blot original images/original images for all western blots/FIGURES1-3/FADD-1.tif]

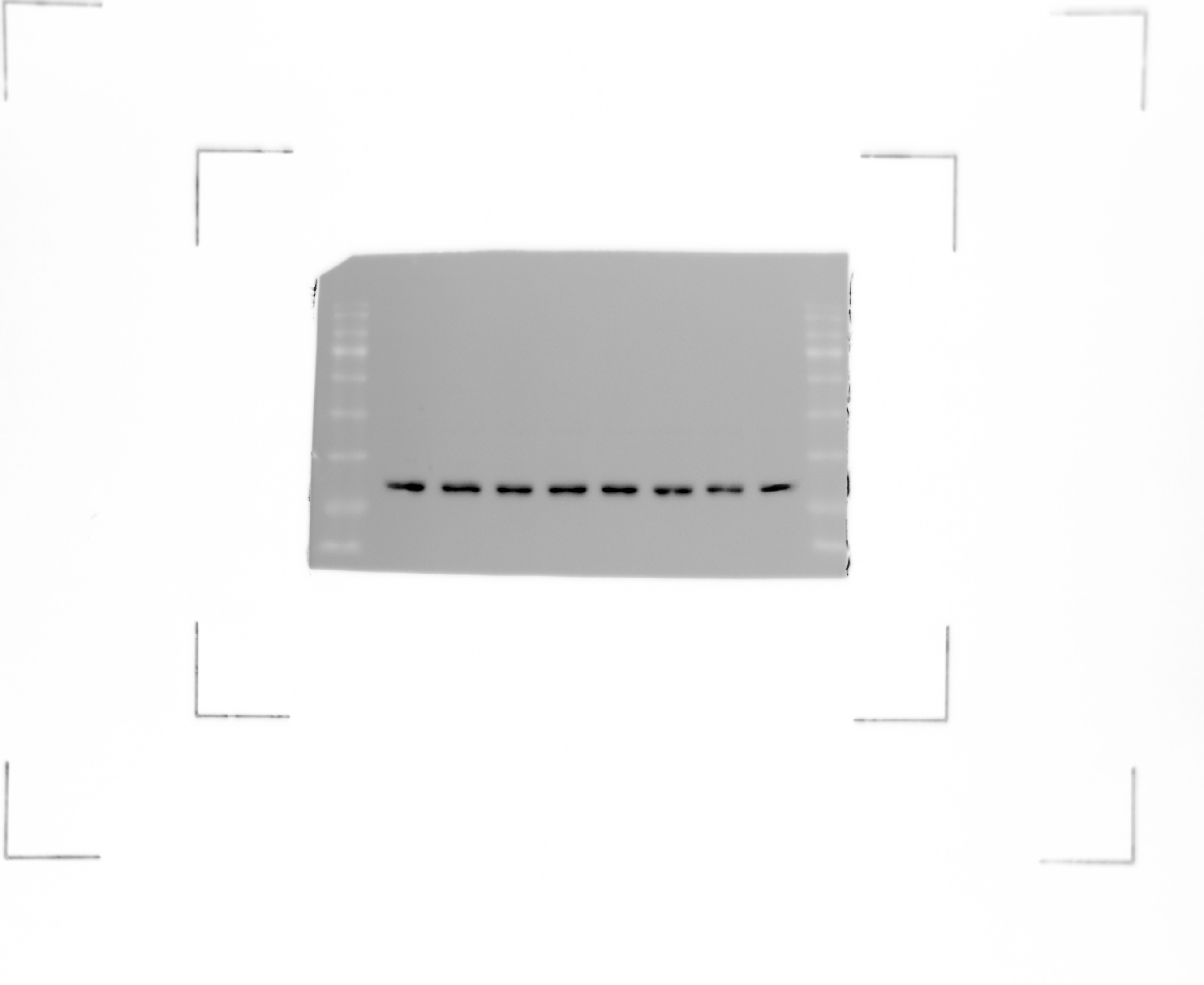

Supplement: Supplementary file 1 — Supplementary Material 1. [file 40001_2024_1968_MOESM1_ESM.zip › western blot original images/original images for all western blots/FIGURES1-3/FADD-2.tif]

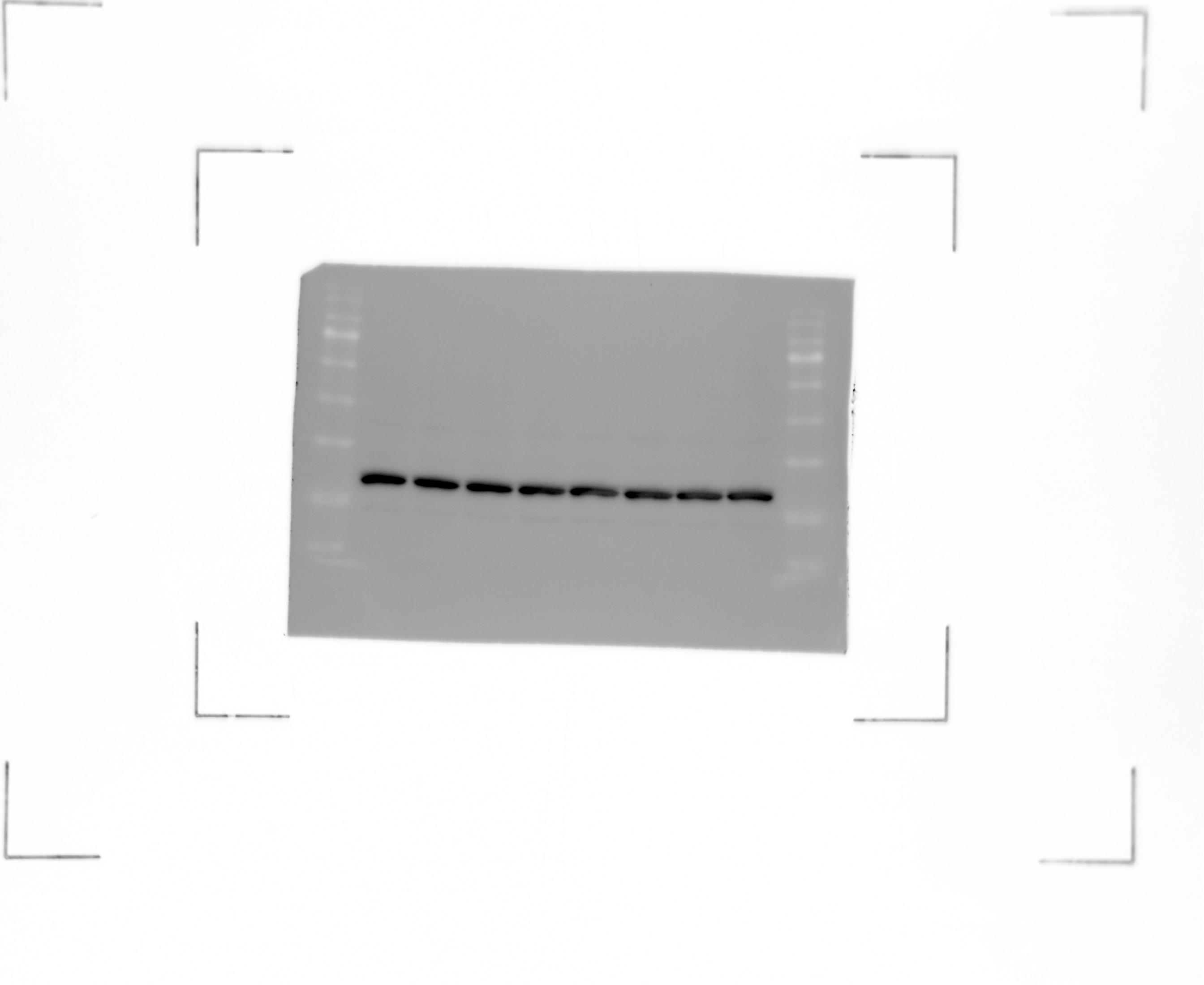

Supplement: Supplementary file 1 — Supplementary Material 1. [file 40001_2024_1968_MOESM1_ESM.zip › western blot original images/original images for all western blots/FIGURES1-3/FADD-3.tif]

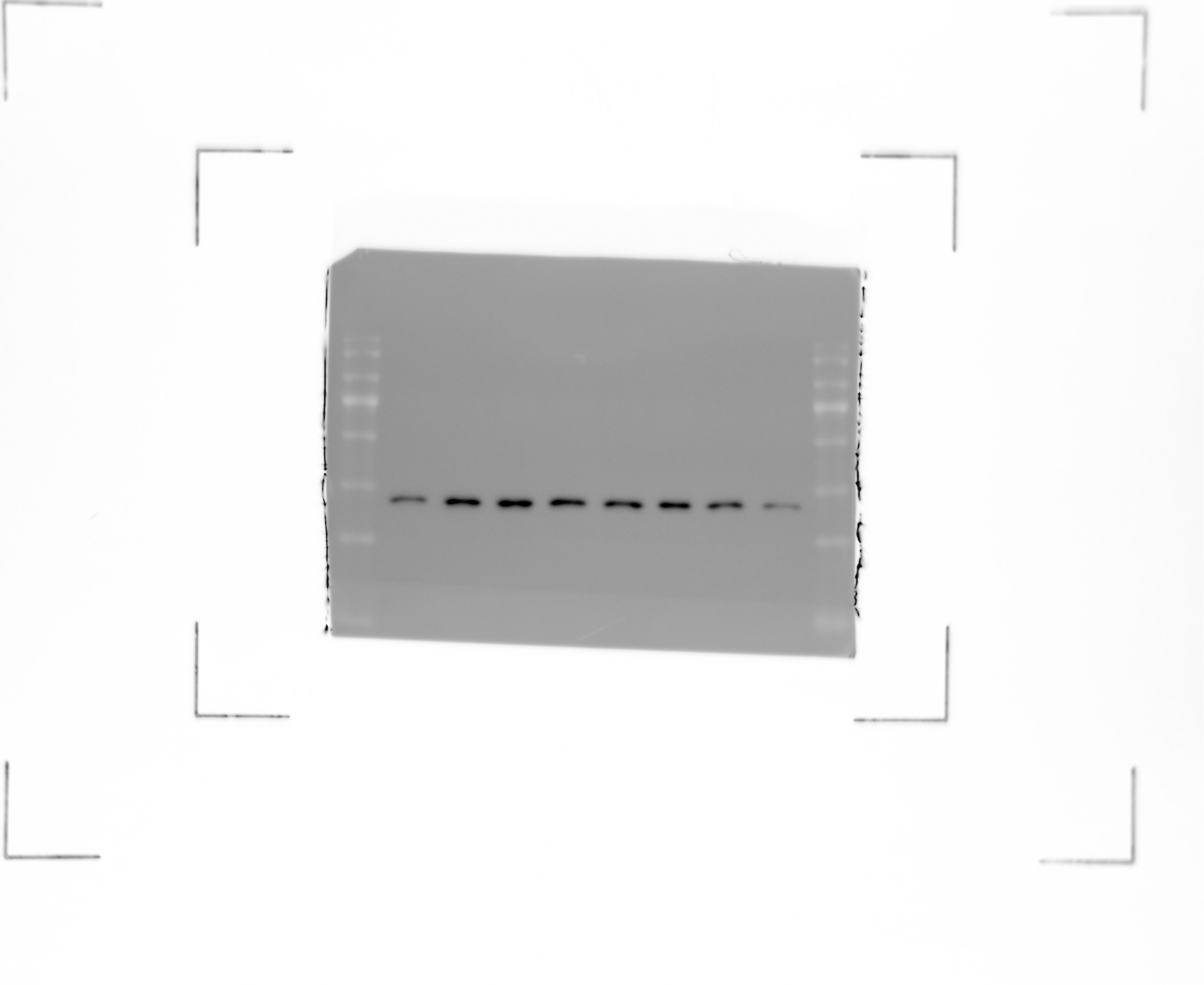

Supplement: Supplementary file 1 — Supplementary Material 1. [file 40001_2024_1968_MOESM1_ESM.zip › western blot original images/original images for all western blots/FIGURES1-3/FAS-1.tif]

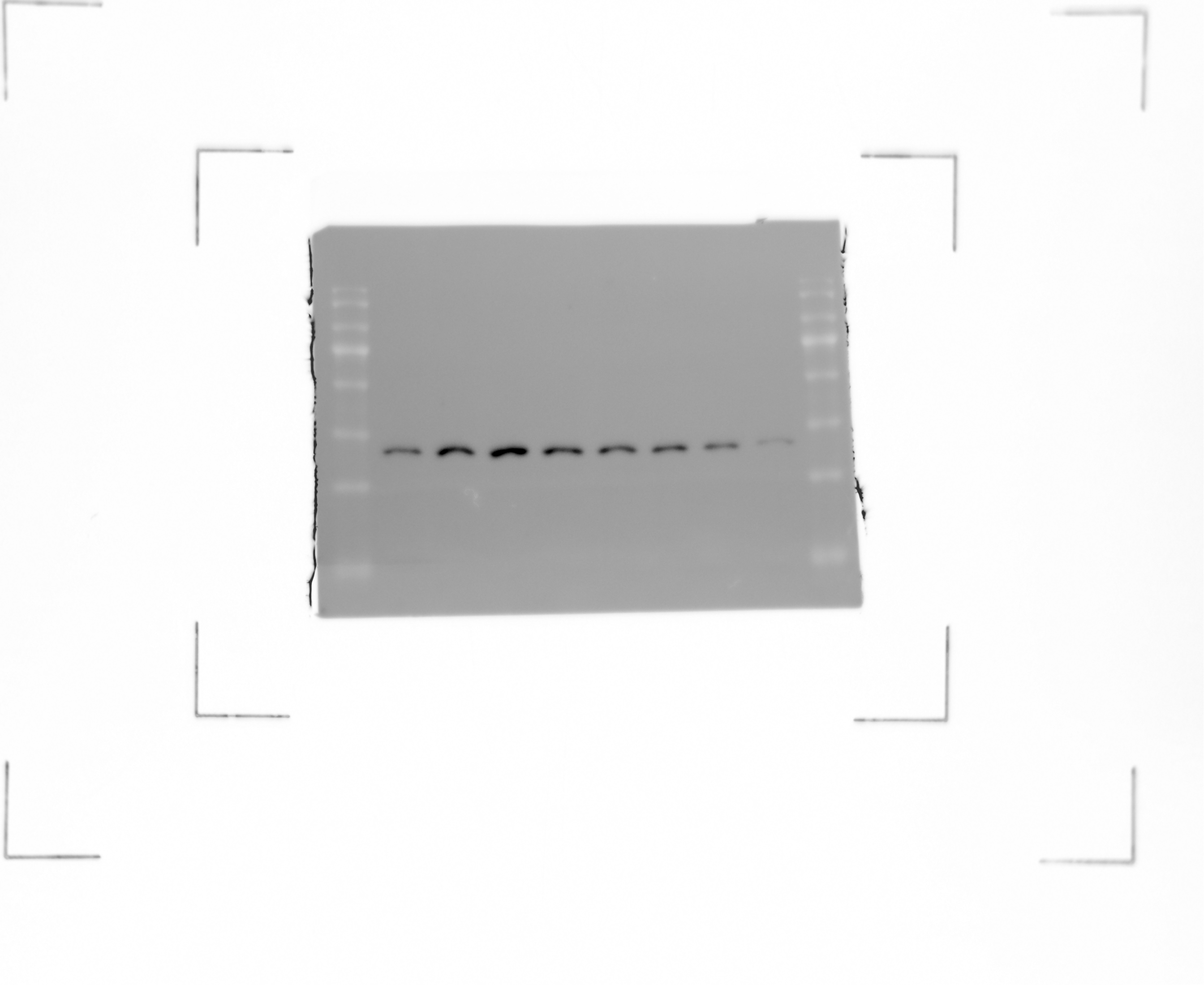

Supplement: Supplementary file 1 — Supplementary Material 1. [file 40001_2024_1968_MOESM1_ESM.zip › western blot original images/original images for all western blots/FIGURES1-3/FAS-2.tif]

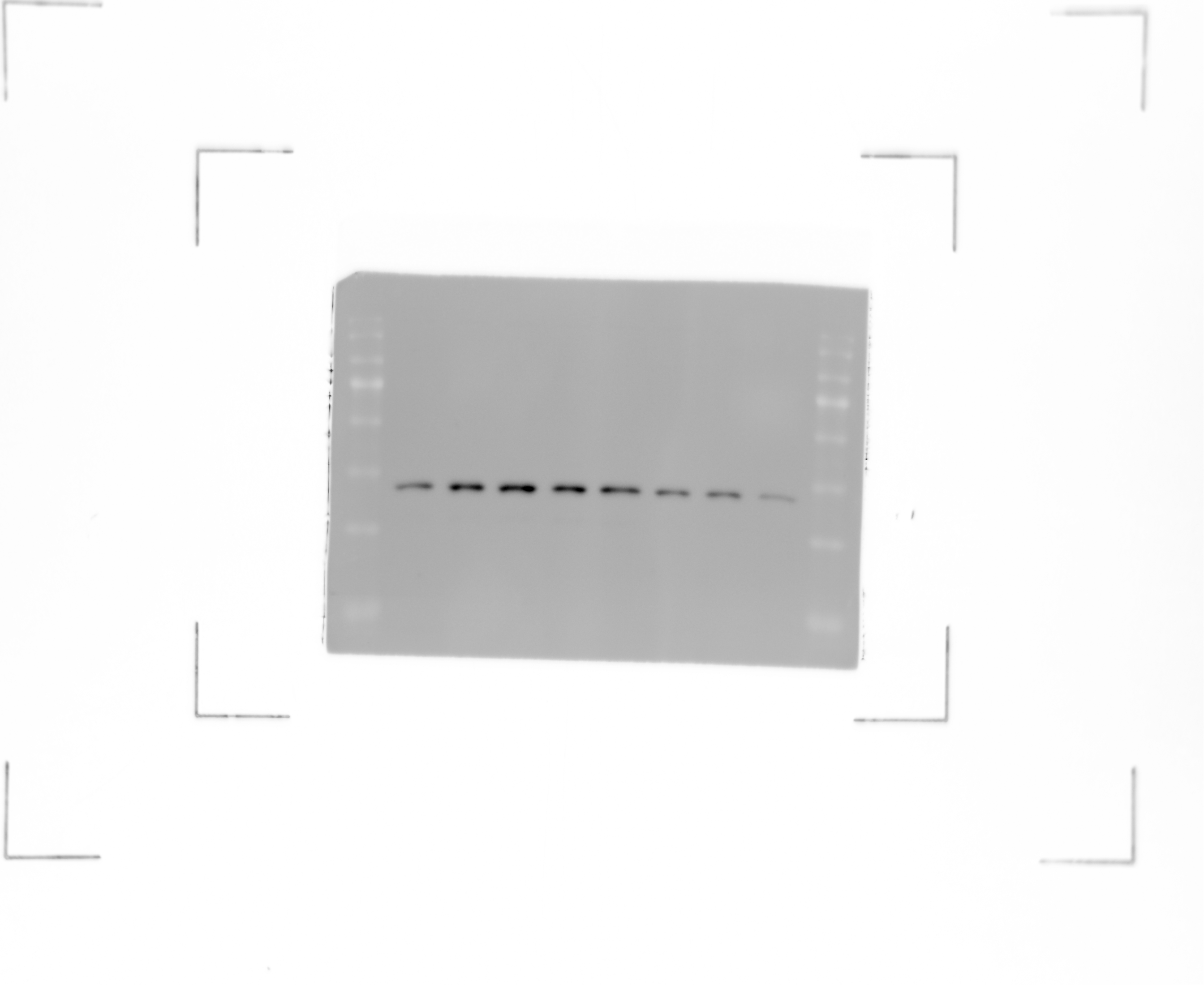

Supplement: Supplementary file 1 — Supplementary Material 1. [file 40001_2024_1968_MOESM1_ESM.zip › western blot original images/original images for all western blots/FIGURES1-3/FAS-3.tif]

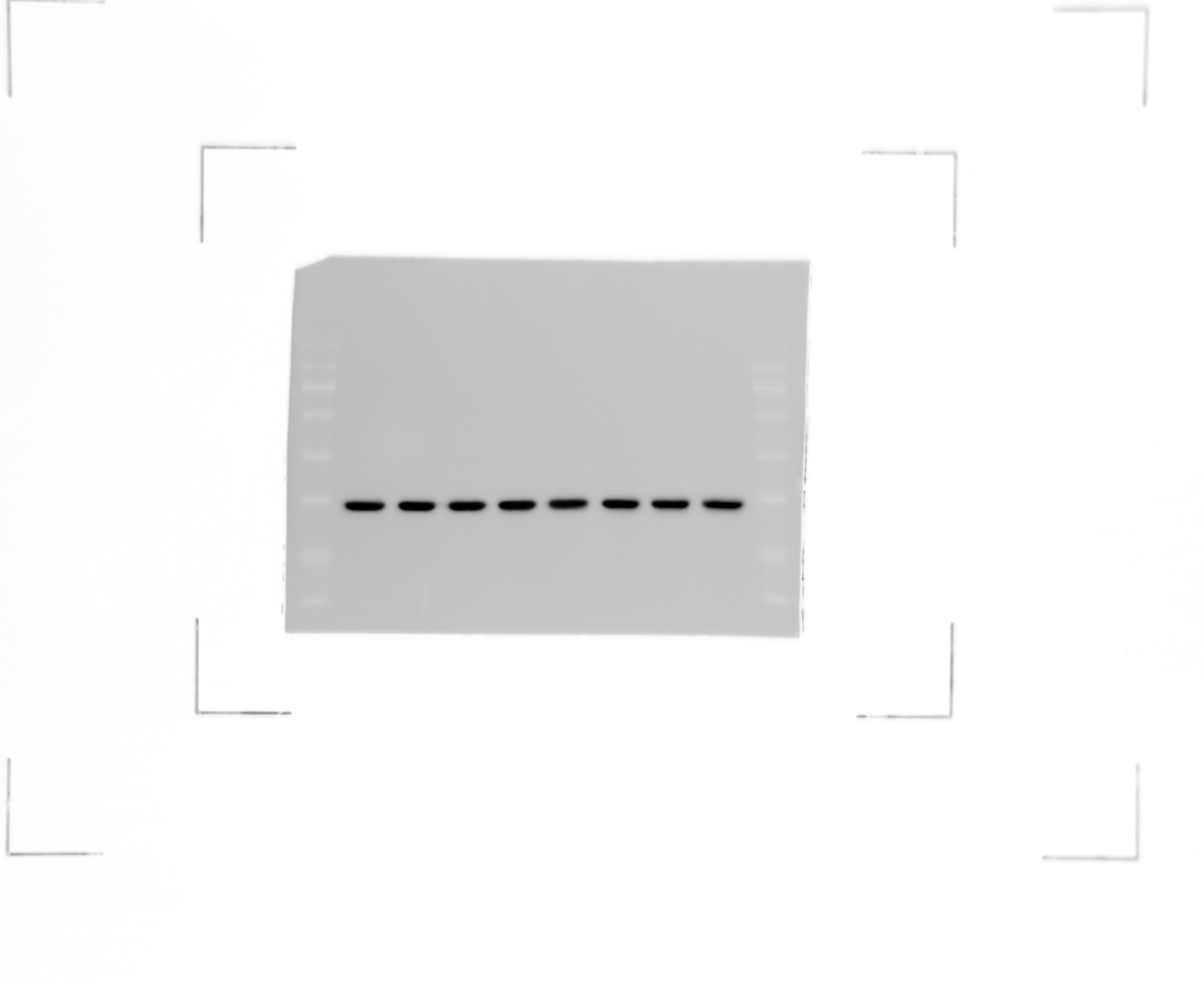

Supplement: Supplementary file 1 — Supplementary Material 1. [file 40001_2024_1968_MOESM1_ESM.zip › western blot original images/original images for all western blots/FIGURES1-3/GAPDH-1.tif]

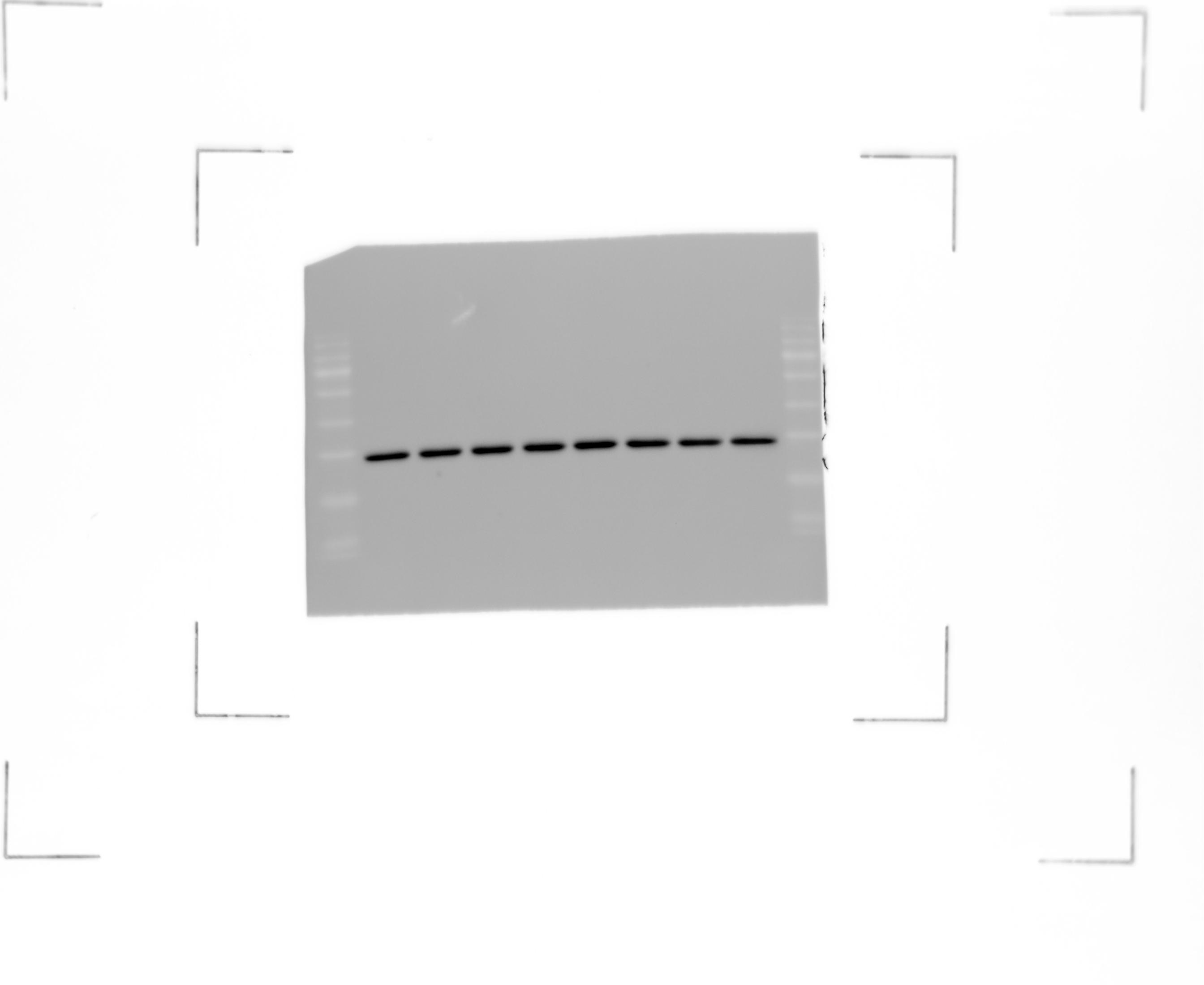

Supplement: Supplementary file 1 — Supplementary Material 1. [file 40001_2024_1968_MOESM1_ESM.zip › western blot original images/original images for all western blots/FIGURES1-3/GAPDH-2.tif]

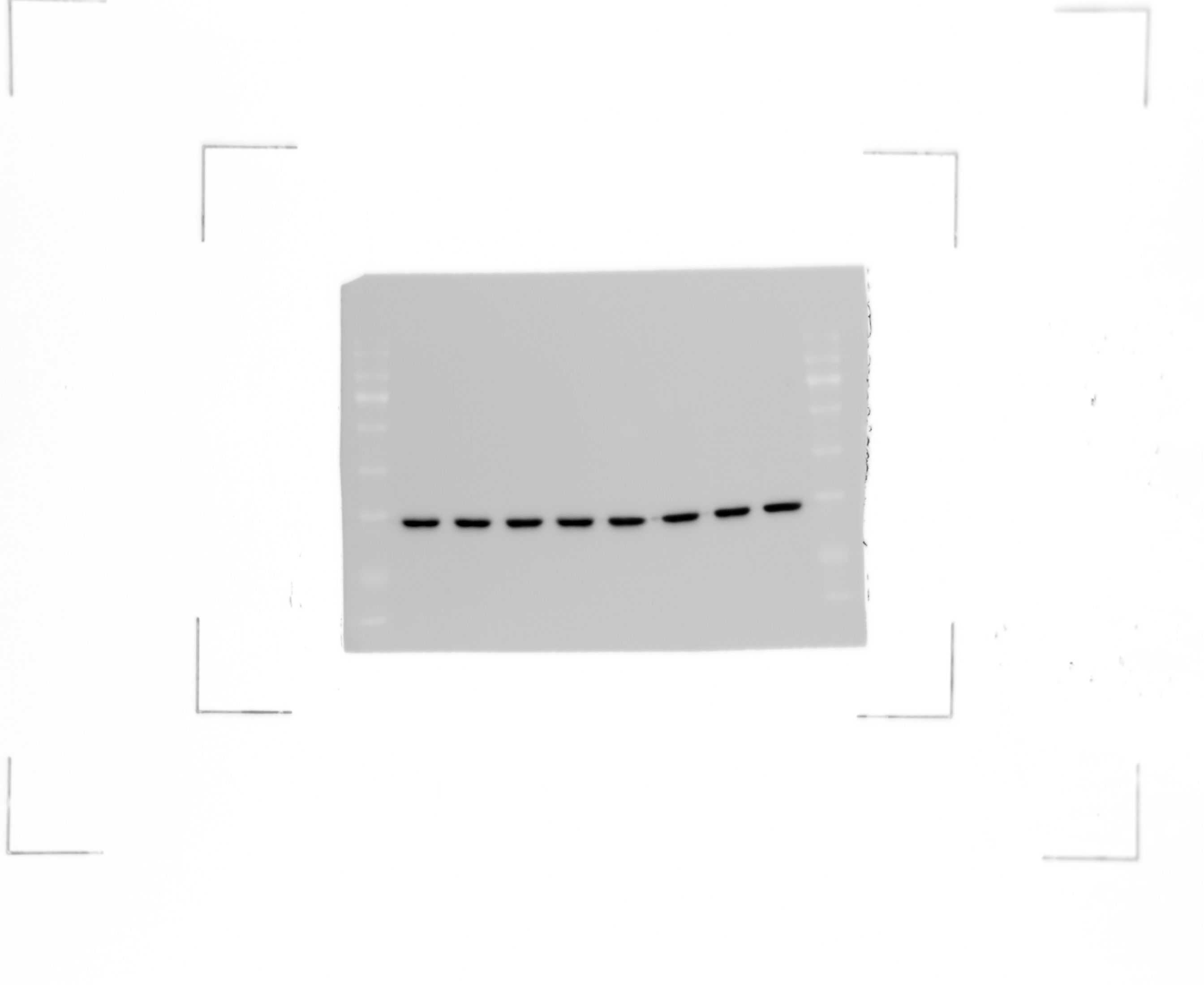

Supplement: Supplementary file 1 — Supplementary Material 1. [file 40001_2024_1968_MOESM1_ESM.zip › western blot original images/original images for all western blots/FIGURES1-3/GAPDH-3.tif]

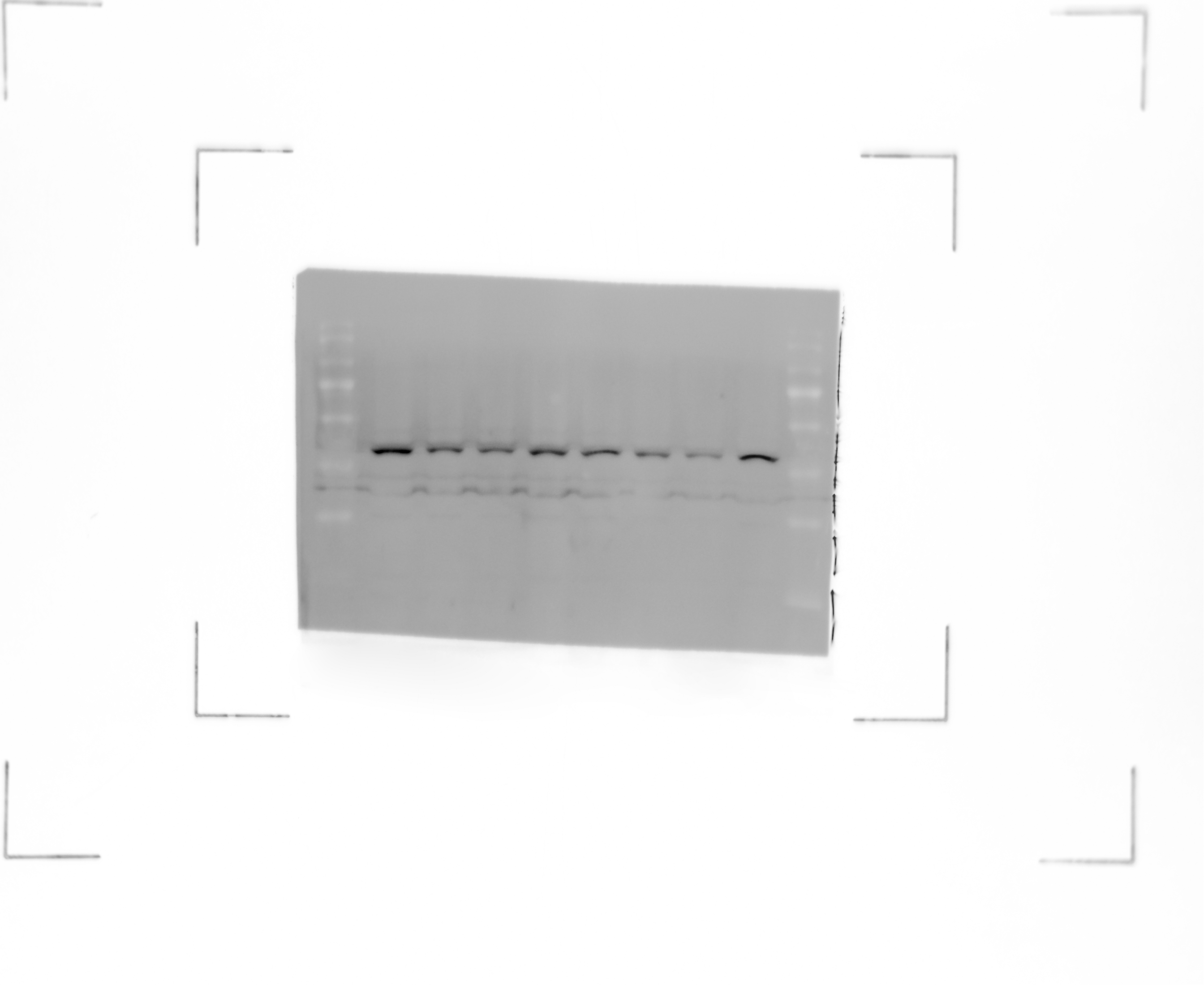

Supplement: Supplementary file 1 — Supplementary Material 1. [file 40001_2024_1968_MOESM1_ESM.zip › western blot original images/original images for all western blots/FIGURES1-3/KRT8-1.tif]

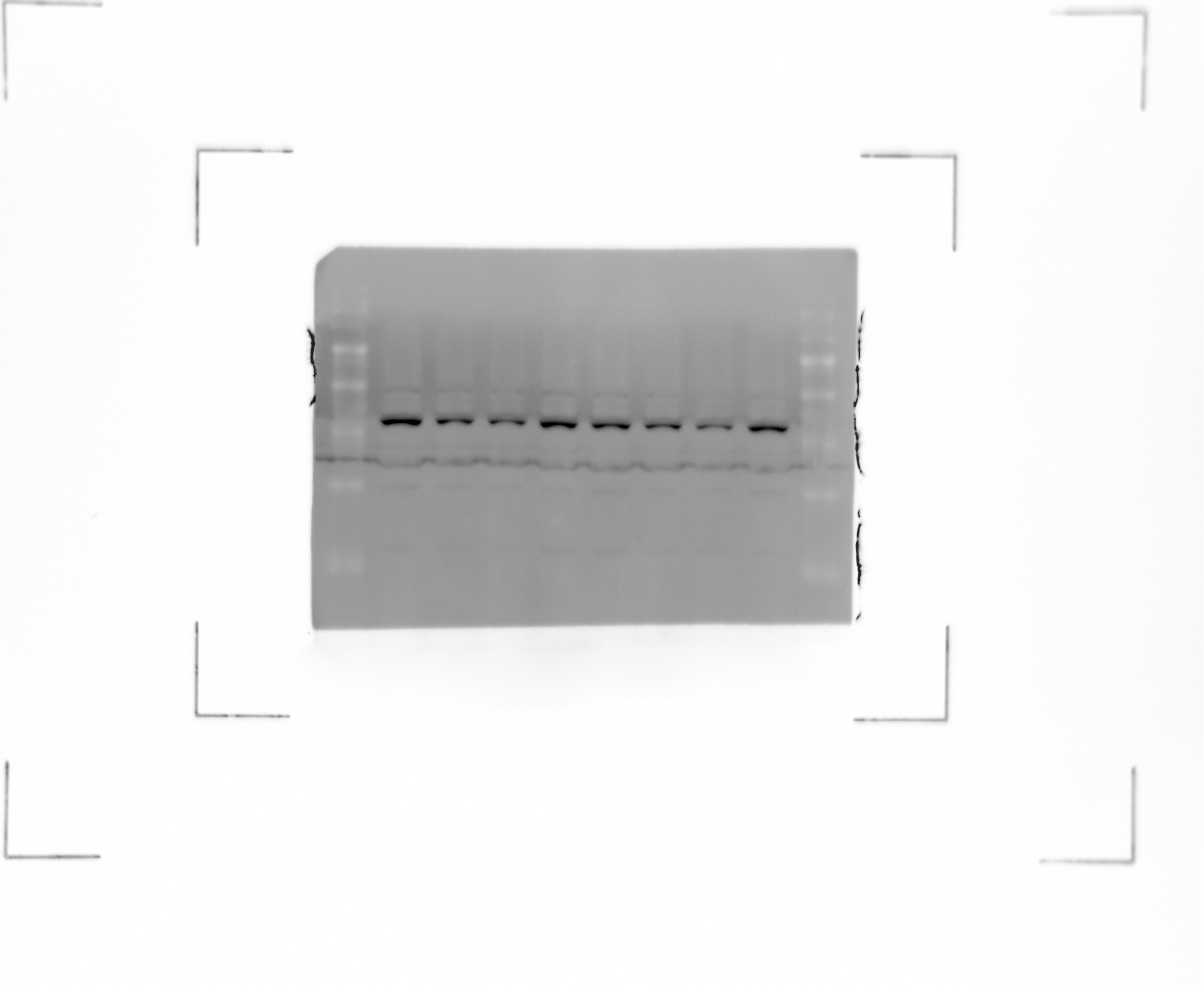

Supplement: Supplementary file 1 — Supplementary Material 1. [file 40001_2024_1968_MOESM1_ESM.zip › western blot original images/original images for all western blots/FIGURES1-3/KRT8-2.tif]

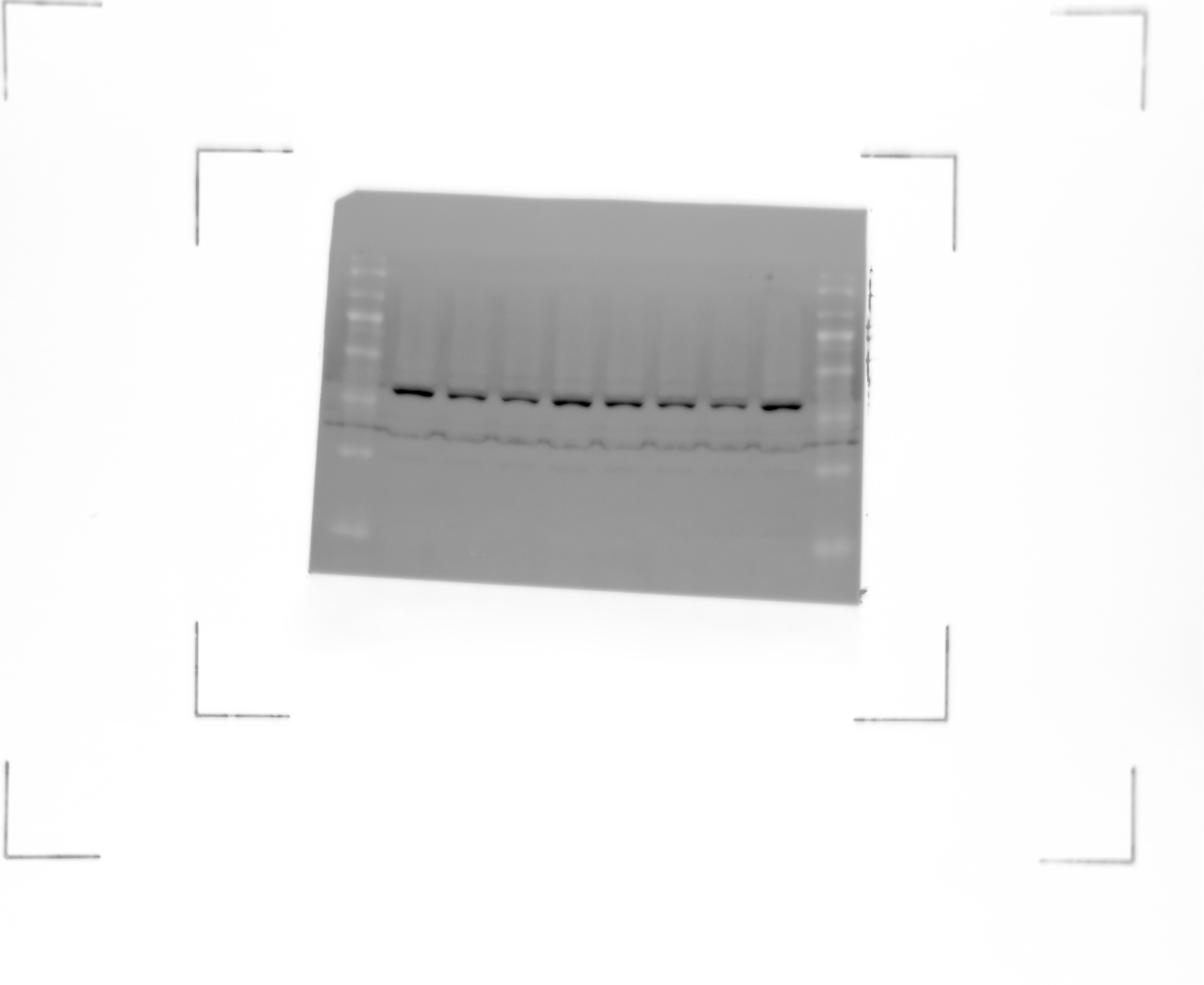

Supplement: Supplementary file 1 — Supplementary Material 1. [file 40001_2024_1968_MOESM1_ESM.zip › western blot original images/original images for all western blots/FIGURES1-3/KRT8-3.tif]

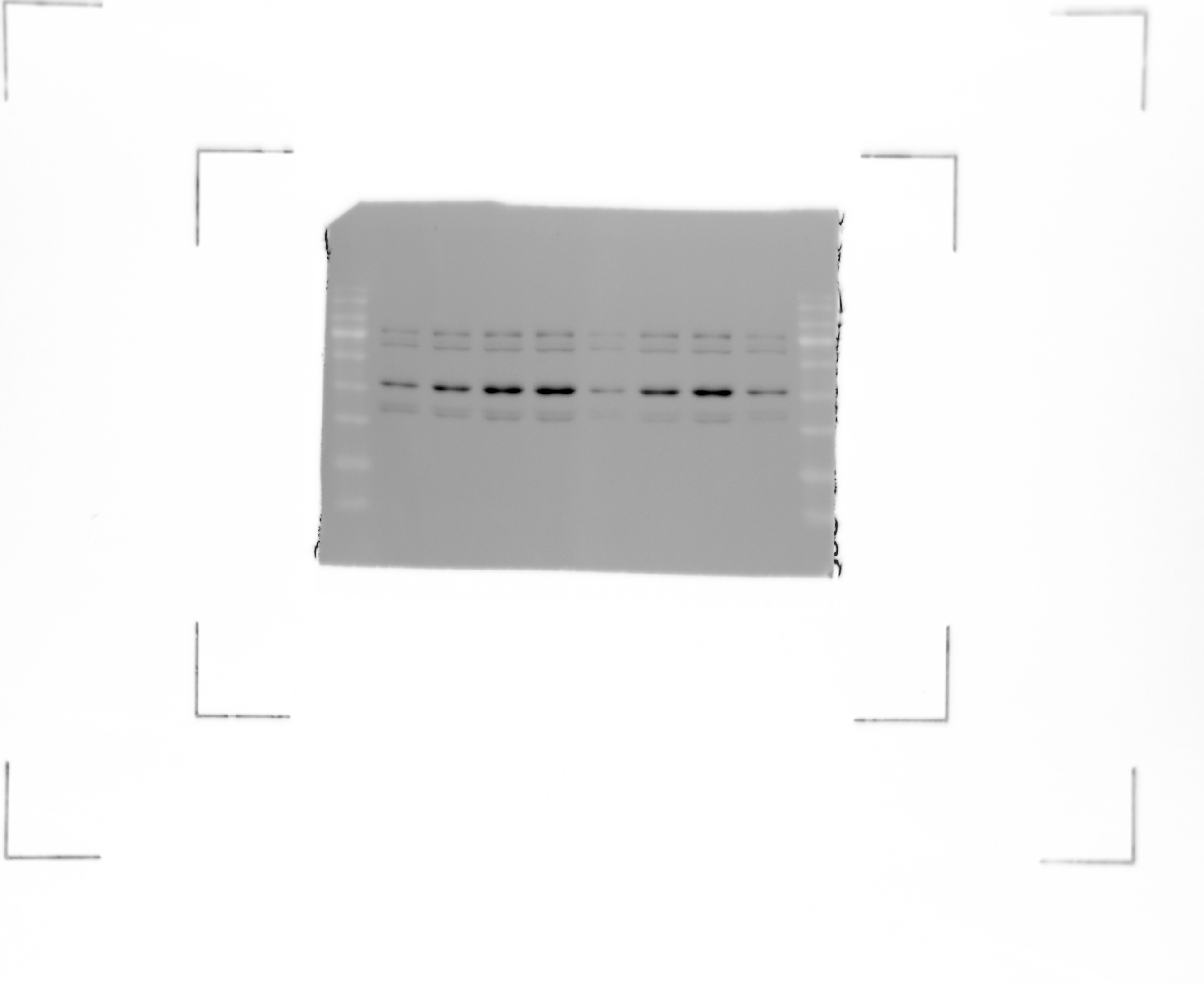

Supplement: Supplementary file 1 — Supplementary Material 1. [file 40001_2024_1968_MOESM1_ESM.zip › western blot original images/original images for all western blots/FIGURES1-3/METTL14-1.tif]

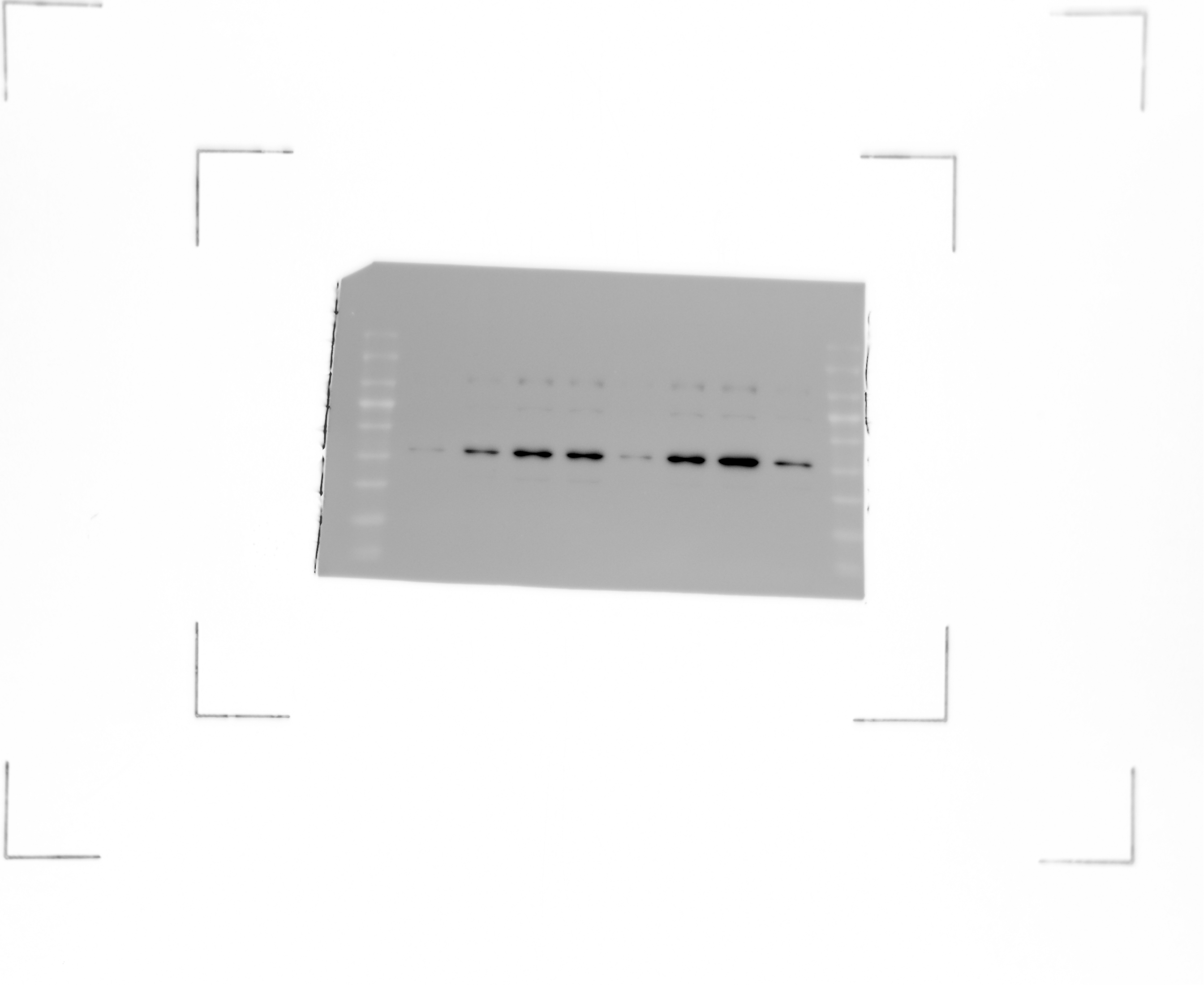

Supplement: Supplementary file 1 — Supplementary Material 1. [file 40001_2024_1968_MOESM1_ESM.zip › western blot original images/original images for all western blots/FIGURES1-3/METTL14-2.tif]

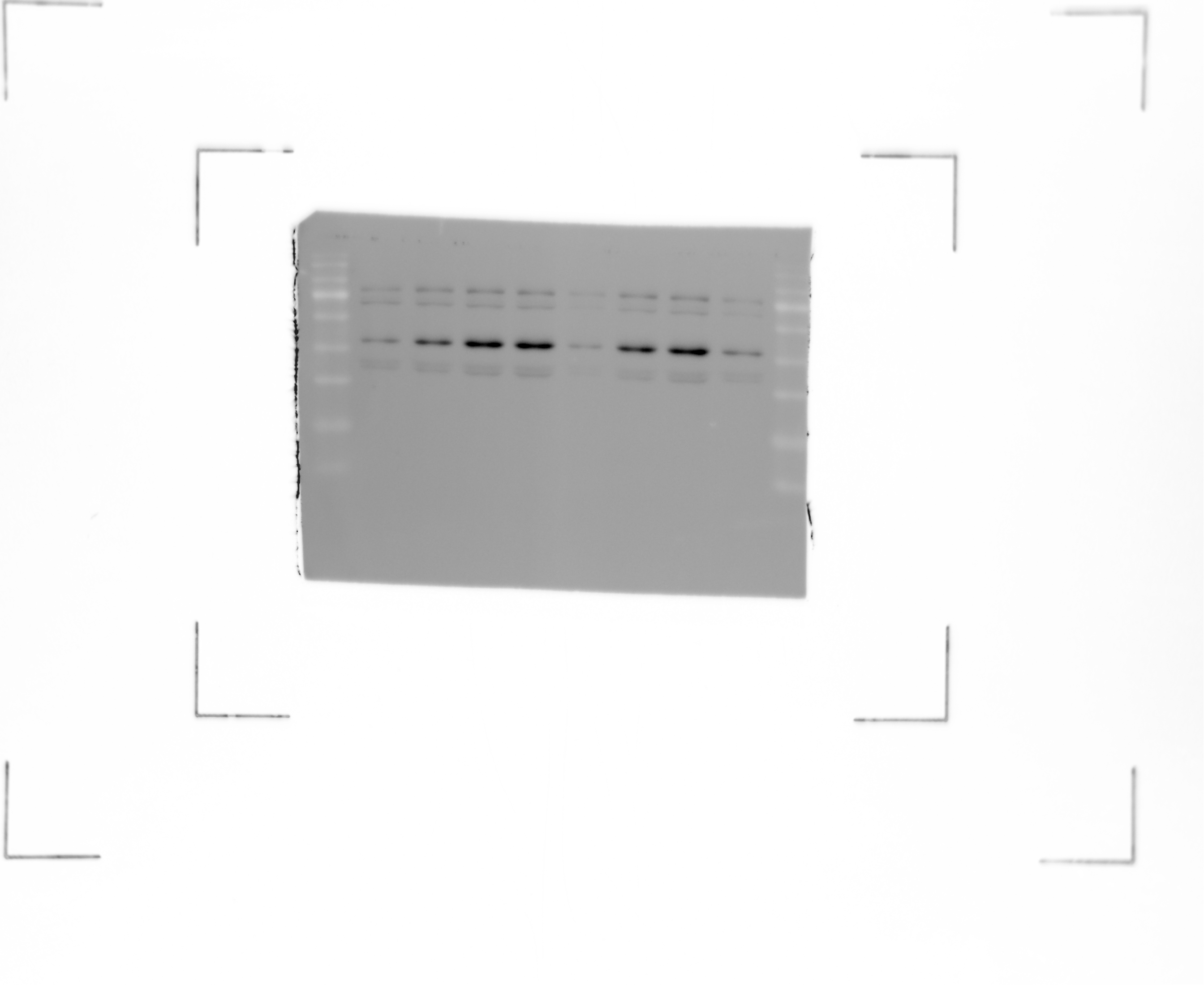

Supplement: Supplementary file 1 — Supplementary Material 1. [file 40001_2024_1968_MOESM1_ESM.zip › western blot original images/original images for all western blots/FIGURES1-3/METTL14-3.tif]

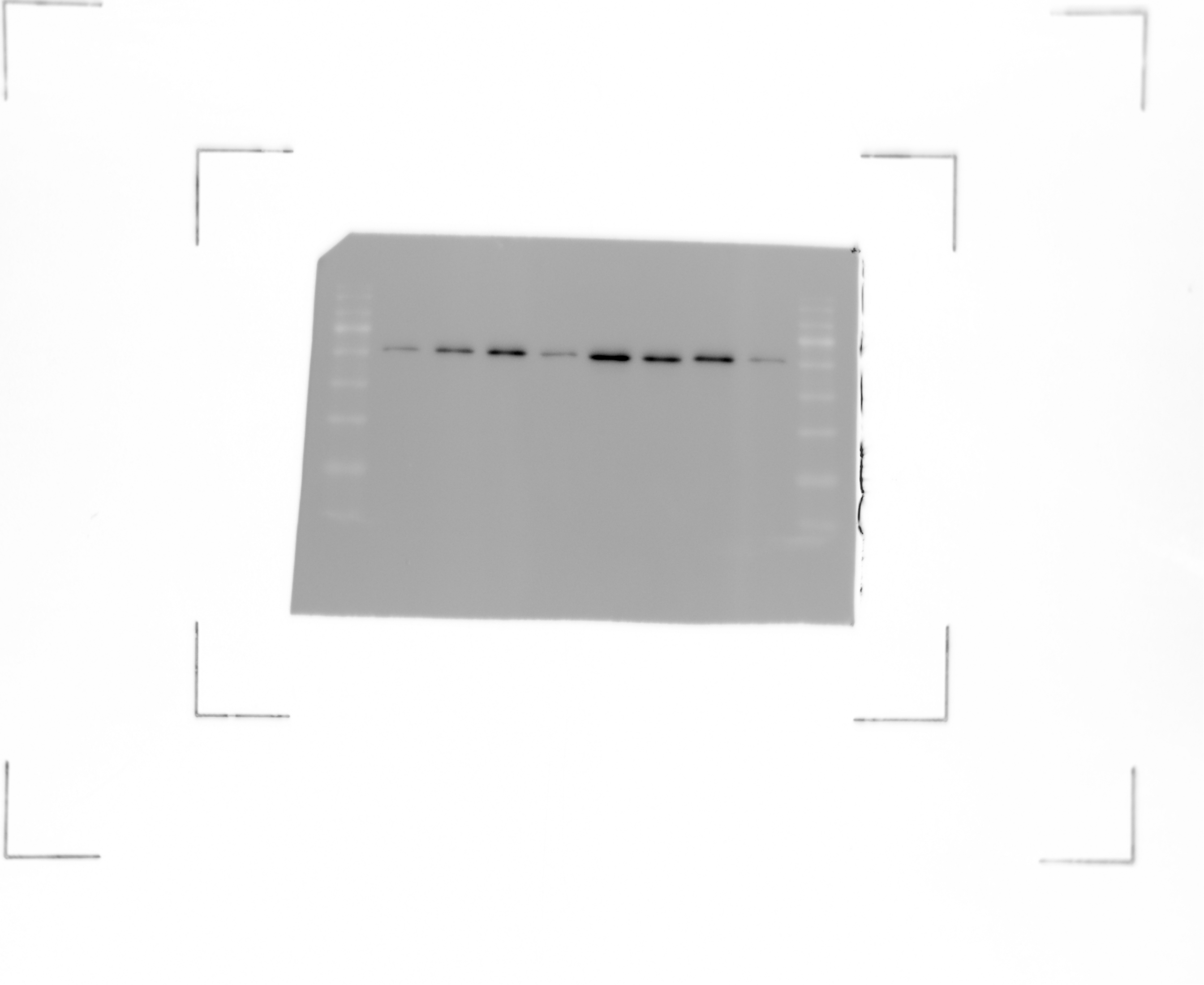

Supplement: Supplementary file 1 — Supplementary Material 1. [file 40001_2024_1968_MOESM1_ESM.zip › western blot original images/original images for all western blots/FIGURES1-3/METTL3-1.tif]

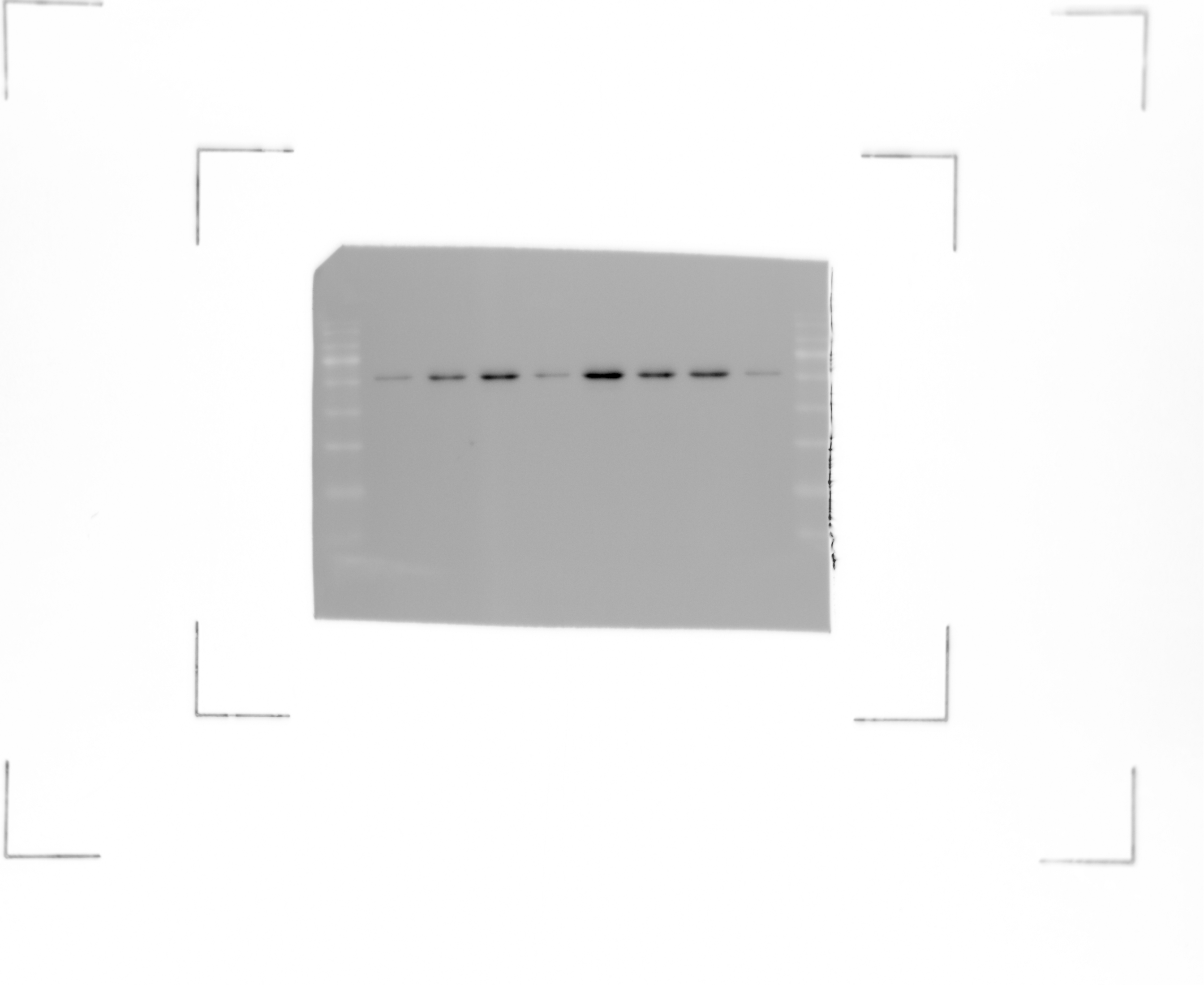

Supplement: Supplementary file 1 — Supplementary Material 1. [file 40001_2024_1968_MOESM1_ESM.zip › western blot original images/original images for all western blots/FIGURES1-3/METTL3-2.tif]

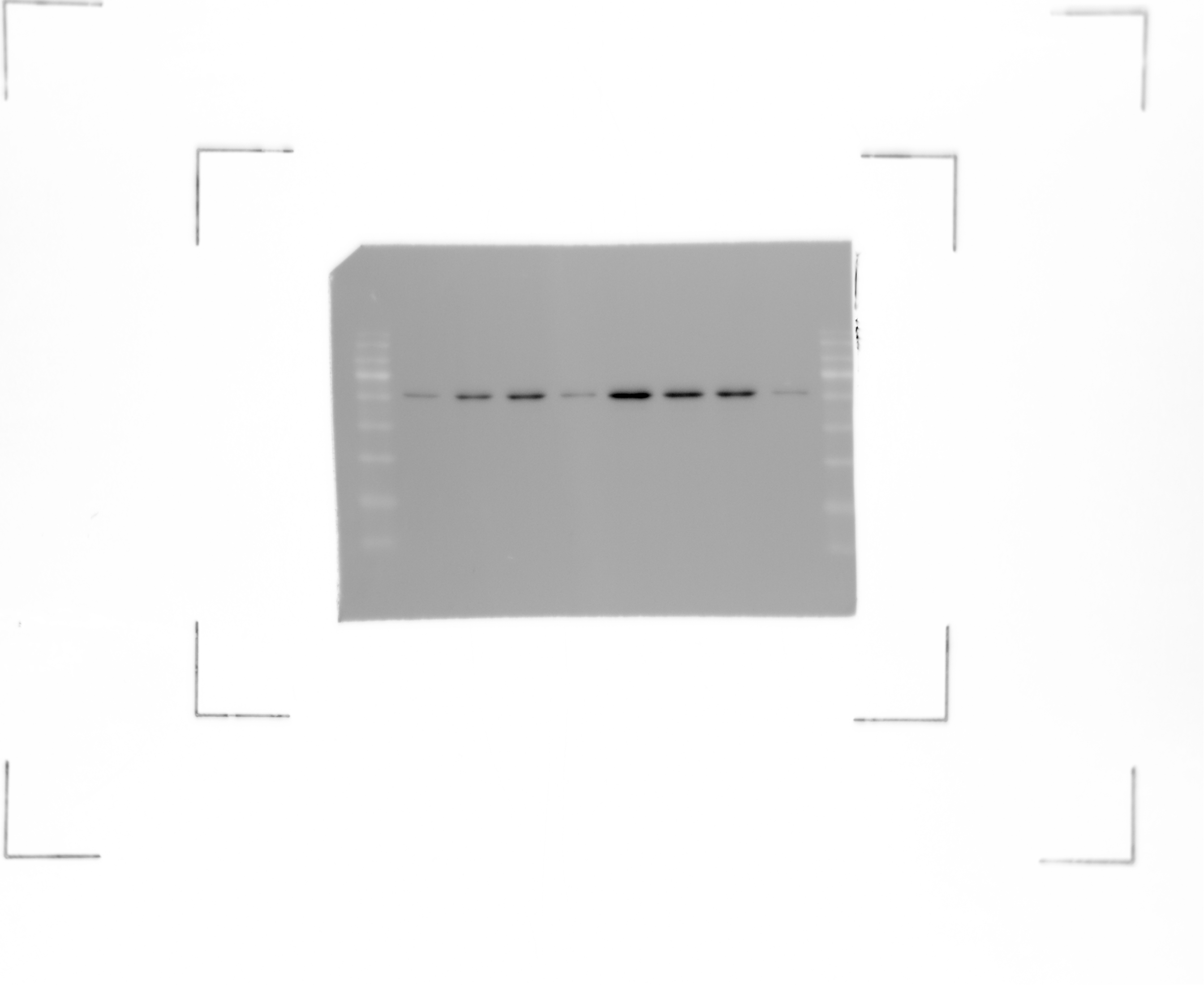

Supplement: Supplementary file 1 — Supplementary Material 1. [file 40001_2024_1968_MOESM1_ESM.zip › western blot original images/original images for all western blots/FIGURES1-3/METTL3-3.tif]

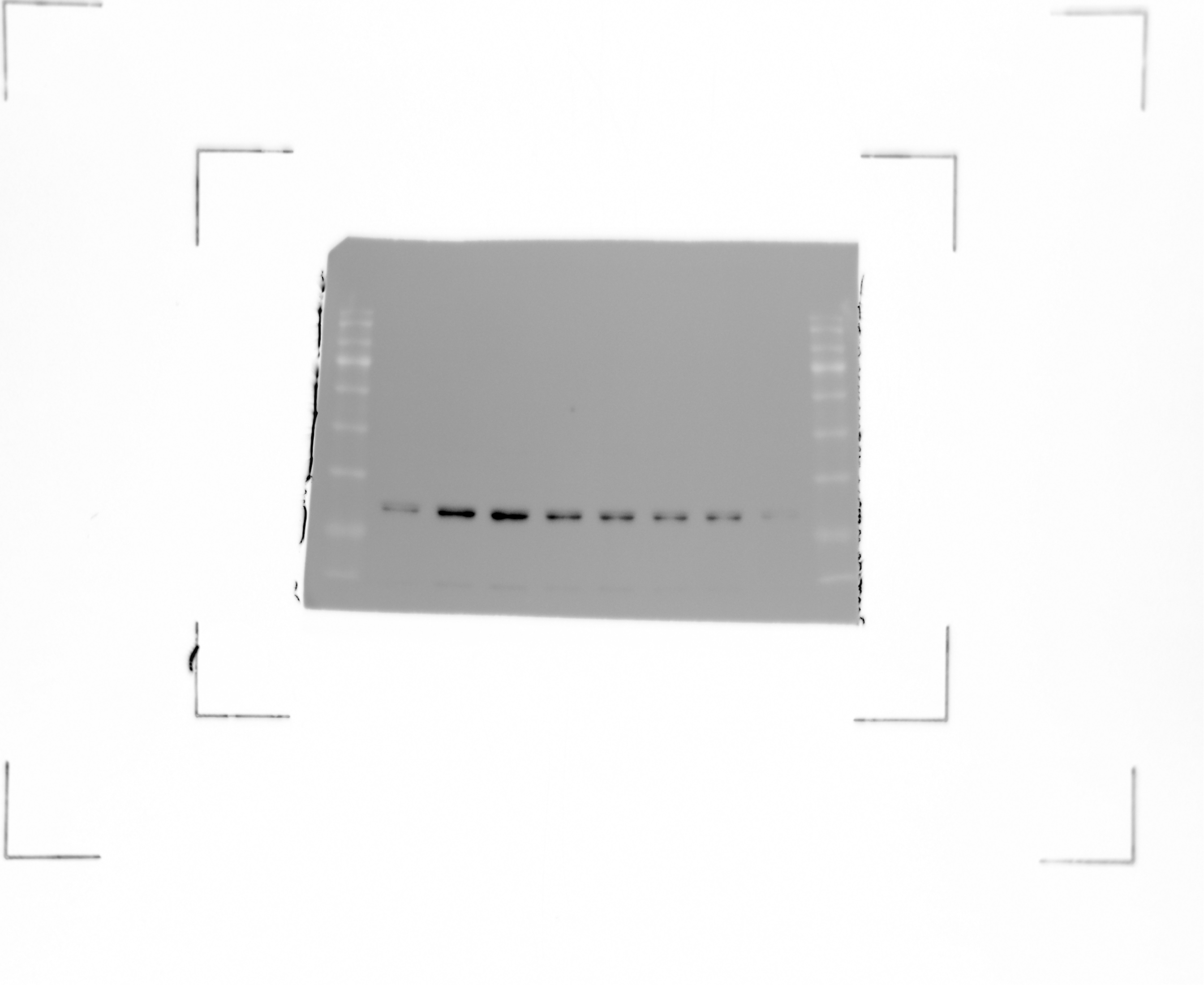

Supplement: Supplementary file 1 — Supplementary Material 1. [file 40001_2024_1968_MOESM1_ESM.zip › western blot original images/original images for all western blots/FIGURES1-3/p-FADD-1.tif]

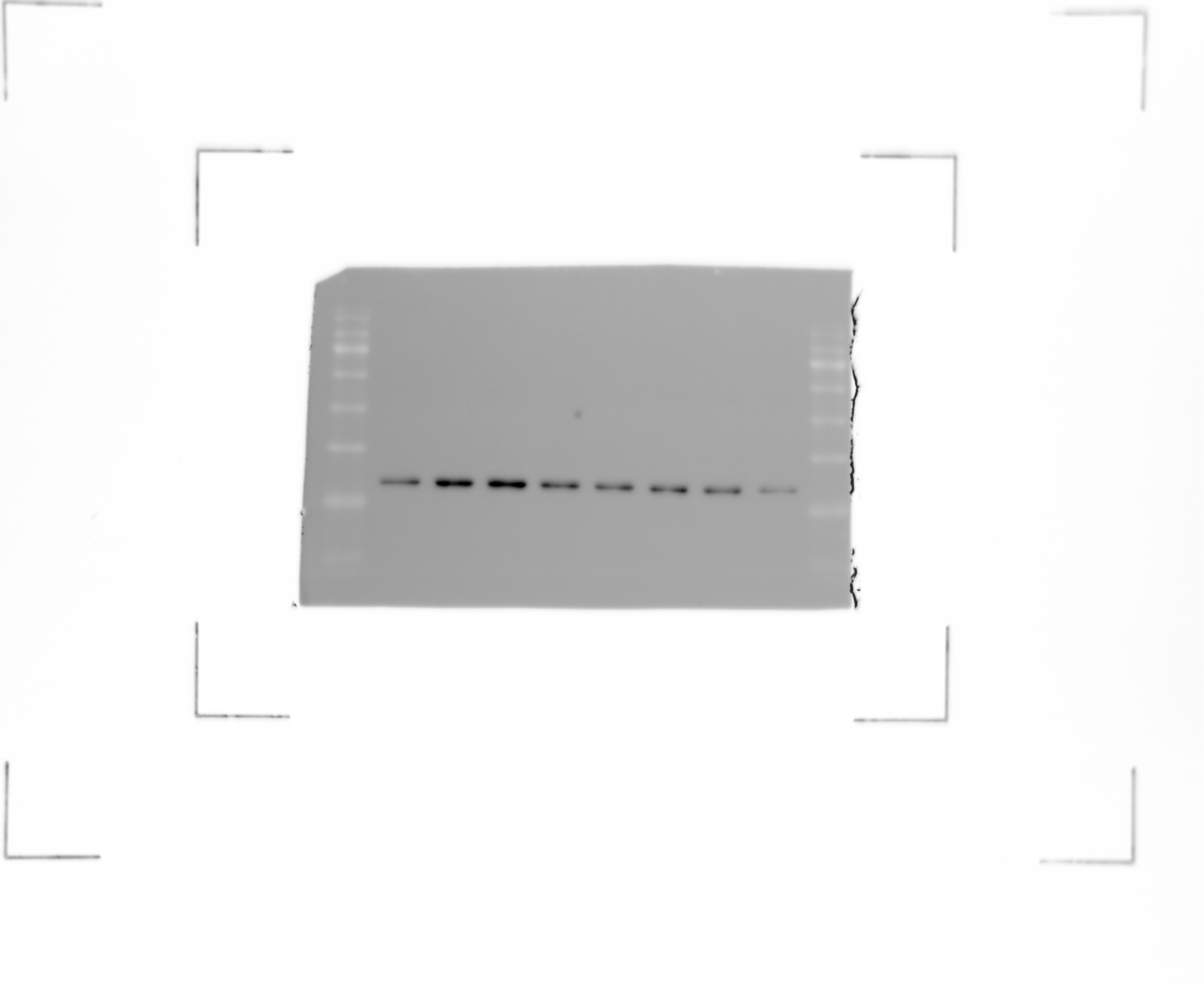

Supplement: Supplementary file 1 — Supplementary Material 1. [file 40001_2024_1968_MOESM1_ESM.zip › western blot original images/original images for all western blots/FIGURES1-3/p-FADD-2.tif]

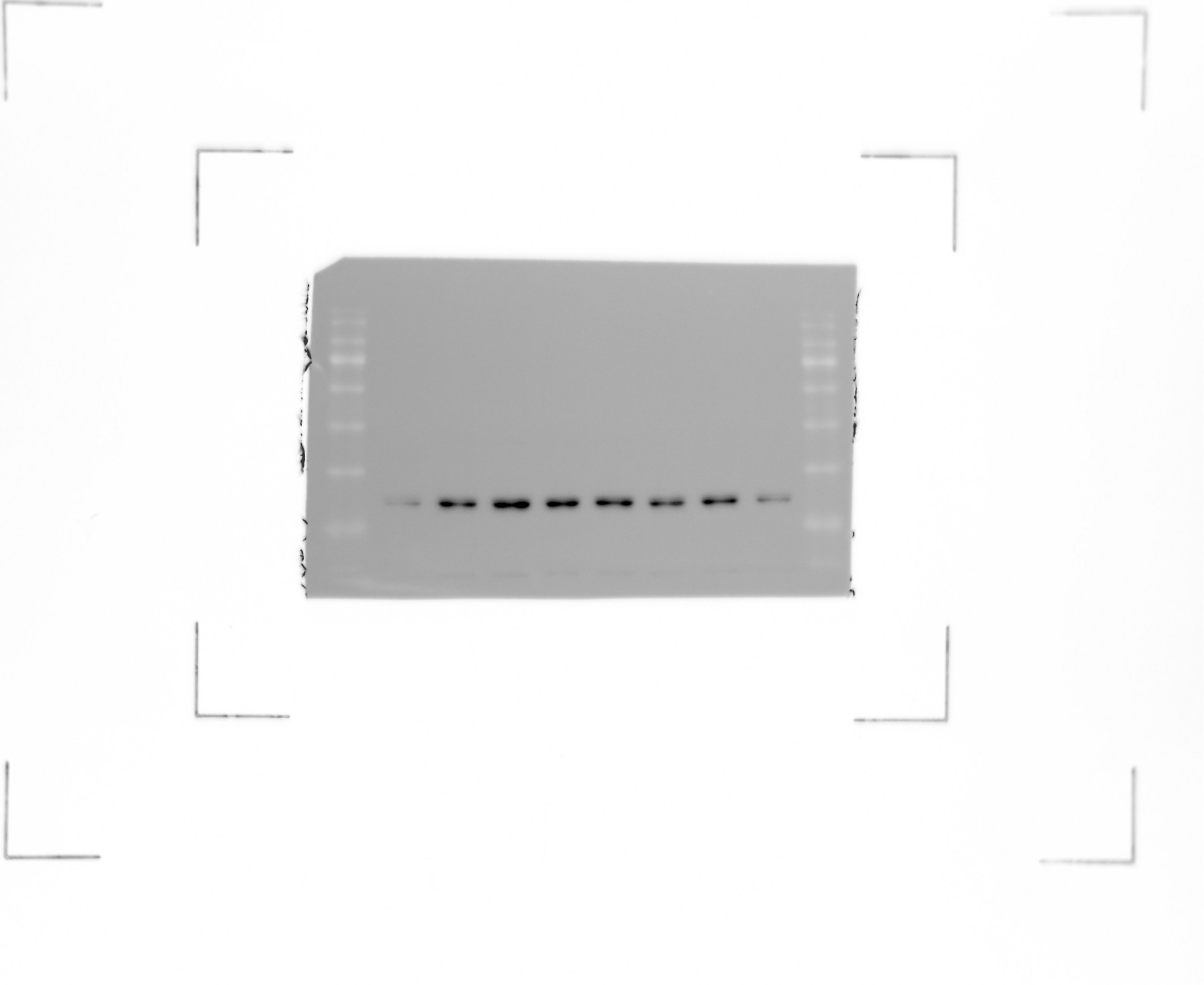

Supplement: Supplementary file 1 — Supplementary Material 1. [file 40001_2024_1968_MOESM1_ESM.zip › western blot original images/original images for all western blots/FIGURES1-3/p-FADD-3.tif]

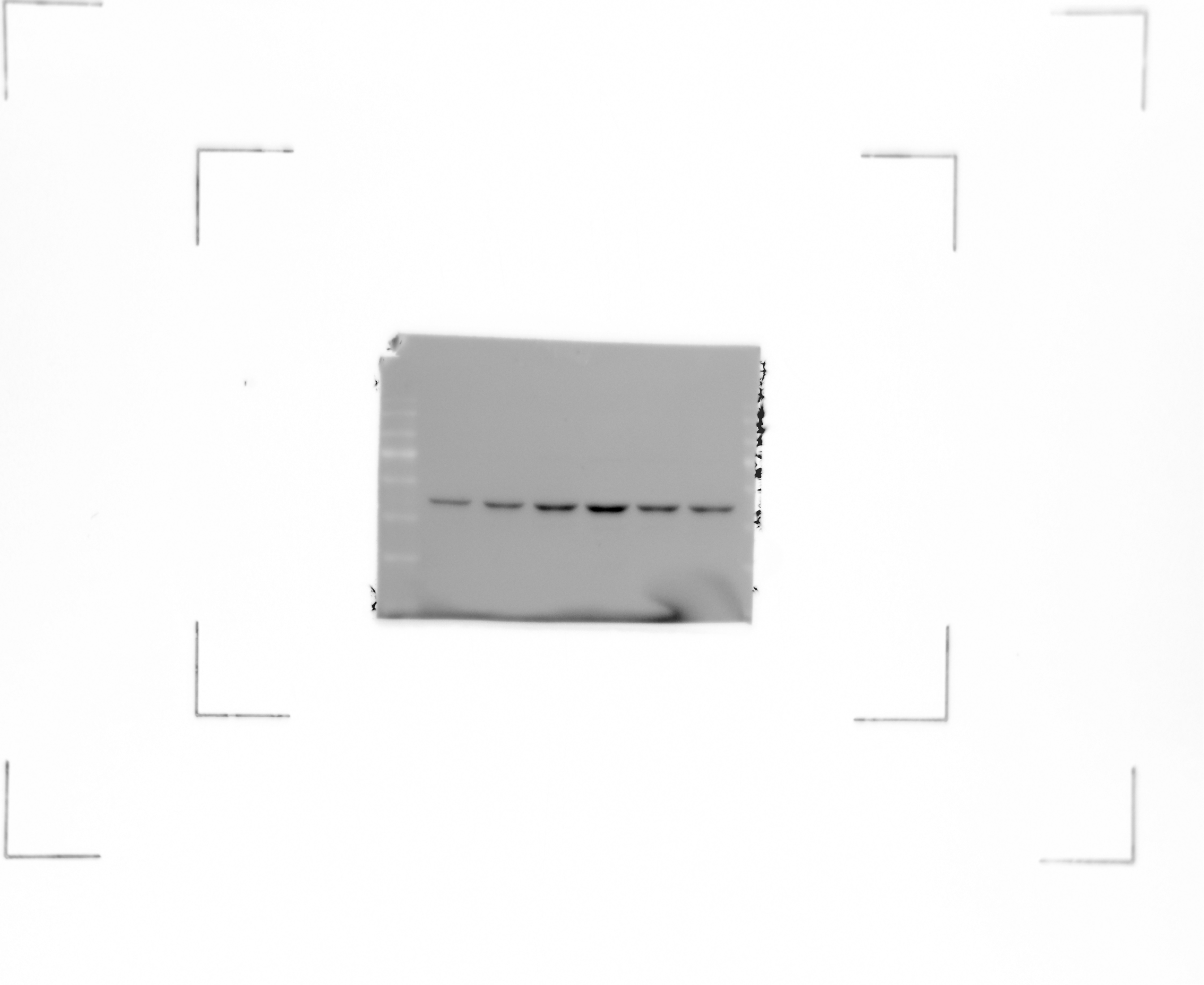

Supplement: Supplementary file 1 — Supplementary Material 1. [file 40001_2024_1968_MOESM1_ESM.zip › western blot original images/original images for all western blots/FIGURES4-6/Caspase 8-1.tif]

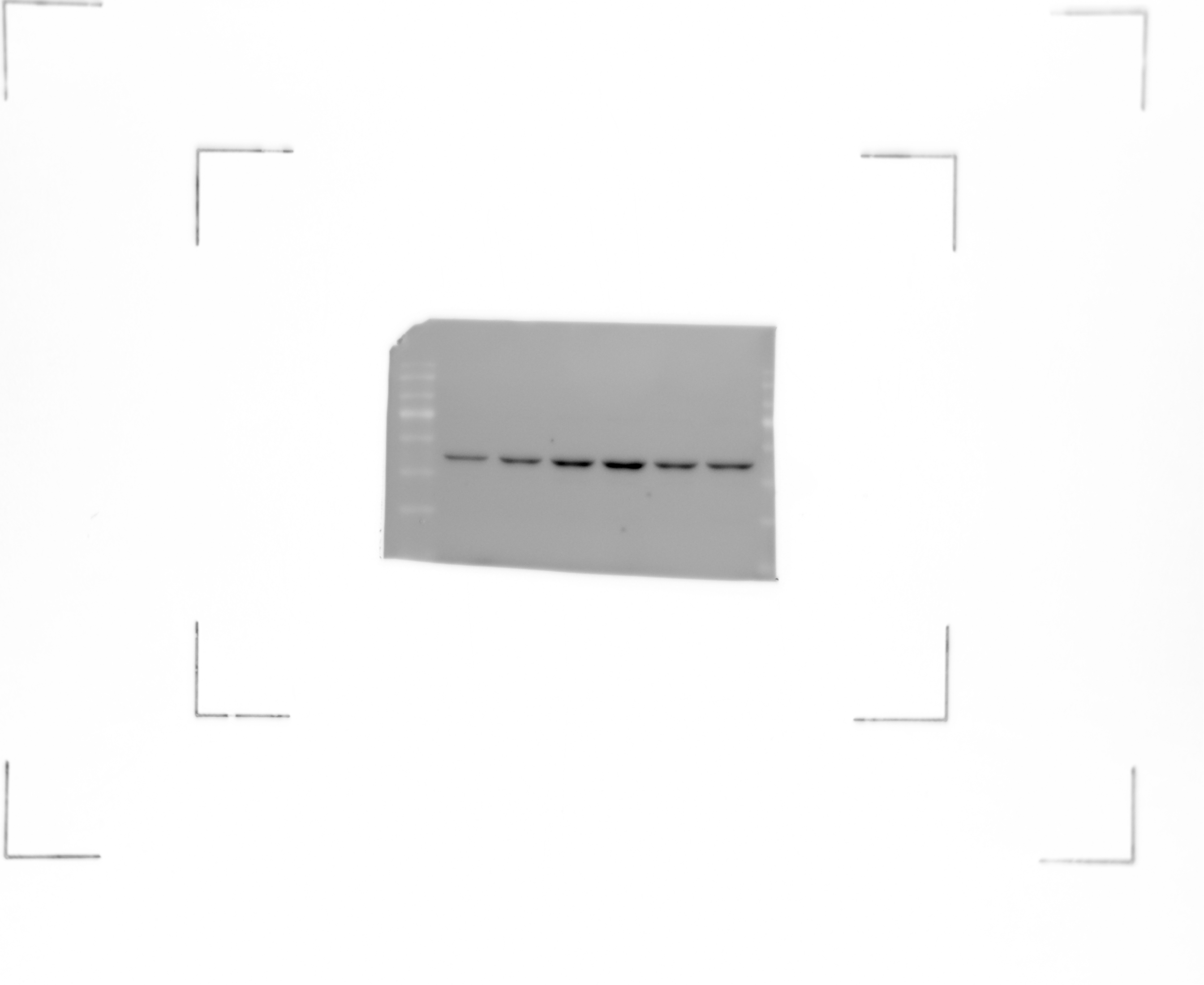

Supplement: Supplementary file 1 — Supplementary Material 1. [file 40001_2024_1968_MOESM1_ESM.zip › western blot original images/original images for all western blots/FIGURES4-6/Caspase 8-2.tif]

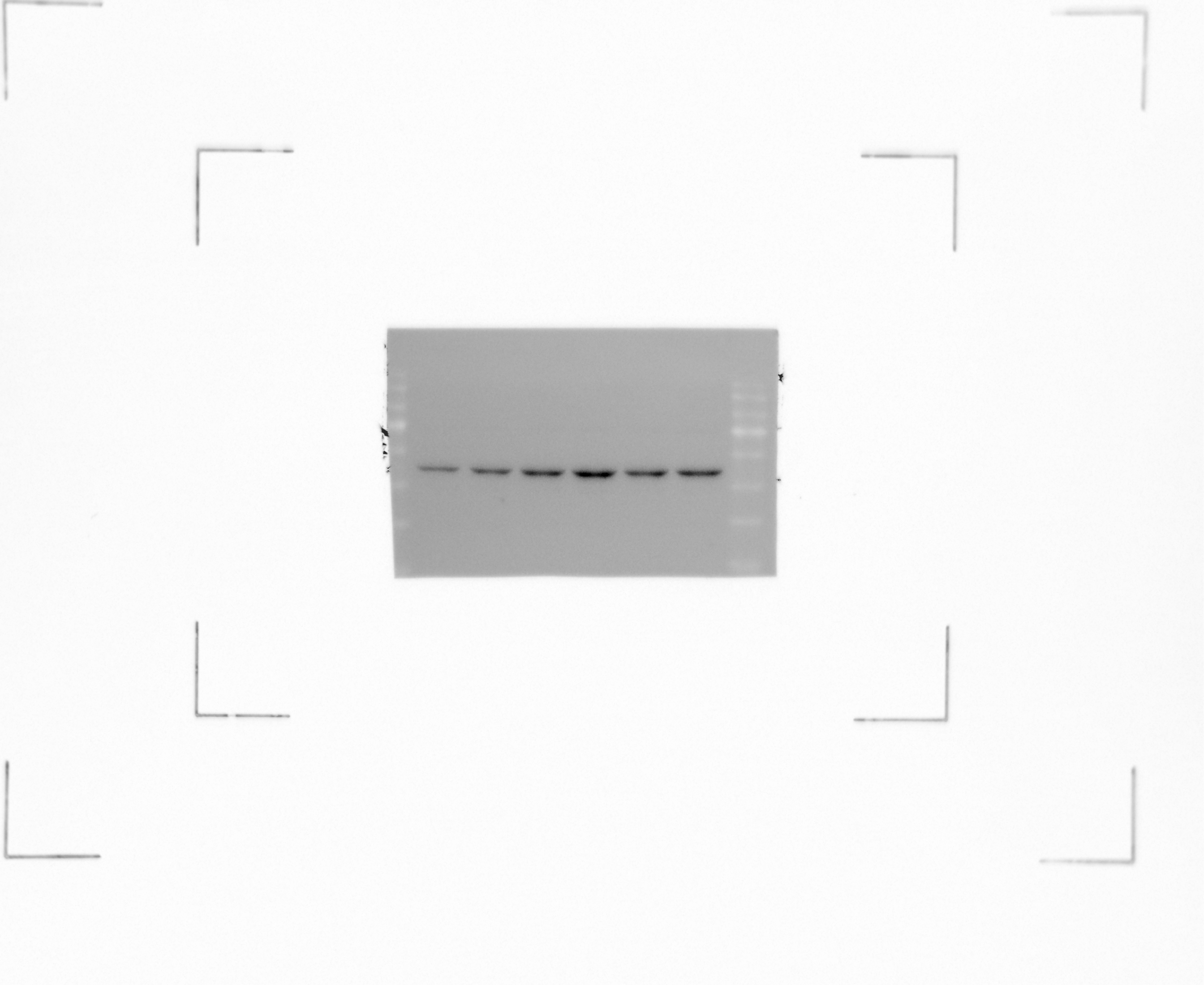

Supplement: Supplementary file 1 — Supplementary Material 1. [file 40001_2024_1968_MOESM1_ESM.zip › western blot original images/original images for all western blots/FIGURES4-6/Caspase 8-3.tif]

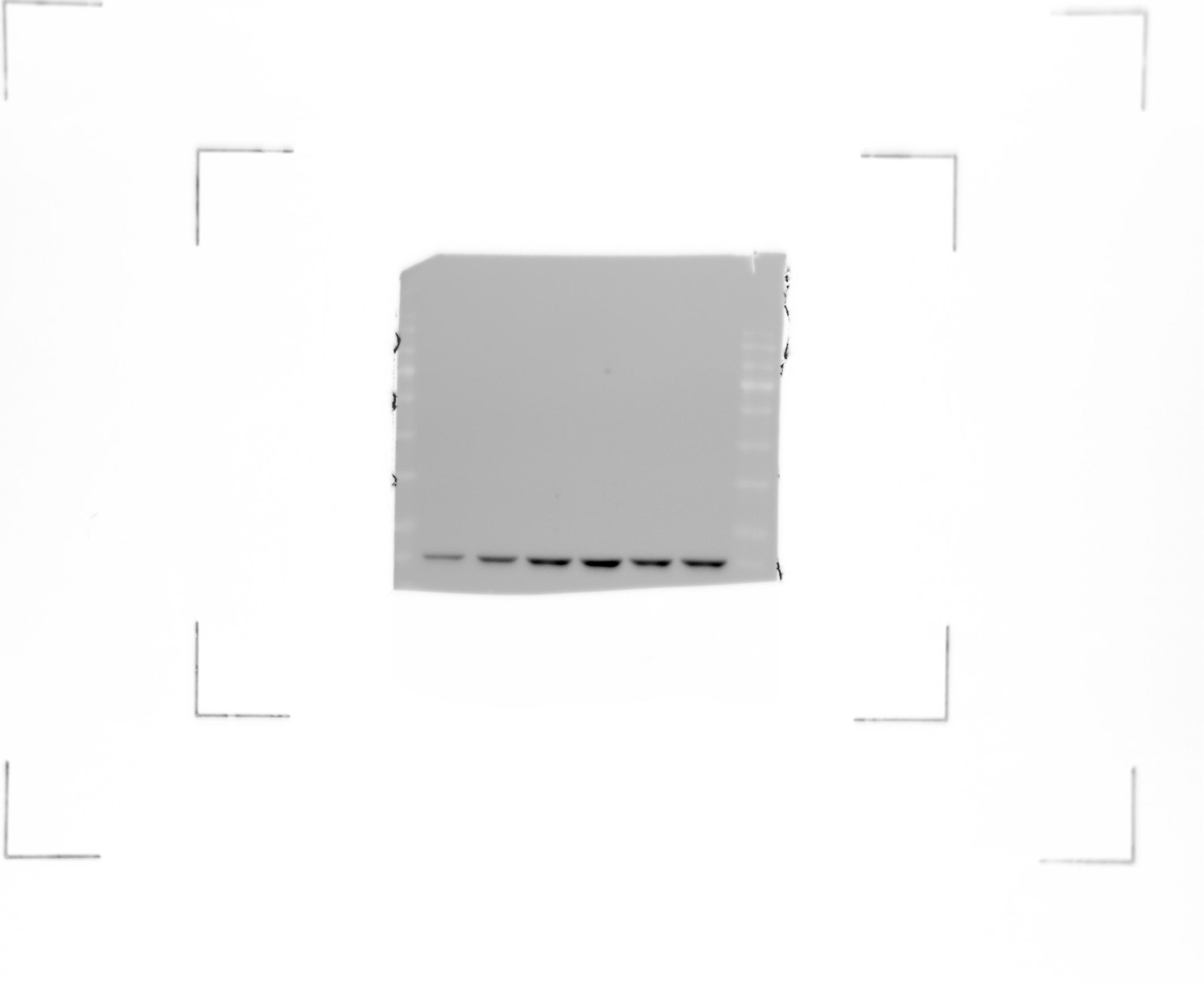

Supplement: Supplementary file 1 — Supplementary Material 1. [file 40001_2024_1968_MOESM1_ESM.zip › western blot original images/original images for all western blots/FIGURES4-6/Cleaved Caspase 3-1.tif]

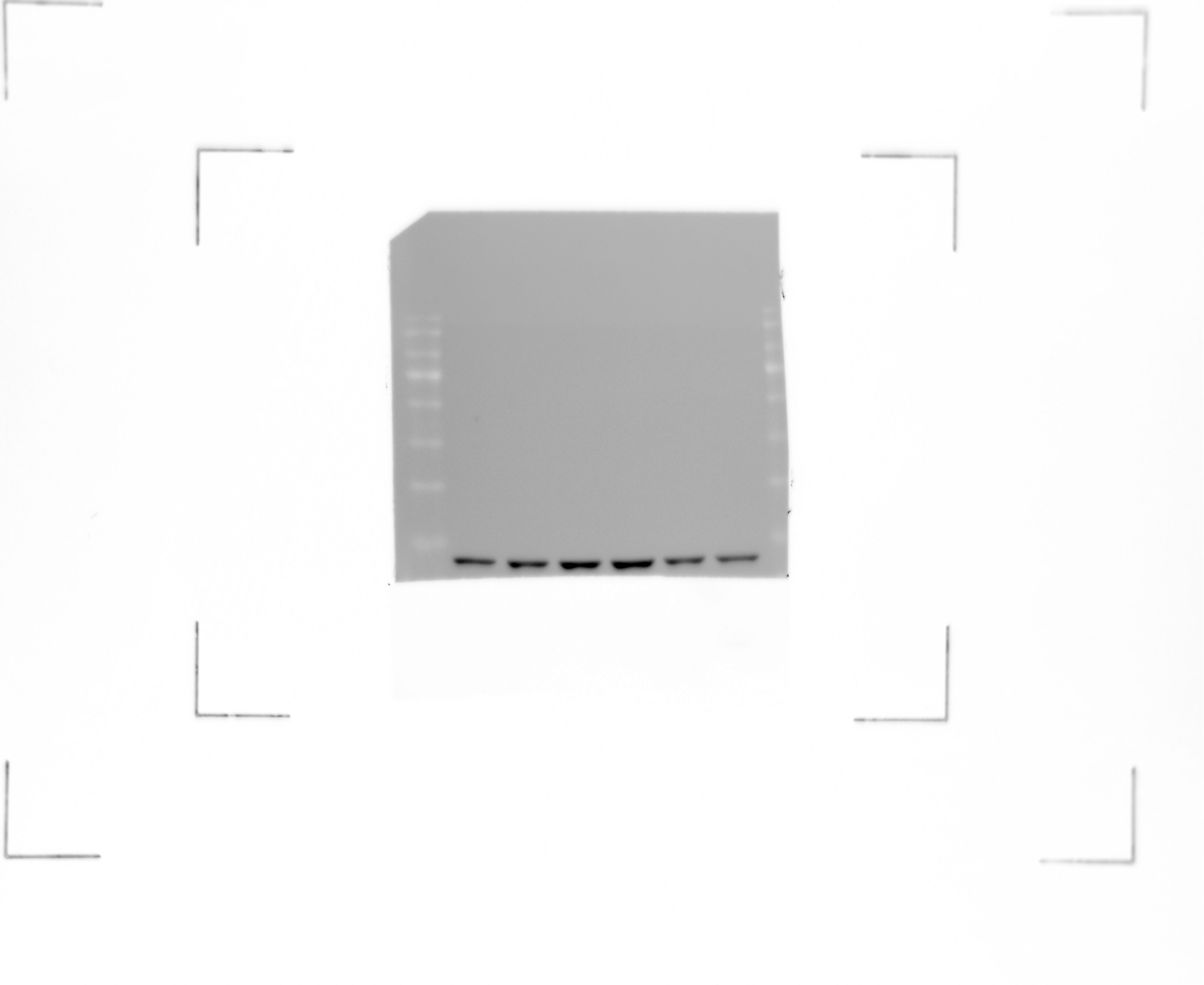

Supplement: Supplementary file 1 — Supplementary Material 1. [file 40001_2024_1968_MOESM1_ESM.zip › western blot original images/original images for all western blots/FIGURES4-6/Cleaved Caspase 3-2.tif]

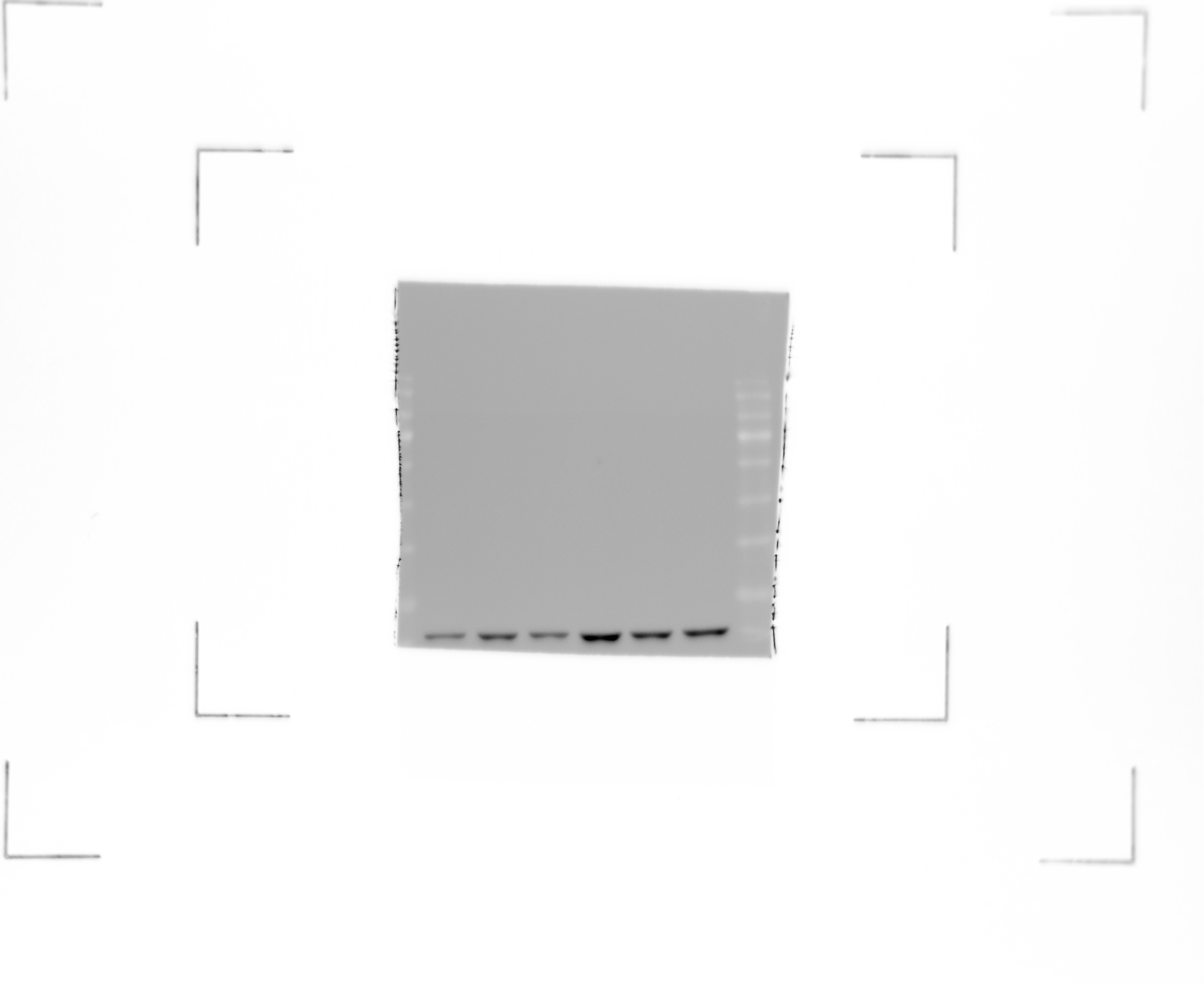

Supplement: Supplementary file 1 — Supplementary Material 1. [file 40001_2024_1968_MOESM1_ESM.zip › western blot original images/original images for all western blots/FIGURES4-6/Cleaved Caspase 3-3.tif]

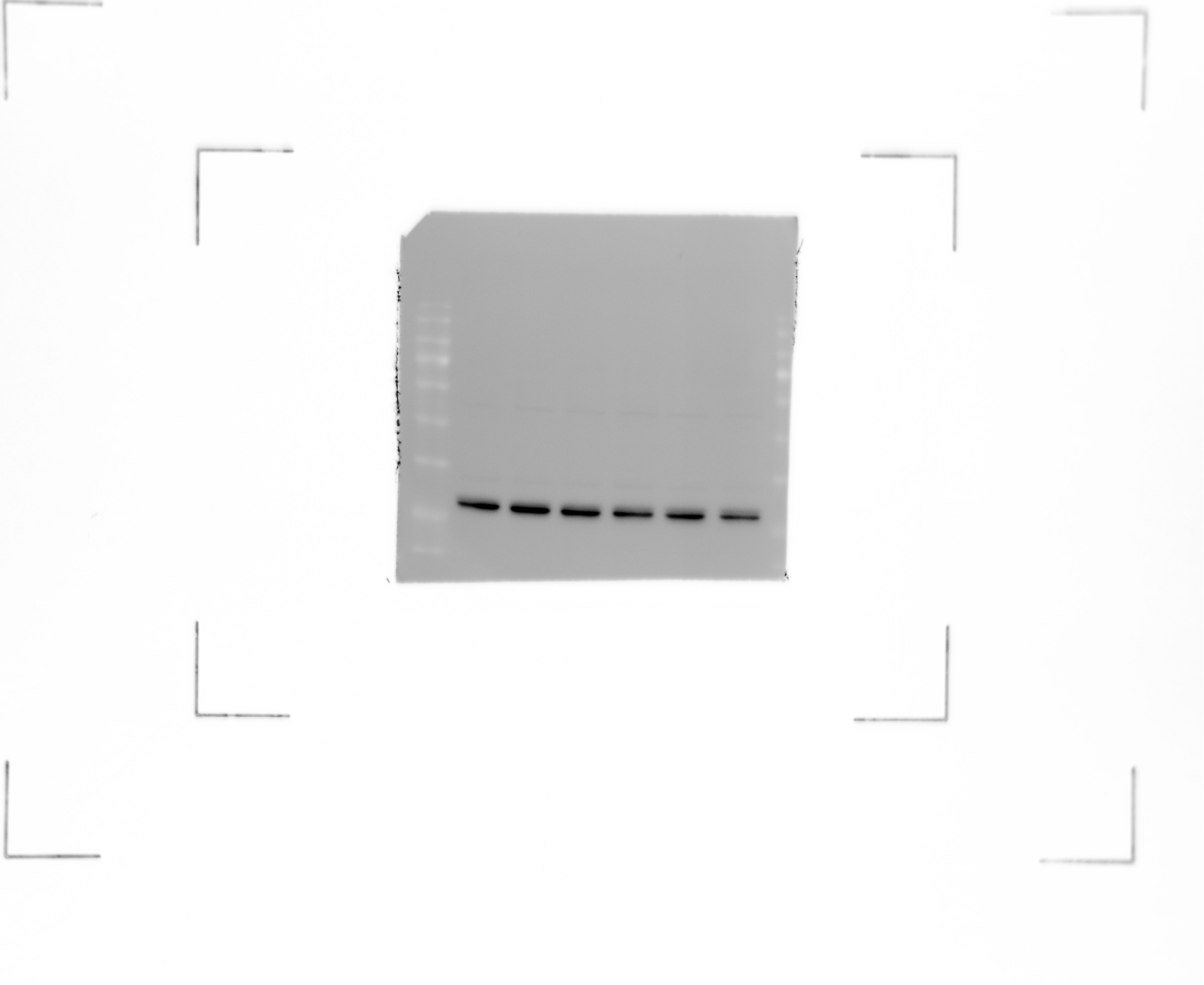

Supplement: Supplementary file 1 — Supplementary Material 1. [file 40001_2024_1968_MOESM1_ESM.zip › western blot original images/original images for all western blots/FIGURES4-6/FADD-1.tif]

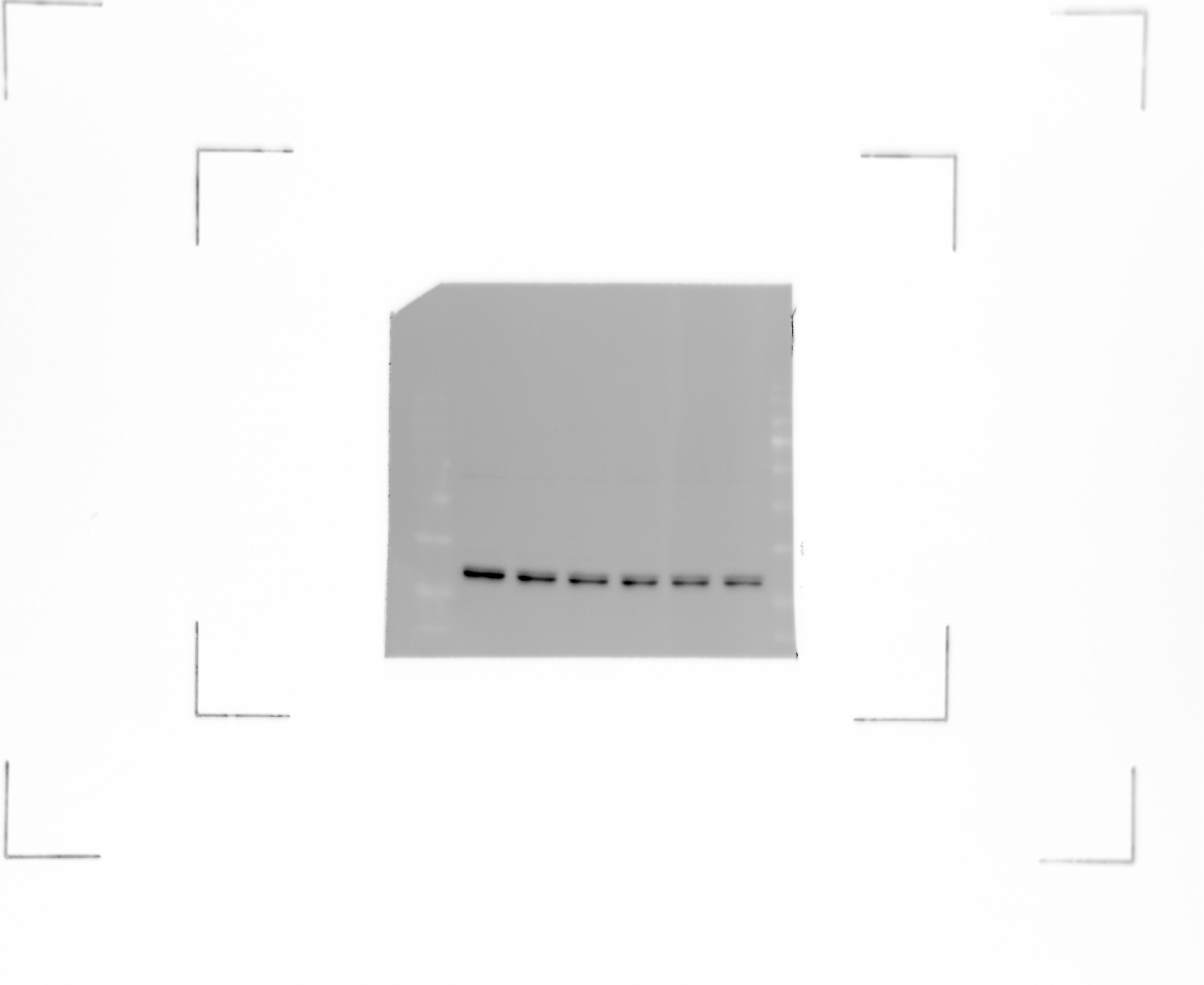

Supplement: Supplementary file 1 — Supplementary Material 1. [file 40001_2024_1968_MOESM1_ESM.zip › western blot original images/original images for all western blots/FIGURES4-6/FADD-2.tif]

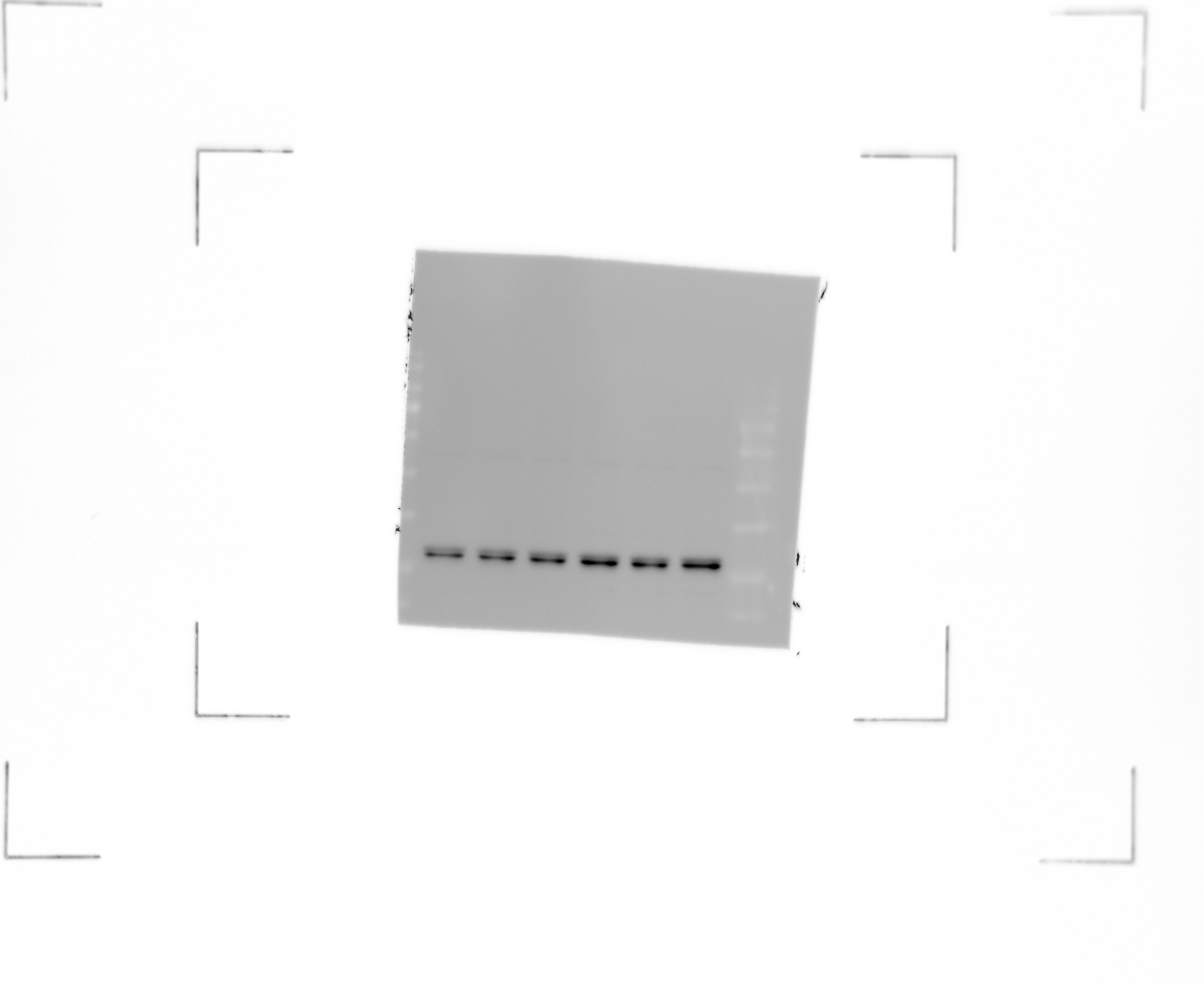

Supplement: Supplementary file 1 — Supplementary Material 1. [file 40001_2024_1968_MOESM1_ESM.zip › western blot original images/original images for all western blots/FIGURES4-6/FADD-3.tif]

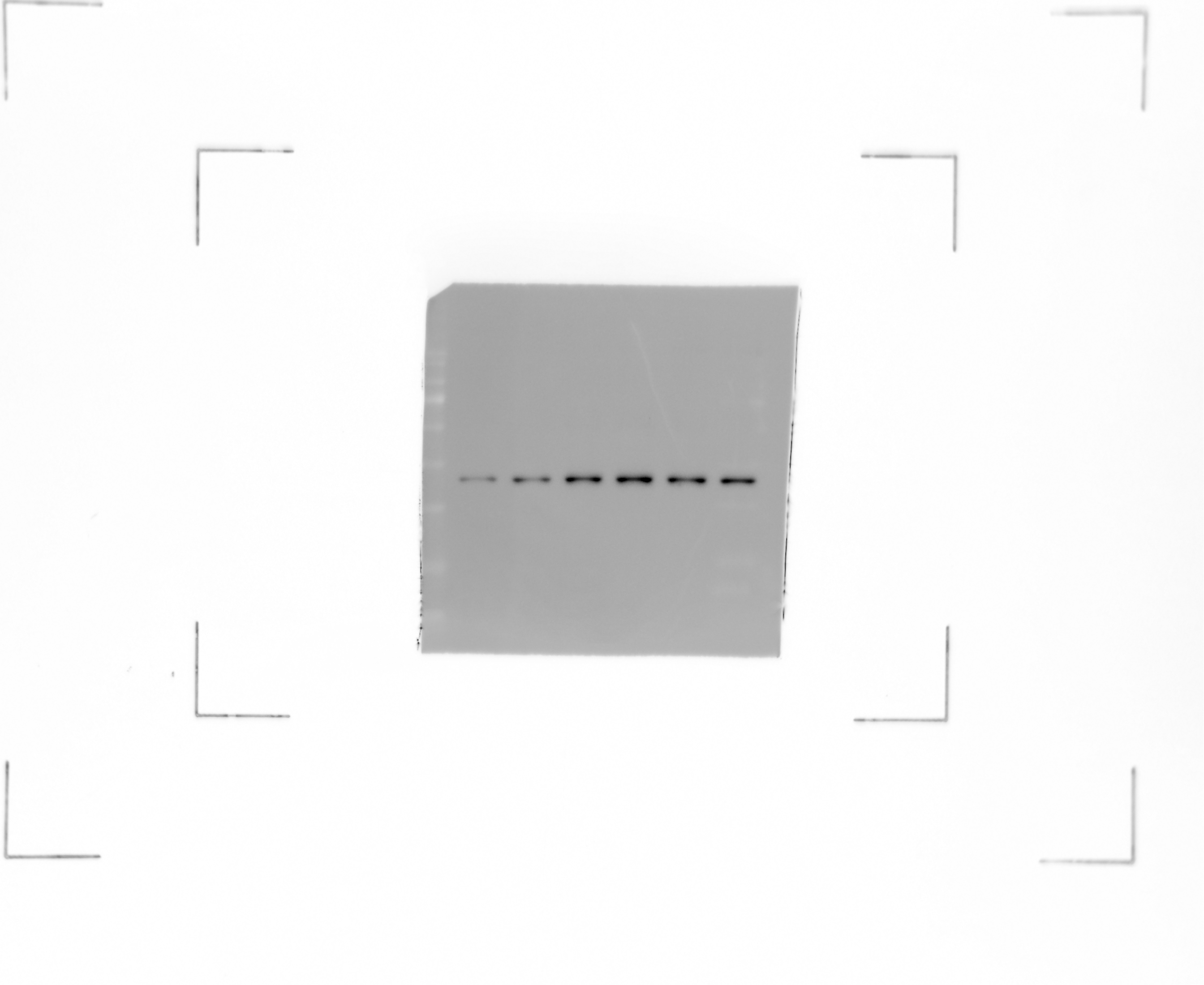

Supplement: Supplementary file 1 — Supplementary Material 1. [file 40001_2024_1968_MOESM1_ESM.zip › western blot original images/original images for all western blots/FIGURES4-6/FAS-1.tif]

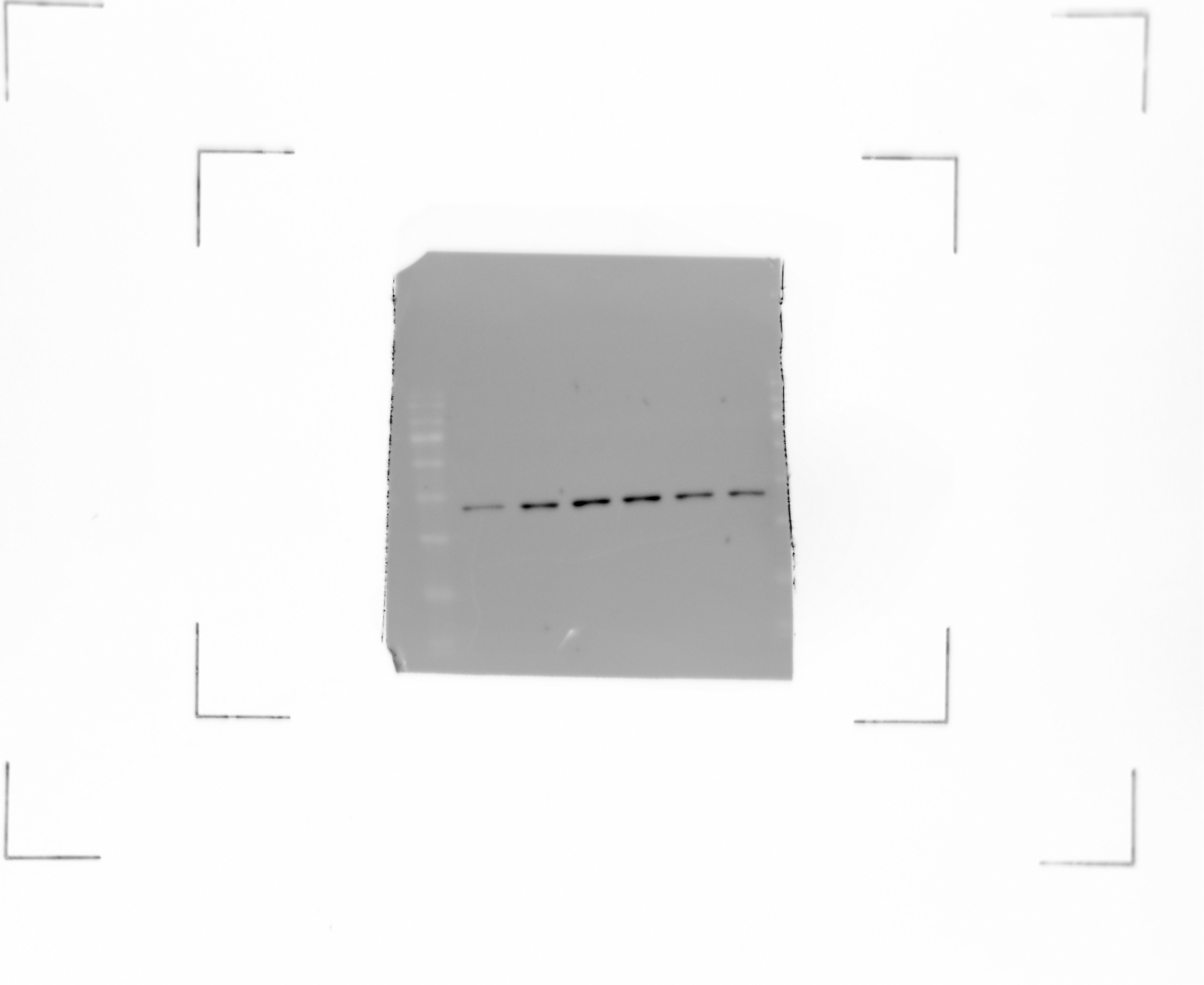

Supplement: Supplementary file 1 — Supplementary Material 1. [file 40001_2024_1968_MOESM1_ESM.zip › western blot original images/original images for all western blots/FIGURES4-6/FAS-2.tif]

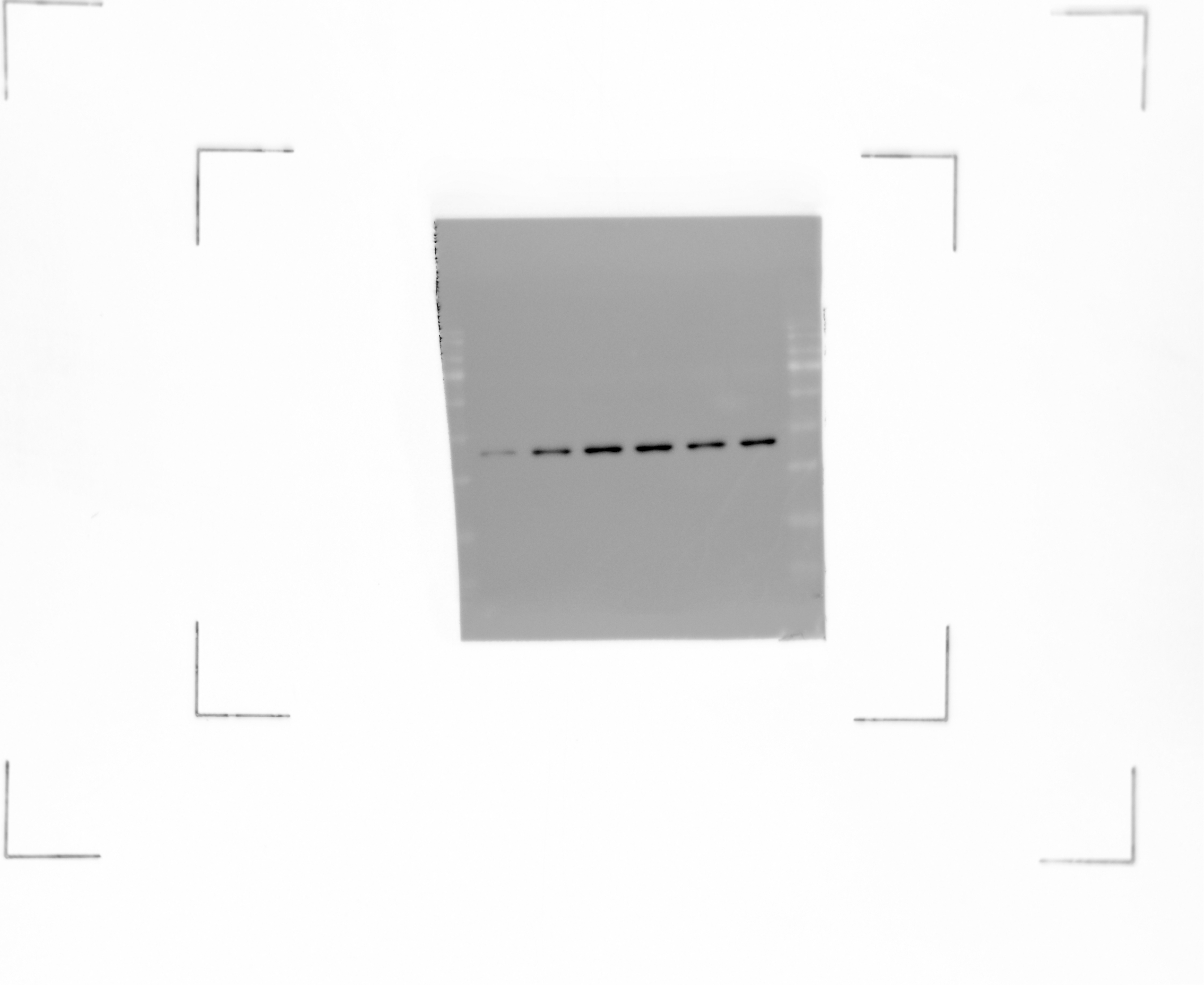

Supplement: Supplementary file 1 — Supplementary Material 1. [file 40001_2024_1968_MOESM1_ESM.zip › western blot original images/original images for all western blots/FIGURES4-6/FAS-3.tif]

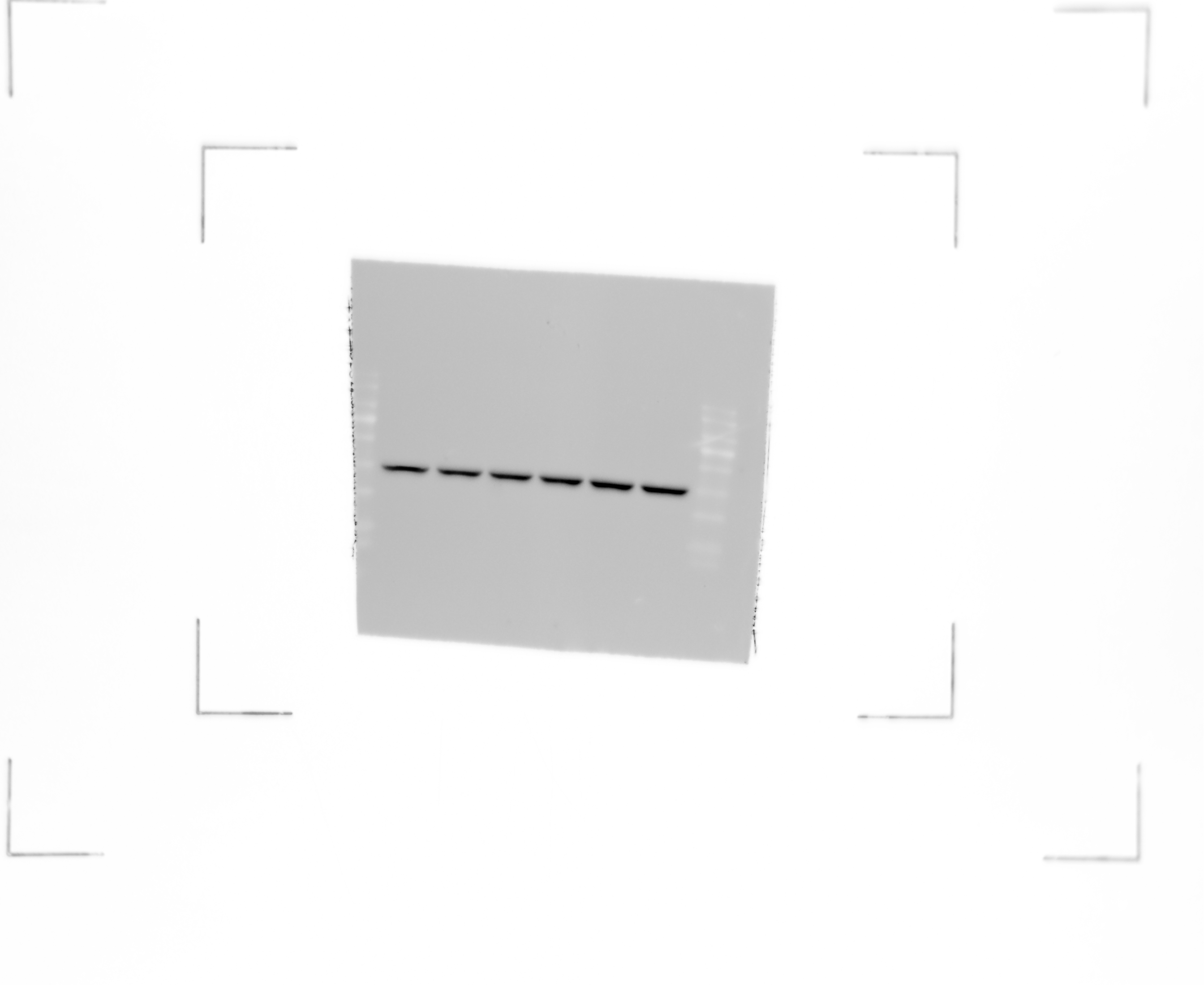

Supplement: Supplementary file 1 — Supplementary Material 1. [file 40001_2024_1968_MOESM1_ESM.zip › western blot original images/original images for all western blots/FIGURES4-6/GAPDH-1.tif]

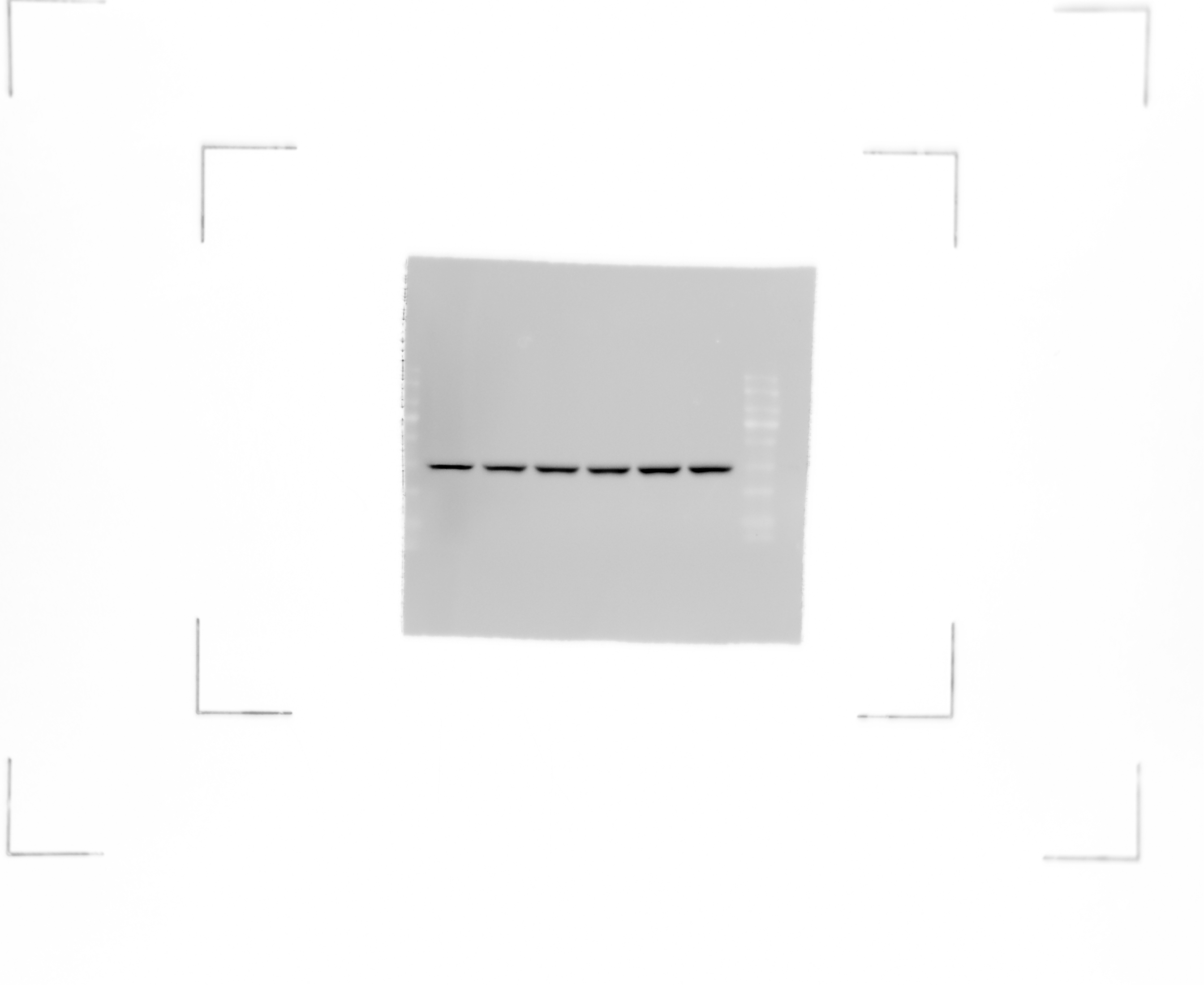

Supplement: Supplementary file 1 — Supplementary Material 1. [file 40001_2024_1968_MOESM1_ESM.zip › western blot original images/original images for all western blots/FIGURES4-6/GAPDH-2.tif]

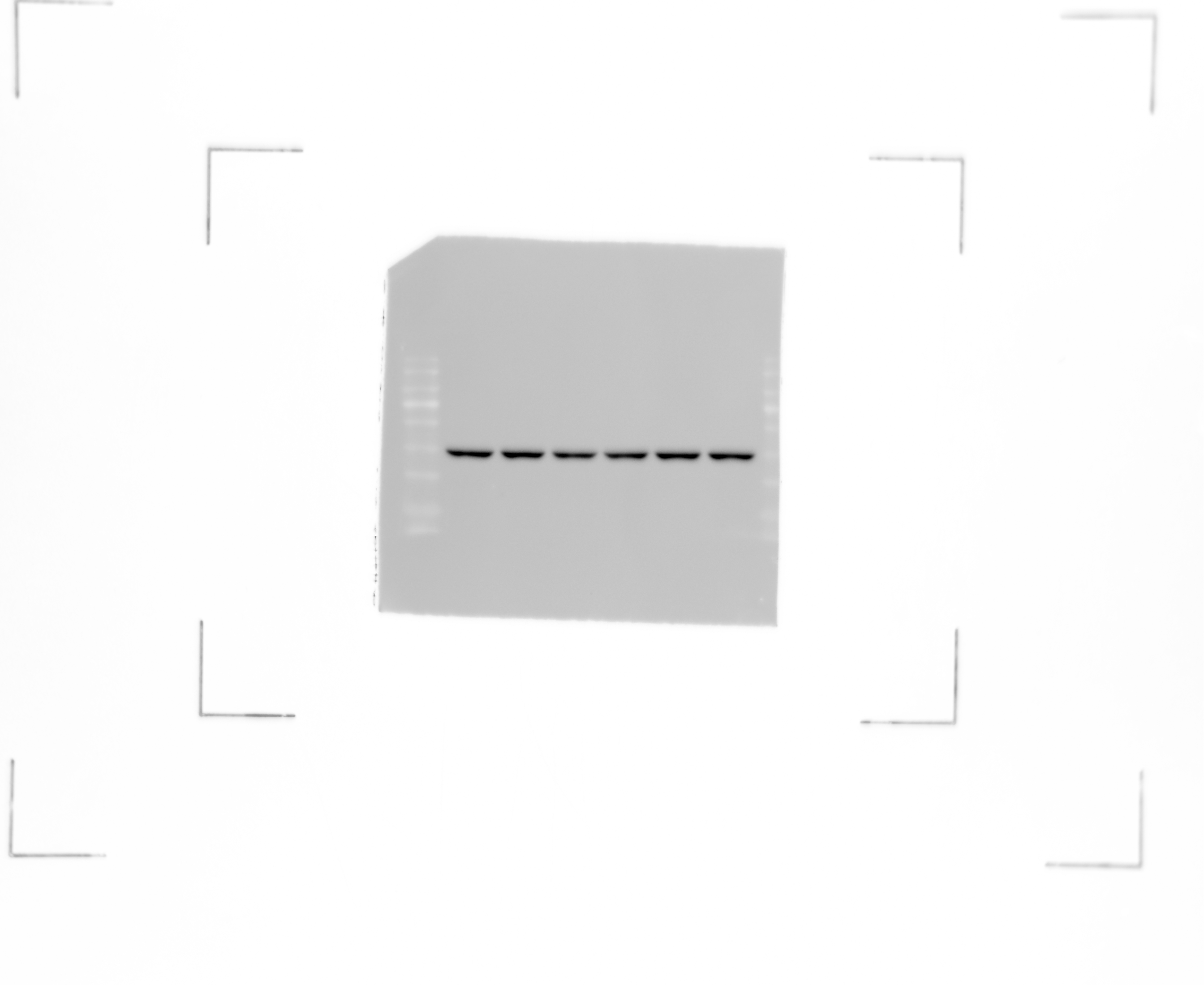

Supplement: Supplementary file 1 — Supplementary Material 1. [file 40001_2024_1968_MOESM1_ESM.zip › western blot original images/original images for all western blots/FIGURES4-6/GAPDH-3.tif]

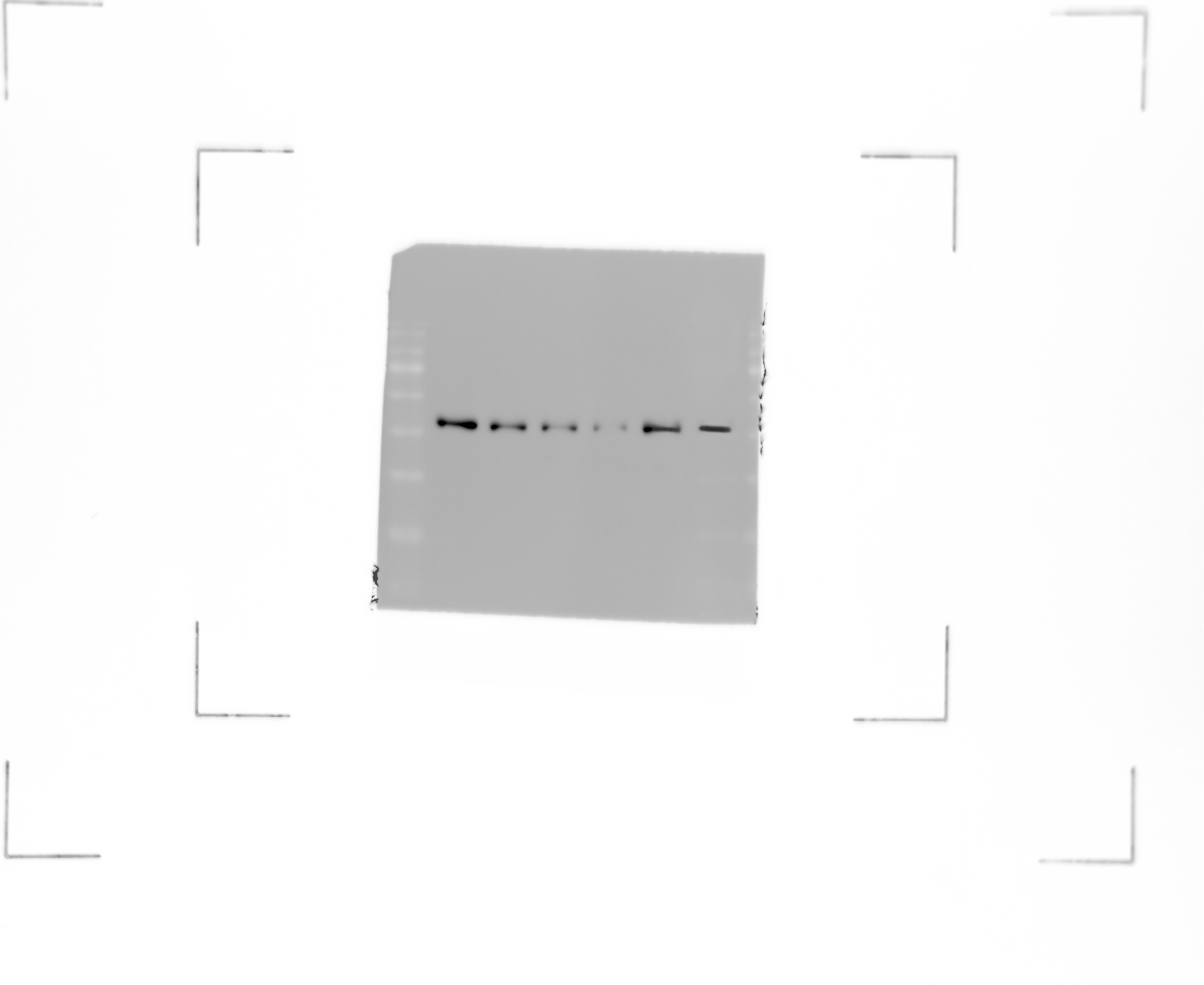

Supplement: Supplementary file 1 — Supplementary Material 1. [file 40001_2024_1968_MOESM1_ESM.zip › western blot original images/original images for all western blots/FIGURES4-6/KRT8-1.tif]

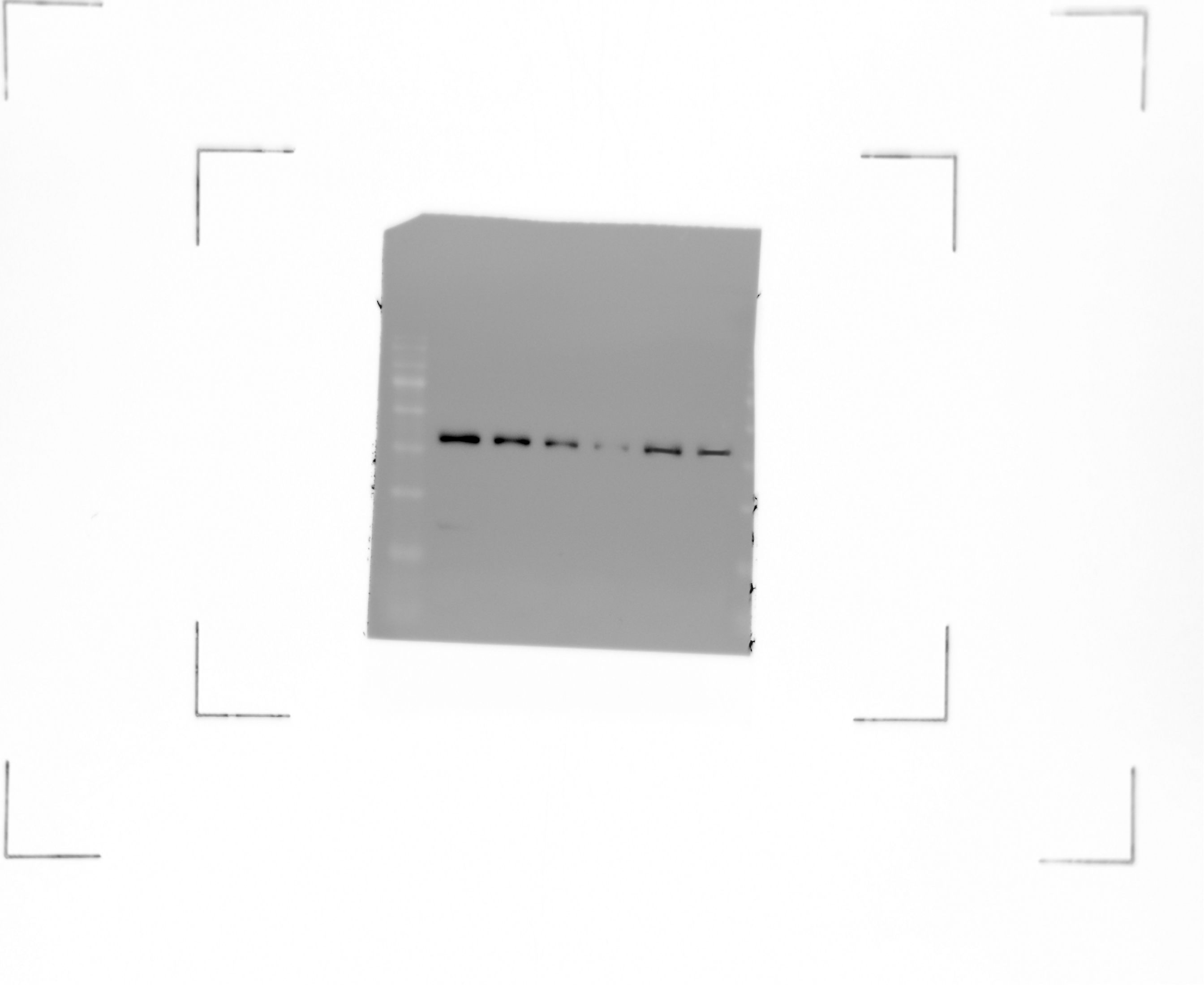

Supplement: Supplementary file 1 — Supplementary Material 1. [file 40001_2024_1968_MOESM1_ESM.zip › western blot original images/original images for all western blots/FIGURES4-6/KRT8-2.tif]

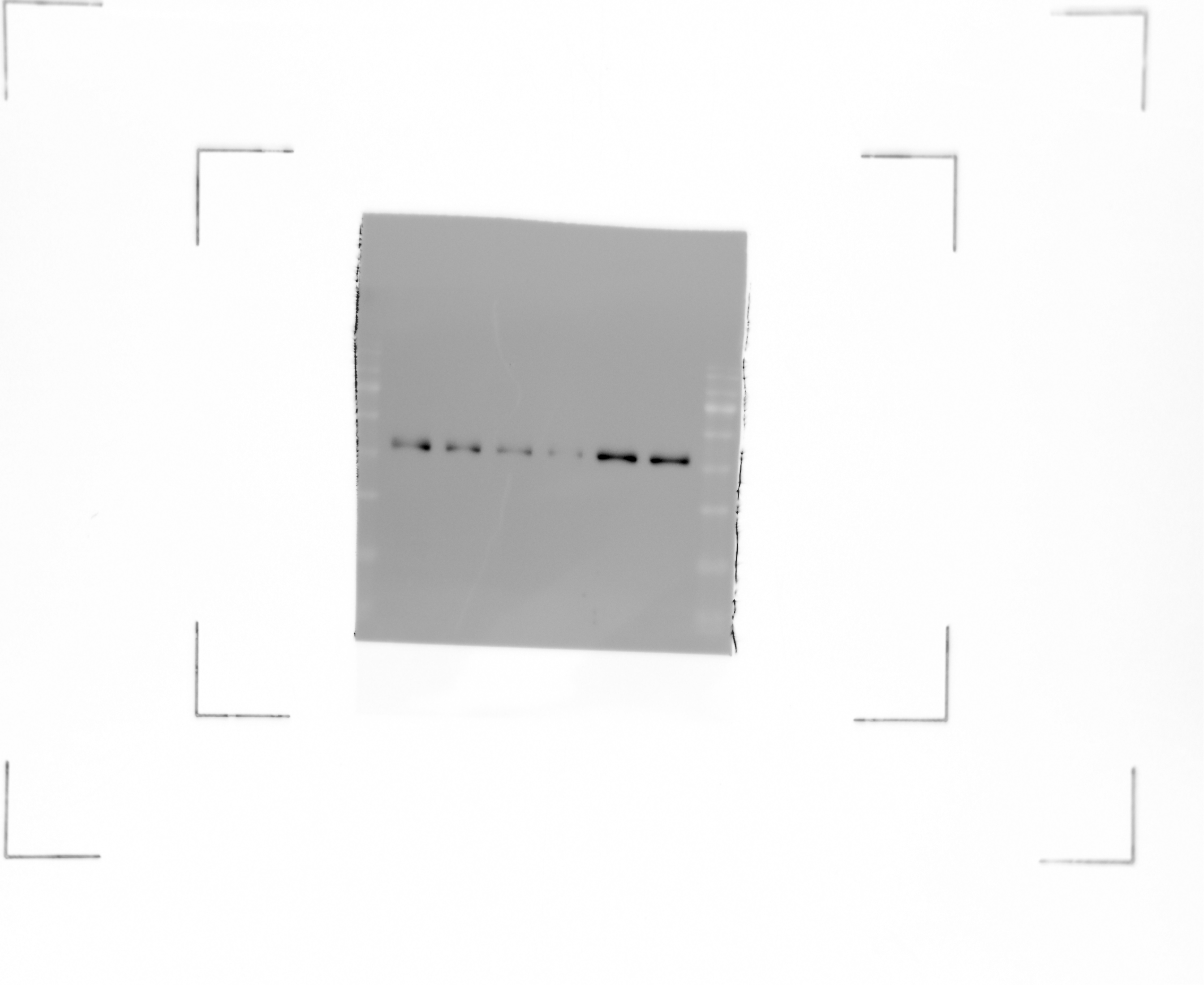

Supplement: Supplementary file 1 — Supplementary Material 1. [file 40001_2024_1968_MOESM1_ESM.zip › western blot original images/original images for all western blots/FIGURES4-6/KRT8-3.tif]

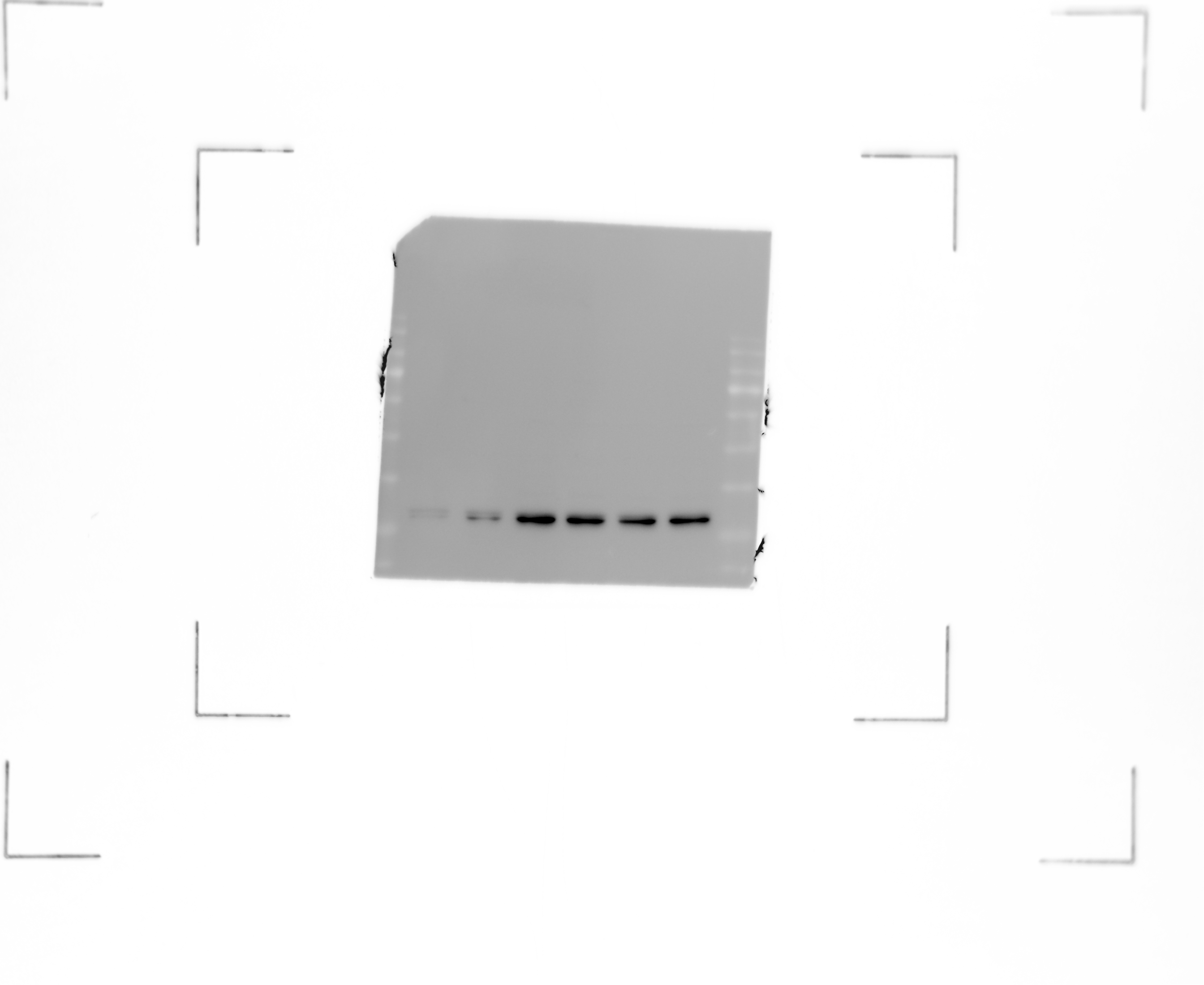

Supplement: Supplementary file 1 — Supplementary Material 1. [file 40001_2024_1968_MOESM1_ESM.zip › western blot original images/original images for all western blots/FIGURES4-6/p-FADD-1.tif]

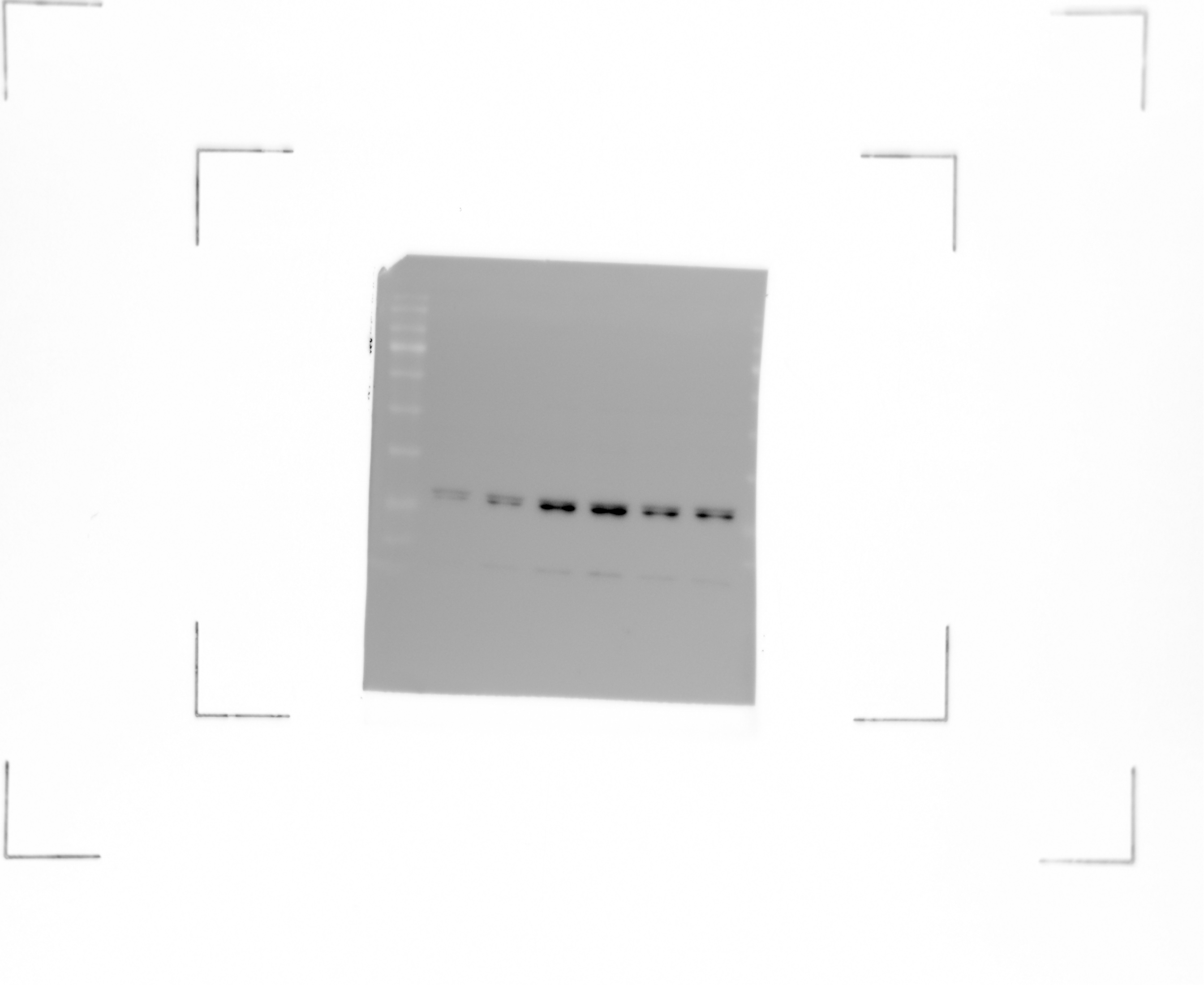

Supplement: Supplementary file 1 — Supplementary Material 1. [file 40001_2024_1968_MOESM1_ESM.zip › western blot original images/original images for all western blots/FIGURES4-6/p-FADD-2.tif]

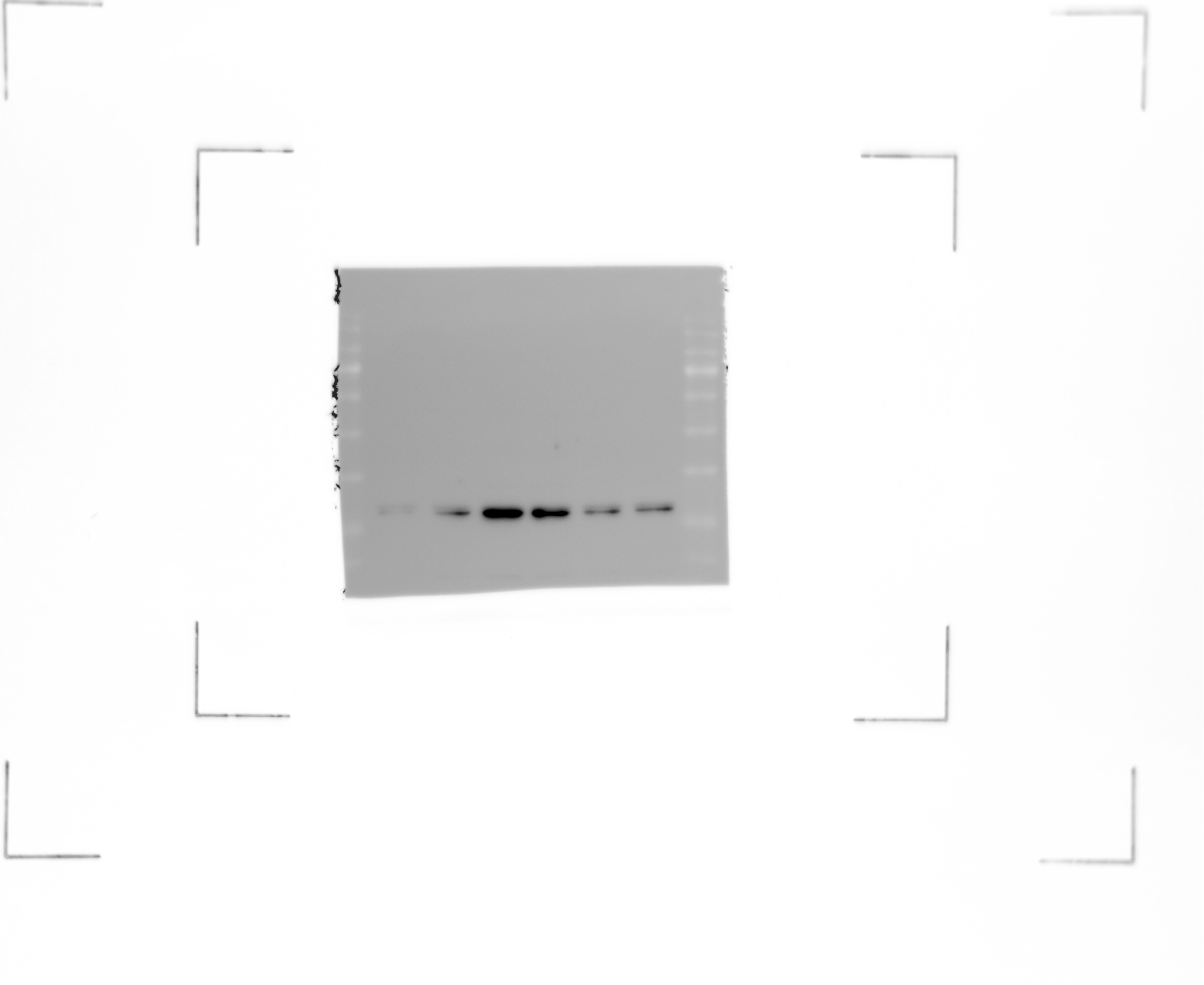

Supplement: Supplementary file 1 — Supplementary Material 1. [file 40001_2024_1968_MOESM1_ESM.zip › western blot original images/original images for all western blots/FIGURES4-6/p-FADD-3.tif]
